# A Pooled shRNA Screen Identifies Rbm15, Spen, and Wtap as Factors Required for Xist RNA-Mediated Silencing

**DOI:** 10.1016/j.celrep.2015.06.053

**Published:** 2015-07-16

**Authors:** Benoit Moindrot, Andrea Cerase, Heather Coker, Osamu Masui, Anne Grijzenhout, Greta Pintacuda, Lothar Schermelleh, Tatyana B. Nesterova, Neil Brockdorff

**Affiliations:** 1 Developmental Epigenetics, Department of Biochemistry, University of Oxford, South Parks Road, Oxford, OX1 3QU, UK; 2 Advanced Cellular Imaging, Department of Biochemistry, University of Oxford, South Parks Road, Oxford, OX1 3QU, UK; 3 Laboratory for Developmental Genetics, RIKEN Center for Integrative Medical Sciences, 1-7-22 Suehiro, Tsurumi-ku, Yokohama 230-0045, Japan

## Abstract

X-chromosome inactivation is the process that evolved in mammals to equalize levels of X-linked gene expression in XX females relative to XY males. Silencing of a single X chromosome in female cells is mediated by the non-coding RNA Xist. Although progress has been made toward identifying factors that function in the maintenance of X inactivation, the primary silencing factors are largely undefined. We developed an shRNA screening strategy to produce a ranked list of candidate primary silencing factors. Validation experiments performed on several of the top hits identified the SPOC domain RNA binding proteins Rbm15 and Spen and Wtap, a component of the m6A RNA methyltransferase complex, as playing an important role in the establishment of Xist-mediated silencing. Localization analysis using super-resolution 3D-SIM microscopy demonstrates that these factors co-localize with Xist RNA within the nuclear matrix subcompartment, consistent with a direct interaction.

## Highlights

• An shRNA screen identifies factors implicated in chromosome silencing by Xist RNA
• Rbm15, Wtap, and Spen are required for Xist-mediated silencing
• Rbm15 is important for efficient deposition of H3K27me3 on the inactive chromosome
• Rbm15, Wtap, and Spen co-localize with Xist RNA in perichromatin spaces

## Graphical Abstract

**Figure:**
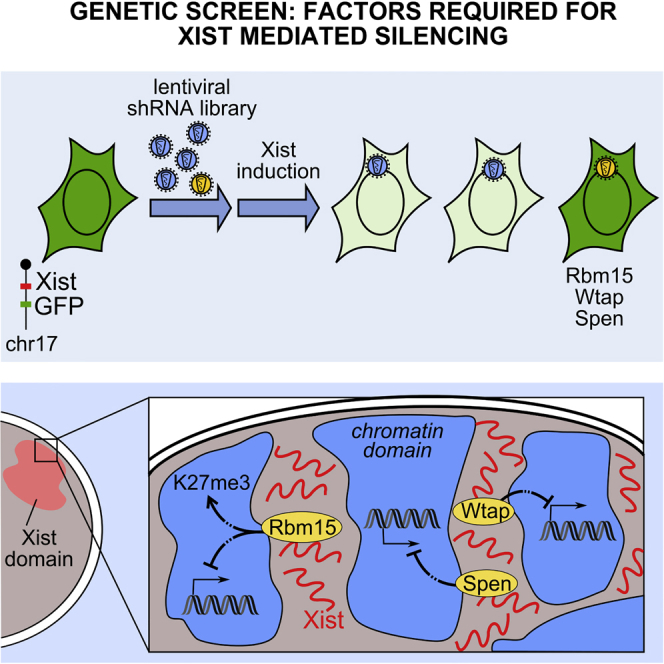

## Teaser

To identify primary silencing factors implicated in X-chromosome inactivation, Moindrot et al. set up a pooled shRNA genetic screen. The RNA-binding proteins Rbm15 and Spen, together with Wtap, a subunit of the RNA methylation complex, were identified as important factors required for Xist-mediated silencing.

## Introduction

Dosage compensation in mammals is achieved through the stable silencing of one of the two X chromosomes in female cells during early development, a process termed X-chromosome inactivation (XCI). XCI is initiated by the X inactive specific transcript (Xist), a long non-coding RNA (lncRNA) that is expressed from the inactive X (Xi) elect. Xist RNA spreads in *cis* over the length of the chromosome, and its accumulation triggers the formation of a stable heterochromatic structure, the Barr body (reviewed in Gendrel and Heard, 2014). Xist is both necessary and sufficient to initiate the X-inactivation process (Penny et al., 1996, Lee et al., 1996, Herzing et al., 1997), and indeed Xist transgenes function efficiently when located on autosomes (Lee et al., 1996, Wutz and Jaenisch, 2000). Xist functions in a context-dependent manner during a restricted window of opportunity in early development (Wutz and Jaenisch, 2000; Savarese et al., 2006). Functional dissection of Xist RNA has identified a critical element, the A repeat, required for chromosome inactivation, and multiple redundant elements that mediate localization in *cis* (Wutz et al., 2002).

Formation of the Barr body is a multistep process involving several pathways linked to formation of repressive heterochromatin. Notable examples are the acquisition or loss of specific histone tail modifications, enrichment or depletion of variant histones, DNA methylation at X-linked promoter CpG islands, and long-range topological reorganization of the chromatin fiber. How these different pathways are linked to Xist RNA accumulation and to one another remains poorly understood. A key challenge has been to identify the primary factors that initiate the silencing cascade during the early developmental window of opportunity. A priori, these factors are predicted to include one or more RNA binding proteins (RBPs). Polycomb repressive complex 2 (PRC2), which mediates the histone modification H3 lysine 27 methylation, has been suggested as a candidate for this role (Silva et al., 2003; Plath et al., 2003) and has been proposed to bind directly to Xist RNA (Zhao et al., 2008). However, recent evidence indicates that PRC2 and Xist RNA are spatially separated (Cerase et al., 2014) and further that PRC2 recruitment is mediated by a region of the transcript that is separate from the critical A-repeat element (da Rocha et al., 2014). Two other candidate factors that have been identified are the nuclear matrix proteins SATB1 and the RBP SAFA/hnRNPU. SATB1 has been suggested to have an indirect role in conferring competence for Xist silencing within the developmental window of opportunity (Agrelo et al., 2009). SAFA/hnRNPU, on the other hand, has been suggested to bind directly to Xist RNA via its RRM domain and to facilitate Xist RNA localization (Hasegawa et al., 2010). A role for the nuclear matrix in Xist-mediated silencing is further indicated by the observation that Xist RNA domains are retained in nuclear matrix preparations (Clemson et al., 1996). Moreover, imaging of Xist RNA by super-resolution 3D-SIM microscopy (SR-3DSIM) demonstrates localization within perichromatin spaces, together with SAFA/hnRNPU (Smeets et al., 2014).

Efforts to purify native Xist ribonucleoprotein complexes have been hampered by the association of Xist RNA with the insoluble nuclear matrix. Similarly, an attempt to purify key factors from nuclear extracts using defined regions of Xist identified only hnRNPC, a generic mRNA processing factor (Brown and Baldry, 1996). Two independent studies have applied RNAi-based genetic screening to identify factors involved in X inactivation in XX somatic cells (Chan et al., 2011; Bhatnagar et al., 2014). Both studies identified several candidates, and in some cases, validation experiments supported a role in X inactivation and/or Xist expression. However, none of the validated candidates was a known RBP. In this study, we set out to identify the primary silencing factors that act in the critical developmental window of opportunity using a pooled shRNA genetic screen. Several of the targets that we identified were related, either as components of the same multisubunit complexes or defined pathways, suggesting that the screen achieved a high degree of saturation. We validated several top-ranking hits, of which Rbm15 and Spen, two related RBPs, and Wtap a subunit of the N6-methyladenosine (m6A) RNA methylation complex, were all found to play a role in Xist-mediated silencing. SR-3DSIM analysis of Rbm15, Spen, and Wtap demonstrated co-localization with Xist in nuclear matrix/perichromatin spaces, indicating that these factors may interact directly with Xist RNA.

## Results

### Pooled shRNA Screening

**Figure 1:**
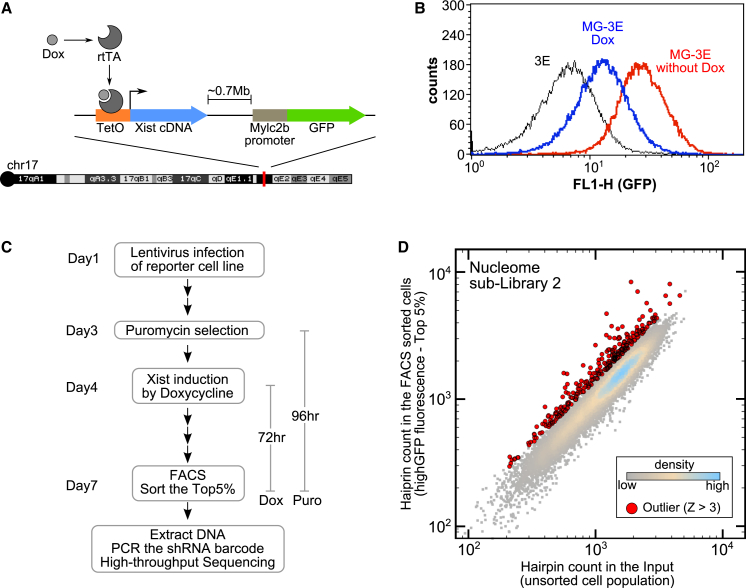
A Genetic Screen to Identify Silencing Factors Acting during the Establishment of XCI (A) Arrangement of the engineered chromosome 17 loci in the MG-3E reporter cell line. See also Figure S1. (B) GFP fluorescence of MG-3E cell line before (red) and after (blue) doxycycline treatment (72 hr, 1.5 μg/ml). The parental 3E cell line (black) is also shown. (C) Workflow for the genetic screen. MG-3E cells were transduced with shRNA libraries 3 days prior to Xist induction. Transduced cells were FACS sorted 72 hr after Xist induction. PCR across the shRNA barcode is performed on DNA from sorted and unsorted MG-3E cells and PCR products analyzed by HTS. (D) Hairpin count in unsorted and sorted MG-3E cells transduced with one of the three nucleome sublibraries. The outliers enriched in the sorted samples are indicated in red (z ≥ 3).

In order to identify factors that function in the critical developmental window of opportunity, we established a reporter system in mouse embryonic stem cells (mESCs), which are known to be Xist responsive (Wutz and Jaenisch, 2000; Tang et al., 2010; Cerase et al., 2014). We made use of an XY mESC cell line, 3E, in which a doxycycline-inducible Xist transgene is located on chromosome 17 (Tang et al., 2010; Cerase et al., 2014). An unstable PEST-GFP open reading frame (Corish and Tyler-Smith, 1999) was inserted under the control of Mylc2b promoter in *cis* with the Xist transgene using homologous recombination to generate the MG-3E cell line (Figures 1A and S1). The Mylc2b locus was selected as one of several loci that is efficiently silenced following 3 days of Xist RNA induction in undifferentiated 3E ESCs (Tang et al., 2010; Cerase et al., 2014). Consistent with expectations, MG-3E cells showed strongly reduced Mylc2b-GFP levels following induction of Xist RNA expression, as determined by fluorescence-activated cell sorting (FACS) analysis (Figure 1B).

We adopted a pooled lentiviral shRNA screening strategy (Silva et al., 2008; Sims et al., 2011) using a custom nucleome shRNA library comprising up to nine independent shRNA hairpins for each of 5,088 target genes encoding mouse proteins with the Gene Ontology (GO) term nucleus. Additionally, a pilot screen using a commercial whole genome shRNA library identified targets in the ubiquitylation pathway (data not shown), and we therefore performed a parallel experiment using a custom designed library of shRNAs directed at ∼1,000 target genes encoding mouse proteins with a function in ubiquitylation/sumolyation. A proportion of shRNAs were present in both ubiquitylome and nucleome libraries. Each shRNA was tagged with a unique barcode, enabling subsequent identification by high-throughput sequencing (HTS).

Following optimization and establishment of appropriate conditions, both libraries were screened to identify targets for which knockdown enhanced representation in GFP high cells following induction of Xist RNA (Figure 1C). Briefly, cells were transduced with lentiviral packaged shRNA pools of 9,000–15,000 shRNAs, at low multiplicity of infection (MOI), and then selected for puromycin resistance for 4 days. After the first 24 hr of puromycin selection, doxycycline was added to induce Xist RNA for a total of 3 days, at which point cells were harvested and FACS sorted based on high GFP fluorescence. The representation of shRNAs in FACS sorted and unsorted populations was determined by HTS of PCR products spanning the barcode (Figure 1D; Table S1). To identify putative Xist silencing factors, we established a pipeline to prioritize hits based first on the ratio of barcode sequence in sorted and unsorted populations and then, for a given factor, on the number of independent shRNAs overrepresented in the high GFP population (Figure S2A).

### Validation of shRNA Screen Hits

**Figure 2:**
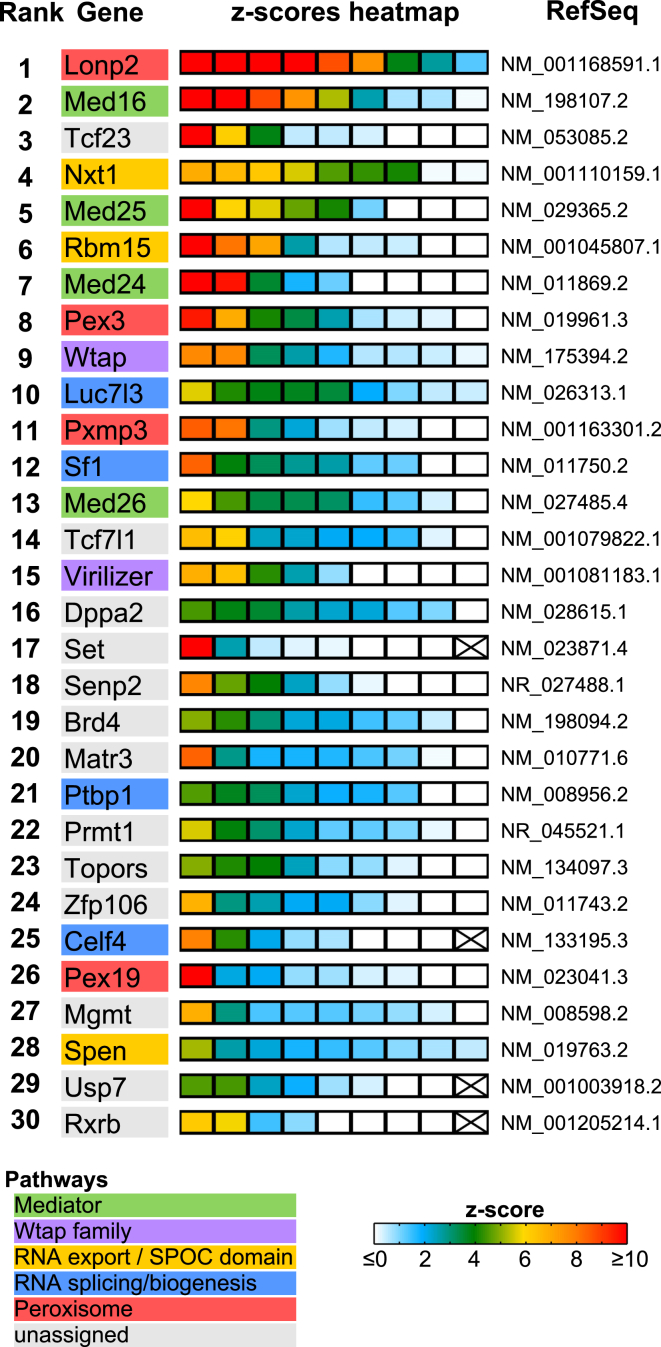
Top-Ranked Candidates from the Nucleome Screen The 30 top-ranked candidates from the nucleome screen are listed. The enrichment in the FACS-sorted cells (*Z*-score) of the corresponding hairpins is shown using a colored heatmap, where each square represents an independent hairpin. Crosses indicate excluded hairpins for which read number was below a set threshold. The candidates were ranked based on the *Z*-score for the hairpins targeting the same transcript. Factors linked to the same complexes/pathways are color coded. See also Figure S2.

A ranked list of the top targets from the nucleome (225) and ubiquitylome (34) libraries is shown in Tables S2 and S3. The highest ranking hits were all identified with multiple independent shRNAs, precluding off-target effects. There was a good correlation for individual shRNAs designed to ranked targets that are represented in both the nucleome and ubiquitylome, specifically Lonp2, Topors, Senp2, and Usp7 (Figure S2B), indicating reproducibility between independent experiments. Interestingly, we identified several factors associated with specific biochemically defined multisubunit complexes and/or specific pathways. Thus, among the top 30 ranked hits in the nucleome screen, we identified multiple subunits of Mediator complex, which is required for transactivation of RNA Polymerase II, as well as the RNA export factor Nxt1 and the SPOC domain protein Rbm15, which have been shown to interact with one another (Lindtner et al., 2006), together with Spen, an Rbm15 homolog. We also identified Wtap and Virilizer proteins, core subunits of the m6A RNA methyltransferase complex (Ping et al., 2014), different factors implicated in mRNA splicing/biogenesis, and several peroxisomal proteins that had an incidental nuclear GO annotation (Figure 2). Other components of the mRNA export complex (Nxt2#72 and Nxf1#103) and Mediator complex (Med10#208) were also identified (Table S2). Additional targets of potential interest included PRC1 Polycomb proteins (KDM2B#79, Scml2#168, and L3Mbtl3#178), other factors linked to heterochromatin (Prmt1#22 and Mbd3#51), nuclear matrix/chromosome structure proteins (Matr3#20 and Topors#23), and a pluripotency factor (Dppa2/4#16). Within the ubiquitylome screen, we identified the deubiquitylase Usp9x (#3), which had also been found in pilot screens performed using commercial whole-genome shRNA libraries (data not shown) and several subunits of the Cop9 signalosome (Table S3, #7, #9, #11 and #29).

**Figure 3:**
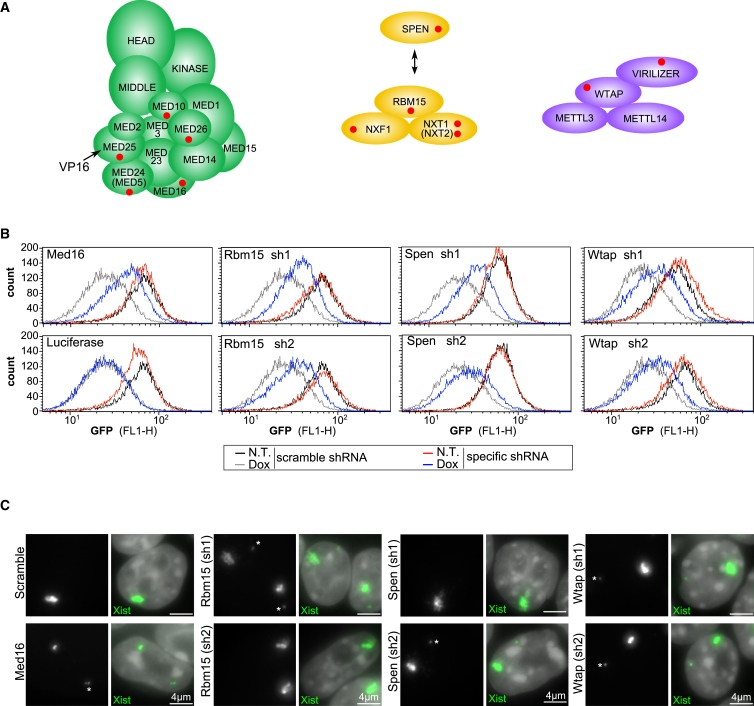
Validation of Selected High-Ranking Targets (A) Molecular complexes from the nucleome library: the Mediator complex (left); an RNA export complex composed of Nxf1, Nxt1, and the SPOC domain proteins Rbm15 and Spen (middle); the m6A methylation complex with Wtap and Virilizer (right). Subunits identified in the screen are labeled with a red dot. (B) GFP fluorescence of MG-3E reporter cell line with (gray/blue, 72 hr) or without (black/red) doxycycline, transduced with scramble (black/gray) or specific shRNA targeting Med16, Luciferase as a control, Rbm15, Wtap, and Spen. (C) RNA FISH illustrating that knockdown of Med16 impairs Xist domain formation, whereas Rbm15, Wtap, or Spen knockdown has no detectable effect. RNA FISH was performed 24 hr after doxycycline treatment. The asterisk indicates Tsix RNA foci that sometimes lie in the same focal plane. Scale bars are 4 μm. See also Figure S3.

Selected high-ranked targets were validated by FACS analysis of Mylc2b-GFP following transduction of induced and non-induced MG-3E cells with individual shRNAs and scrambled control (Figures 3A, 3B, and S3). A luciferase shRNA, together with a second scramble shRNA, were used as negative controls (Figures 3B and S3D). RT-PCR and/or antibody assays were used to determine the knockdown efficiency for the different shRNAs (Figures S4A–S4C). In several cases, for example, Med16, Rbm15, Spen, and Wtap, we observed enhanced GFP levels relative to the scrambled control following Xist RNA induction, demonstrating that knockdown of these factors indeed affects Xist-mediated repression of the GFP reporter (Figures 3B and S3A). In other cases, for example, the peroxisomal protein Lonp2 and Virilizer, we observed enhanced Mylc2b-GFP levels also in uninduced cultures (Figure S3B), indicating that the effect is, at least in part, independent of silencing by Xist RNA. This could occur, for example, through stabilization of GFP or GFP encoding mRNA. It should be noted that this does not rule out a role for these factors in Xist-mediated silencing. In the case of Nxt1, shRNA transduction severely reduced the viability of the cells, presumably because Nxt1 is required for the nuclear export of mRNAs. Because reduced viability could lead to counterselection that would bias the validation, we did no further analysis of this target. Finally, for Usp9x, ranked #3 in the ubiquitylome screen, we observed a relatively weak enhancement of Mylc2b GFP levels specifically in Xist-induced cells (Figure S3C).

The aforementioned FACS validation experiments highlighted Mediator, Rbm15, Spen, and Wtap as potentially having a role in Xist-mediated silencing. A priori, the knockdown of these positive hits could affect Xist transgene expression, Xist RNA localization or interfere with the downstream silencing cascade. We therefore analyzed Xist RNA expression using RNA FISH (Figure 3C) and RT-PCR (Figure S3E) following shRNA knockdown in induced MG-3E cells. Knockdown of the Mediator subunit Med16 resulted in a clear reduction in Xist domains and levels of Xist RNA. This is most likely due to dependence of doxycycline inducible transgene expression on co-activation by VP16, which in turn requires interaction with the Med25 subunit of the mediator complex tail region (Yang et al., 2004). Consistent with this suggestion, the majority of Mediator subunits identified in the screen are in the tail region and include Med25 (Figure 3A) (Malik and Roeder, 2010). We conclude that Mediator complex is important for the function of the MG-3E reporter system rather than for Xist-mediated silencing per se.

For Rbm15, Spen, and Wtap, Xist domains were apparently unaffected, although for Rbm15, levels of Xist RNA determined by RT-PCR were somewhat reduced (Figures 3C and S3E). Thus, these results indicate that Rbm15, Spen, and Wtap function primarily in Xist-mediated silencing and not in Xist RNA localization.

### Rbm15, Spen, and Wtap Are Required for Xist-Mediated Silencing

We went on to determine whether Rbm15, Spen, and Wtap have a role in silencing of other genes located in *cis* with the Xist transgene. Initially we used RT-PCR to assess expression levels of four chromosome 17 genes, SatB1, Enpp5, Crb3, and Fbxl17, previously shown to be downregulated following Xist RNA induction in MG-3E cells (Cerase et al., 2014). As shown in Figure S4D, knockdown of Rbm15, Spen, and Wtap and also Med16, used as a positive control, all resulted in elevated levels of the analyzed genes. While these effects varied from gene to gene, and between different shRNAs, control loci on other chromosomes, Dnmt1 and rtTA, were unaffected. Overall the results are consistent with knockdown of Rbm15, Spen, and Wtap affecting Xist-mediated silencing of the whole of chromosome 17.

**Figure 4:**
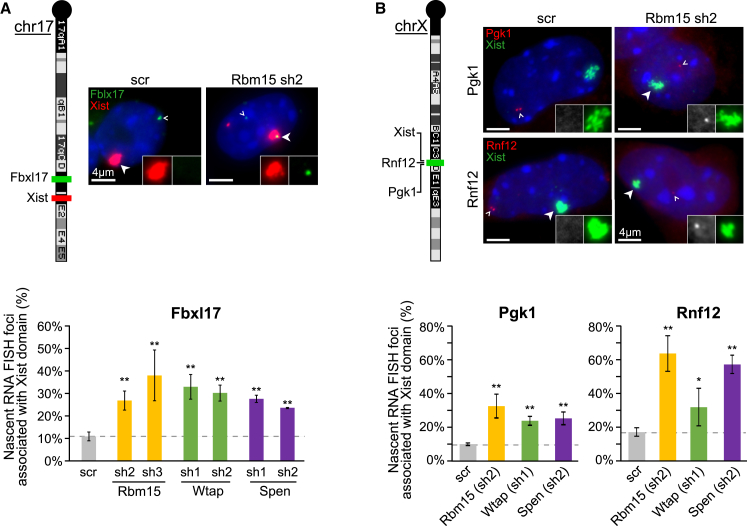
Rbm15, Wtap, and Spen Are Required for Xist-Mediated Transcriptional Silencing (A) Expression of Fbxl17 gene (green) was assessed within the Xist-coated chromosome (red) by nascent RNA FISH in 3E ESCs. (Top) The relative positions of Fbxl17 and Xist transgene are shown on the chromosome 17 ideogram. Images of individual cells after treatment with scramble or Rbm15 shRNAs. The insets correspond to a 1.75× magnification of the Xist cloud and show both red and green channels. (Bottom) Quantification (mean ± SD) of the proportion of cells with Fbxl17 allele expressed from the Xist-coated chromosome after treatment with different shRNAs. More than 150 cells from three independents experiments were scored. (B) Expression level of Pgk1 and Rnf12 genes (red) was assessed within the Xist-coated chromosome (green) by nascent RNA FISH in differentiated XT67E1 female ESCs. Relative positions of genes on chromosome X and example RNA FISH images with 1.75× magnification showing Xist in green and assessed genes in gray. Quantifications (mean ± SD) are shown below and were performed on more than 140 cells from three independents experiments. ^∗^p < 0.05, ^∗∗^p < 0.005 relative to scr shRNA (chi-square test). Scale bars are 4μm. The full arrowheads indicate the Xist cloud magnified in the insets; the open arrowheads indicate the expressed genes from the homologous chromosome. See also Figure S4.

To substantiate our findings using RT-PCR analysis, we assayed allelic silencing of Fbxl17 by RNA FISH, determining presence or absence of Fbxl17 nascent mRNA foci within or immediately adjacent to doxycycline-induced Xist RNA domains following transduction with Rbm15, Spen, Wtap, or scrambled shRNAs. The results, shown in Figure 4A, demonstrate that the frequency of Fbxl17 nascent RNA foci associated with Xist RNA domains is significantly and reproducibly elevated following knockdown of all three factors.

To determine the role of Rbm15, Spen, and Wtap in Xist-mediated gene silencing on the X chromosome, as opposed to in an Xist transgene model, we performed RNA FISH analysis of Xist RNA and nascent mRNA for two X-linked genes, Pgk1 and Rnf12, in differentiating XX embryonic stem cells (ESCs). As shown in Figure 4B, we observed a significant increase in the frequency of nascent mRNA foci associated with Xist domains for both genes following knockdown of Rbm15, Spen, and Wtap, as compared with scrambled shRNA control. Thus, together these experiments demonstrate a key role for Rbm15, Spen, and Wtap in gene silencing in *cis* mediated by Xist RNA.

**Figure 5:**
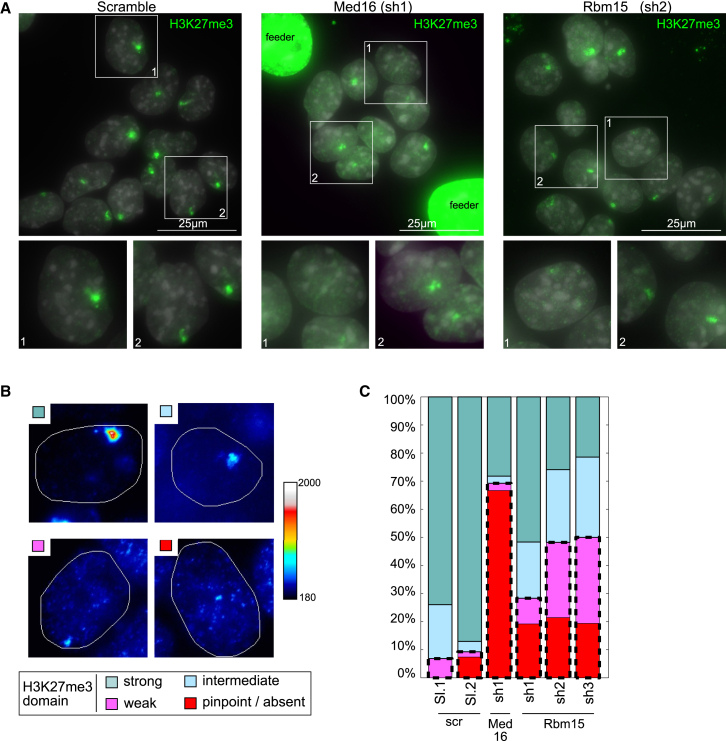
H3K27me3 Domains Are Altered by Rbm15 Knockdown (A) H3K27me3 domains in 3E cells treated with scrambled, Med16 or Rbm15 targeting shRNA. Xist was induced for 24 hr before the IF. The inserts (below) show magnifications. Scales bar are 25 μm. See also Figure S5A. (B) H3K27me3 domains were classified into four categories based on the color map (right). (C) Percentage of cells with strong/intermediate/weak/absent H3K27me3 domains as defined in (B); 70–120 cells were analyzed for each shRNA.

We went on to determine whether knockdown of Rbm15, Spen, and Wtap affect chromatin features of Xi, specifically the formation of H3K27me3 domains linked to Xist-mediated recruitment of the Polycomb complex PRC2. Initial analysis indicated Rbm15 knockdown leads to reduced intensity and size of H3K27me3 domains (Figure 5A). No obvious effect was seen with either Spen or Wtap knockdown (Figure S5A). To quantify the effect seen following Rbm15 knockdown, we developed a semiquantitative image analysis pipeline, defining four categories, strong, intermediate, weak, and absent K27me3 domains (Figure 5B). Knockdown of Med16 was again used as a control. As shown in Figure 5C, we observed a consistent reduction in the size of H3K27me3 domains using three independent Rbm15 hairpins. The level of reduction approached that seen following knockdown of the Med16 subunit that significantly reduces transgenic Xist RNA expression as reported above. We conclude that Rbm15 is important for efficient establishment of H3K27me3 domains on Xi.

### Super-resolution 3DSIM Reveals Rbm15, Spen, and Wtap Co-localize with Xist RNA

To further examine the function of Rbm15, Spen, and Wtap in Xist-mediated silencing, we used immunofluorescence to assess their nuclear localization in 3E mESCs expressing transgenic Xist RNA. The results, illustrated in Figure S5B, show that all three factors have a broad nuclear localization, with neither enrichment nor exclusion underlying Xist RNA domains, as assessed by co-staining for H3K27me3.

**Figure 6:**
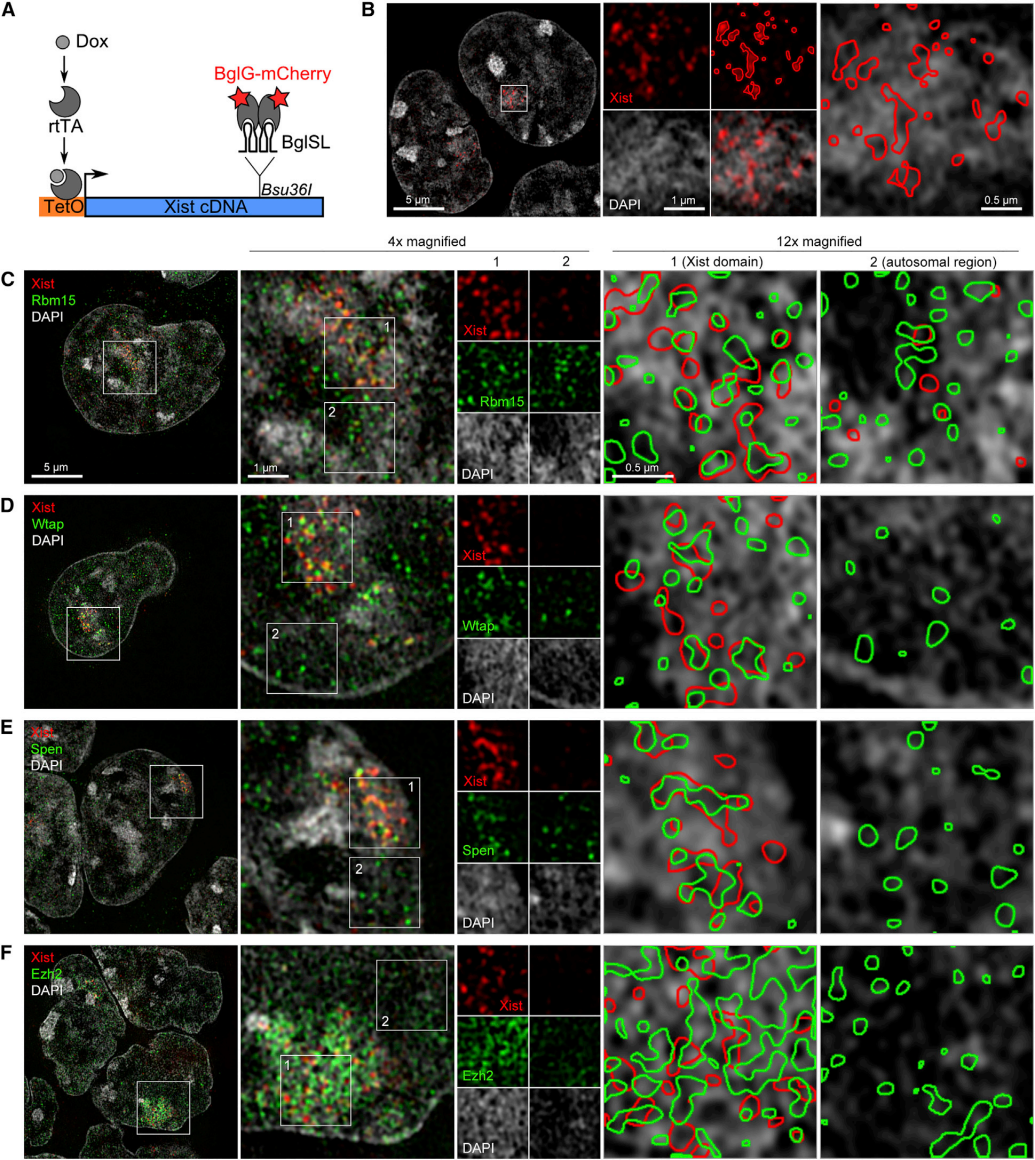
3DSIM Showing that Xist RNA, Rbm15, Wtap, and Spen Co-localize within Perichromatin Spaces (A) Xist tagging strategy using 18 copies of the Bgl stem-loop (BglSL) motif, which are recognized by the protein fusion BglG-mCherry. (B) Indirect detection of Xist RNA by immunofluorescence using an anti-mCherry antibody illustrates its localization within perichromatin spaces. (C–F) Double IF to analyze the distribution of Xist (red) and Rbm15 (C), Wtap (D), Spen (E), and the PRC2 component Ezh2 (F) (green). The 4×-magnified panels show the merged image. Insets 1 and 2, respectively, correspond to the Xist domain and the neighboring autosomal chromosome. The 12×-magnified panels illustrate the respective DAPI signal with outlined Xist (red) and analyzed protein (green) localization. See also Figure S6.

Analysis of Xi features and Xist RNA by super-resolution 3DSIM has shown that Xist RNA localizes to the perichromatin or nuclear matrix compartment, spatially separated from chromatin (Smeets et al., 2014). To analyze the localization patterns of Rbm15, Spen, and Wtap relative to Xist RNA, we made use of an ESC line, P4D7B1, in which inducible Xist RNA is tagged with Bgl stem loops that bind a BglG-mCherry fusion protein (Chen et al., 2009) (Figure 6A). This system bypasses the requirement to prepare samples using the relatively disruptive immunoFISH procedure. As shown in Figure 6B, BglG-mCherry signal accurately recapitulates Xist RNA localization within perichromatin spaces and clearly separated from chromatin.

We went on to determine the relative localization of BglG-mCherry and Rbm15 (Figure 6C), Wtap (Figure 6D), and Spen (Figure 6E) in P4D7B1 cells. As a control, we analyzed the PRC2 Polycomb protein Ezh2 (Figure 6F). The channel alignment for SR-3DSIM is shown in Figure S6. All three of the Xist silencing factors localized to perichromatin spaces, both within the Xist expression domains, and at other nuclear sites (Figures 6C–6E). Moreover, within the Xist expression domains, we observed extensive co-localization of the two signals (Figures 6C–6E, right). In contrast, BglG-mCherry and Ezh2 signal were on the whole spatially separated, consistent with our previous observations (Cerase et al., 2014). These findings demonstrate that Rbm15, Wtap, and Spen function within the same nuclear subcompartment in which Xist RNA is localized and therefore support that these factors could potentially interact with Xist RNA.

## Discussion

The genetic screen described here was designed to identify factors required for establishment of Xist-mediated silencing. Specifically, the reporter cell system functions within the critical developmental window of opportunity during which cells are Xist responsive. The use of an inducible Xist transgene system and of an unstable PEST-GFP reporter enabled us to focus the screen on the time period during which Xist-mediated silencing is initiated. The fact that we identified different subunits of defined complexes and also multiple factors linked to specific pathways indicates that the screen achieved a good degree of saturation. However, we cannot rule out that some factors evaded detection, for example, because of functional redundancy or incomplete coverage of the shRNA libraries.

Pooled shRNA knockdown offered specific advantages in the context of this screen. First, loss of function occurs across a broad dynamic range (because of cell-to-cell variation in knockdown efficiency and between different hairpins designed to the same gene), creating a virtual allelic series that facilitates identification of essential factors for which significant loss of function affects cell viability. Second, using a number of shRNA hairpins to each target provided a critical parameter for the ranking of positive hits.

Our screening procedure was developed after extensive optimization. Notably, we found that it was important to ensure that the number of cells transduced with a given shRNA was sufficient to reliably detect overrepresentation in the selected populations, particularly given that lentiviral transduction of ESCs is relatively inefficient. Linked to this, it was important to limit the coverage of the screen to factors in the nucleome and ubiquitylome in order to reduce false positive rates. Finally, inclusion of an HTS barcode in the shRNAs was essential to obviate the need to sequence across stem loops, which can introduce extreme bias.

Possible sources of false positives in the screen include knockdown of factors that affect the TetOn-inducible promoter system used to drive Xist expression in the reporter cell line and factors that influence the levels of the GFP-PEST reporter other than at the level of transcriptional silencing. Using validation assays that discriminate these possibilities, we found that Mediator knockdown strongly reduces TetOn promoter-driven Xist RNA expression, most likely linked to its requirement for VP16 mediated transactivation, and that knockdown of core peroxisome proteins increased levels of GFP-PEST protein, possibly linked to defects in protein turnover. Although we discarded these hits as false positives, the fact that we identified multiple factors in the same complex/pathway in both cases further demonstrates that the screen achieved a high degree of saturation.

### Rbm15, Spen, and Wtap Facilitate Xist-Mediated Silencing

The validation of top-ranked hits identified three factors, Rbm15, Spen, and Wtap, as playing a role in Xist-mediated silencing. Interestingly, there is evidence that these factors may interact with one another (Horiuchi et al., 2013; Malovannaya et al., 2011). Knockdown of all three factors suppressed Xist-mediated silencing in *cis* but had no obvious effect on the formation of Xist RNA domains. In the case of Rbm15, we also observed a deficiency in the formation of Xist-mediated H3K27me3 domains. SR-3DSIM analysis revealed co-localization with Xist RNA within the nuclear matrix/perichromatin compartment. Given that Rbm15 and Spen are RBPs, our SR-3DSIM observations are consistent with a direct role in binding Xist RNA. This proposal is further supported by two very recently published studies that identified Spen, Wtap, and Rbm15 among several proteins that crosslink to Xist RNA following either formaldehyde (Chu et al., 2015) or UV treatment (McHugh et al., 2015). Of note, both Rbm15 and Spen interactions were found using UV crosslinking (McHugh et al., 2015), supporting that they bind to Xist RNA directly. Both of these studies found that Spen is important for Xist-mediated silencing, in agreement with our observations. However, McHugh et al. (2015) did not detect silencing defects following knockdown of Rbm15, contrasting with our results. The reason for this discrepancy is unknown but could relate to the use of different shRNAs and/or silencing assays.

A comparative analysis of our results and those from the proteomic studies is provided in Table S4. In addition to the major candidates, the RBP Ptbp1, ranked 21 on our list, was identified as a direct Xist interactor in McHugh et al. (2015) and Chu et al. (2015), and the nuclear matrix protein Matr3, ranked 20 in our screen, was identified in Chu et al. (2015). The overlap between our analysis and the proteomic-based studies indicates that a good proportion of candidates identified but not yet validated in this study also function in the X-inactivation pathway. Of particular interest, several candidate factors highly ranked in our study were not identified in the proteomic screens. Notable examples are the RNA export factors Nxt1 and Nxf1/2, proposed Rbm15 interactors, and Virilizer, which interacts with Wtap. It should also be noted that several factors identified in the proteomic analyses were not present in our top-ranked list (Table S4). Of particular note, shRNAs for lamin B receptor (LBR), suggested as a key silencing factor by McHugh et al. (2015), and hnRNPK, which in Chu et al. (2015) was reported to contribute to silencing, showed no significant enrichment. Together, these examples highlight key similarities and differences that should be investigated in future work.

The mechanism of action of Rbm15, Spen, and Wtap in Xist-mediated silencing remains to be fully elucidated. Rbm15 and Spen are both Spen paralogue and orthologue C-terminal (SPOC) domain proteins, possibly indicating a common mode of action. The SPOC domain of Spen has been reported to interact with the co-repressor NCoR/SMRT, together with the histone deacetylase Hdac3 (Shi et al., 2001), and McHugh et al. (2015) found that knockdown of NCoR/SMRT and Hdac3 abrogates Xist-mediated silencing. In this regard, it is noteworthy that shRNAs for NCoR/SMRT and Hdac3 were not enriched in our analysis (Table S4). Similarly, the Rbm15 SPOC domain has been reported to interact with the histone H3K4 methyltransferase SET1B (Lee and Skalnik, 2012), but SET1B shRNAs are also not enriched in our screen. An alternative model for the role of Rbm15 in Xist-mediated silencing is that it functions in complex with the RNA export factors Nxt1/2 and Nxf1. This could potentially link to the fact that Xist RNA is retained in the nucleus, despite being spliced and polyadenylated (Brockdorff et al., 1992; Brown et al., 1992).

Wtap and Virilizer are subunits of the m6A methylation complex, important for the regulation of mRNA stability (Schwartz et al., 2014; Geula et al., 2015). Additionally, a recent study has shown that m6A methylation modifies RNA structure to facilitate binding of the hnRNPC protein required for RNA maturation (Liu et al., 2015). Based on this, we speculate that m6A methylation of Xist RNA may be important to enable binding of silencing factors such as Rbm15 and Spen. An important caveat, however, is that Mettl3, one of the catalytic subunits of the m6A methyltransferase complex, was not present on our ranked list, nor in the aforementioned proteomic screens. Thus, further studies are required to determine whether the link between Wtap and m6A methylation is in fact relevant in the context of Xist mediated silencing.

### Linking Xist Function to the Nuclear Matrix

Polycomb repressor proteins and several other factors implicated in Xi chromatin structure show a strong enrichment within Xist silencing domains relative to other regions of the nucleus. Our findings indicate that this is not the case for Rbm15, Spen, and Wtap, even though they clearly co-localize with Xist RNA. A possible explanation is that these factors are constitutive components of a machinery that localizes to perichromatin spaces, which functions, for example, in mRNA surveillance following release from RNA PolII complexes, prior to translocation to the nuclear pores. Xist RNA may have evolved to interact with this machinery in a manner that triggers a checkpoint that involves both RNA entrapment by nuclear matrix proteins such as hnRNPU/SAFA and signaling back to chromatin to shut down transcription. In relation to the latter, it is interesting to note that Spen is an unusually large protein (450 kDa) and as such could bridge the distance between Xist RNA and underlying chromatin, observed using SR-3DSIM.

In summary, we have used shRNA screening to identify novel factors that function in the establishment of Xist-mediated silencing. The screen achieved a high degree of saturation, indicating that the majority of key factors have been detected. Of particular interest, we validated that Rbm15, Wtap, and Spen are required for Xist-mediated silencing. Given that those three factors have very recently been identified as Xist RNA interacting proteins (Chu et al., 2015; McHugh et al., 2015), we believe that other targets identified in this study provide a rich resource for further investigation of the mechanism of Xist-mediated silencing.

## Experimental Procedures

For detailed experimental procedures, see Supplemental Experimental Procedures.

### Reporter Cell Line

The reporter cell line MG-3E was derived from XY 3E ESCs (Tang et al., 2010; Cerase et al., 2014) by replacing Mylc2b coding region located in *cis* with the Xist transgene with an unstable GFP (GFP:Pest; Corish and Tyler-Smith, 1999). Full details are provided in Supplemental Experimental Procedures.

### Cell Culture and shRNA Screen

ESCs were grown on feeders in DMEM supplemented with leukemia inhibitory factor (LIF)-conditioned medium. Before lentiviral infection, ESCs were trypsinized and pre-plated for 30 min to remove the feeders. For the pooled shRNA libraries 215 × 10^6^ (Nucleome sub-libraries) or 126 × 10^6^ (Ubiquitylome library) MG-3E cells were seeded in 14-cm dishes in ESC medium containing 8 μg/ml Polybrene. Lentiviruses were then added to the medium and cells grown for 24 hr at 37°C 5% CO_2_. The following day, ESCs were trypsinized and seeded on feeders. On day 3, Puromycin selection (2 μg/ml) was initiated and maintained until day 7. From day 4, Xist expression was induced by supplementing the medium with doxycycline (1.5 μg/ml). At day 7, cells were trypsinized and processed for FACS analysis.

Flow sorting (Beckman Coulter MoFlo XDP) was performed on 30–50 million MG-3E ESCs for each of three nucleome sublibraries and the ubiquitylome library. Cells with high GFP fluorescence (upper 5%) were collected. Candidates were identified and ranked based on the hairpin enrichment in the FACS-sorted compared with input populations (see Supplemental Experimental Procedures). A similar protocol was used for individual hairpin transductions, using smaller numbers of cells (the sequences of the shRNA used in validation experiments are listed in Table S5). For immunofluorescence (IF) following lentivirus infection, cells were trypsinized and seeded on slides on day 5, Xist was induced on day 6, and cells were fixed on day 7. For RNA FISH following lentivirus infection in 3E cell lines, cells were induced from day 4, trypsinized and seeded on slides on day 6, and fixed on day 7.

### Microscopy

Preparation of cells for RNA-FISH, IF, and 3DSIM was essentially as previously described (Nesterova et al., 2011; Smeets et al., 2014; Cerase et al., 2014). 3DSIM imaging was performed on a DeltaVision OMX V3 Blaze system (GE Healthcare). Modifications to protocols and all further details are provided in Supplemental Experimental Procedures.

## Author Contributions

A.C, T.B.N., and N.B. conceptualized the experiments. A.C. generated the reporter cell line, optimized and validated the initial screening strategy. B.M. performed and analyzed the genetic screen and validated the hits in the reporter cell line. T.B.N. performed the validations in differentiated ESCs. H.C. and L.S. performed the super-resolution imaging. A.G., G.P. and O.M. provided cell lines and reagents. N.B. wrote the manuscript. B.M., A.C., H.C., G.P., and T.B.N. reviewed and edited the manuscript. The manuscript has been approved by all authors. N.B. acquired and secured the funding.

## Supplemental Information

Document S1. Supplemental Experimental Procedures, Figures S1–S6, and Table S4

Table S1. Unprocessed Sequencing Data The excel file contains four sheets, one for the ubiquitylome and three for the three nucleome libraries. The hairpin count after high-throughput sequencing in the plasmid sample (used to transfect the 293T cells) in the Input population and the FACS-sorted population is indicated for each hairpin.

Table S2. List of Candidates from the Nucleome Screen The candidates are ranked based on the rescaled sum of the *Z* scores (RSZ). (Columns) Show the rank, Hugo name, the RefSeq ID of the targeted transcript, the RSZ, the total number of hairpin targeting this genes with more than 200 reads, the number of hairpins retained to calculate the RSZ, the number of positive hairpins (with a modified *Z* score above 2), the number of hairpins targeting this gene with a modified *Z* score above 3, between 2.5 and 3, and between 2 and 2.5, as well as the full name of the gene.

Table S3. List of Candidates from the Ubiquitylome Screen This is the same as Table S2, but for the ubiquitylome library.

Table S5. List of Primers Used for the RT-qPCR and shRNA Cloning into pLKO.1

Document S2. Article plus Supplemental Information
