# Supplementary material for: A Pooled shRNA Screen Identifies Rbm15, Spen, and Wtap as Factors Required for Xist RNA-Mediated Silencing
Source: Cell Rep. 2015 Jul 16;12(4):562–72. doi: 10.1016/j.celrep.2015.06.053 (PMC4534822; doi:10.1016/j.celrep.2015.06.053)
Supplement: Document S1. Supplemental Experimental Procedures, Figures S1–S6, and Table S4 [file mmc1.pdf]

Cell Reports

Supplemental Information

A Pooled shRNA Screen Identifies Rbm15, Spen, and Wtap as Factors Required for Xist RNA-Mediated Silencing

Benoit Moindrot, Andrea Cerase, Heather Coker, Osamu Masui, Anne Grijzenhout, Greta Pintacuda, Lothar Schermelleh, Tatyana B. Nesterova, and Neil Brockdorff

Figure S1

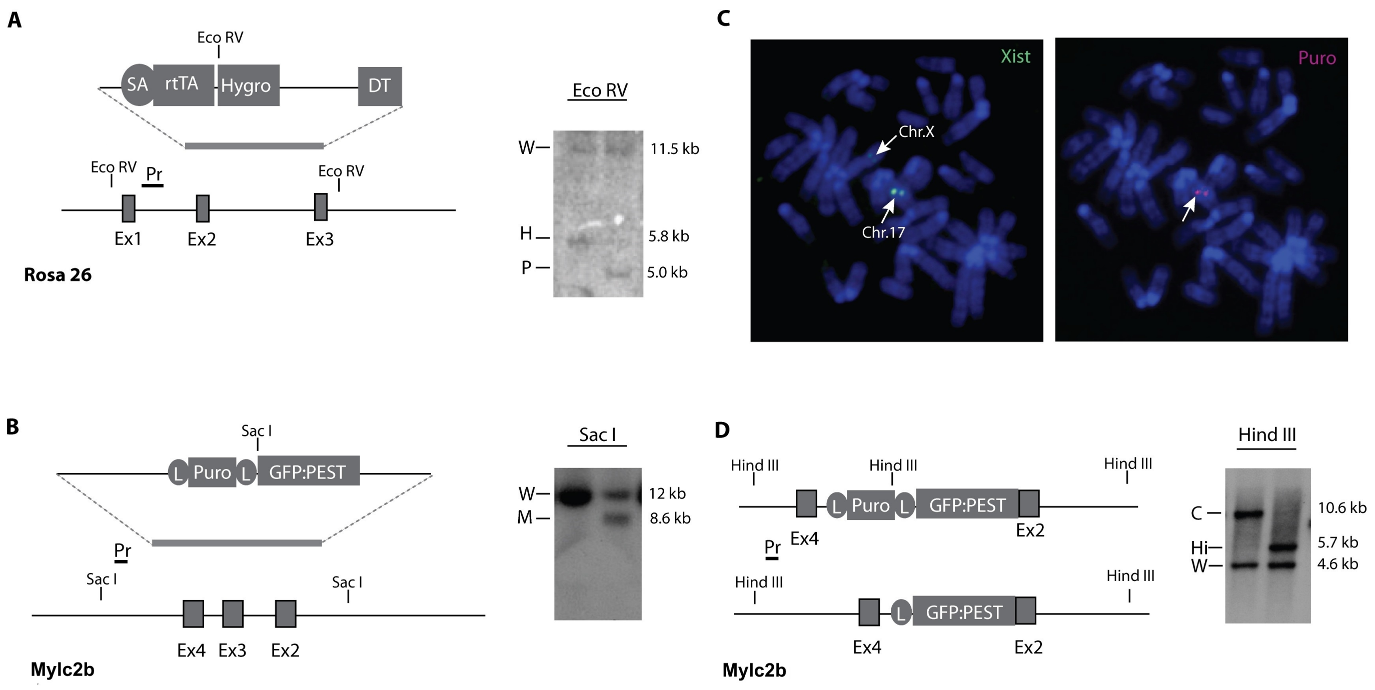

A
Eco RV
SA
rtTA
Hygro
DT
Eco RV
Pr
Eco RV
Ex1
Ex2
Ex3
Rosa 26
Eco RV
W
H
P
11.5 kb
5.8 kb
5.0 kb
C
Xist
Chr.X
Chr.17
Puro
B
Sac I
L
Puro
L
GFP:PEST
Pr
Sac I
Sac I
Ex4
Ex3
Ex2
Mylc2b
Sac I
W
M
12 kb
8.6 kb
D
Hind III
Hind III
Hind III
L
Puro
L
GFP:PEST
Ex4
Ex2
Pr
Hind III
Hind III
L
GFP:PEST
Ex4
Ex2
Mylc2b
Hind III
C
Hi
W
10.6 kb
5.7 kb
4.6 kb

Figure S2

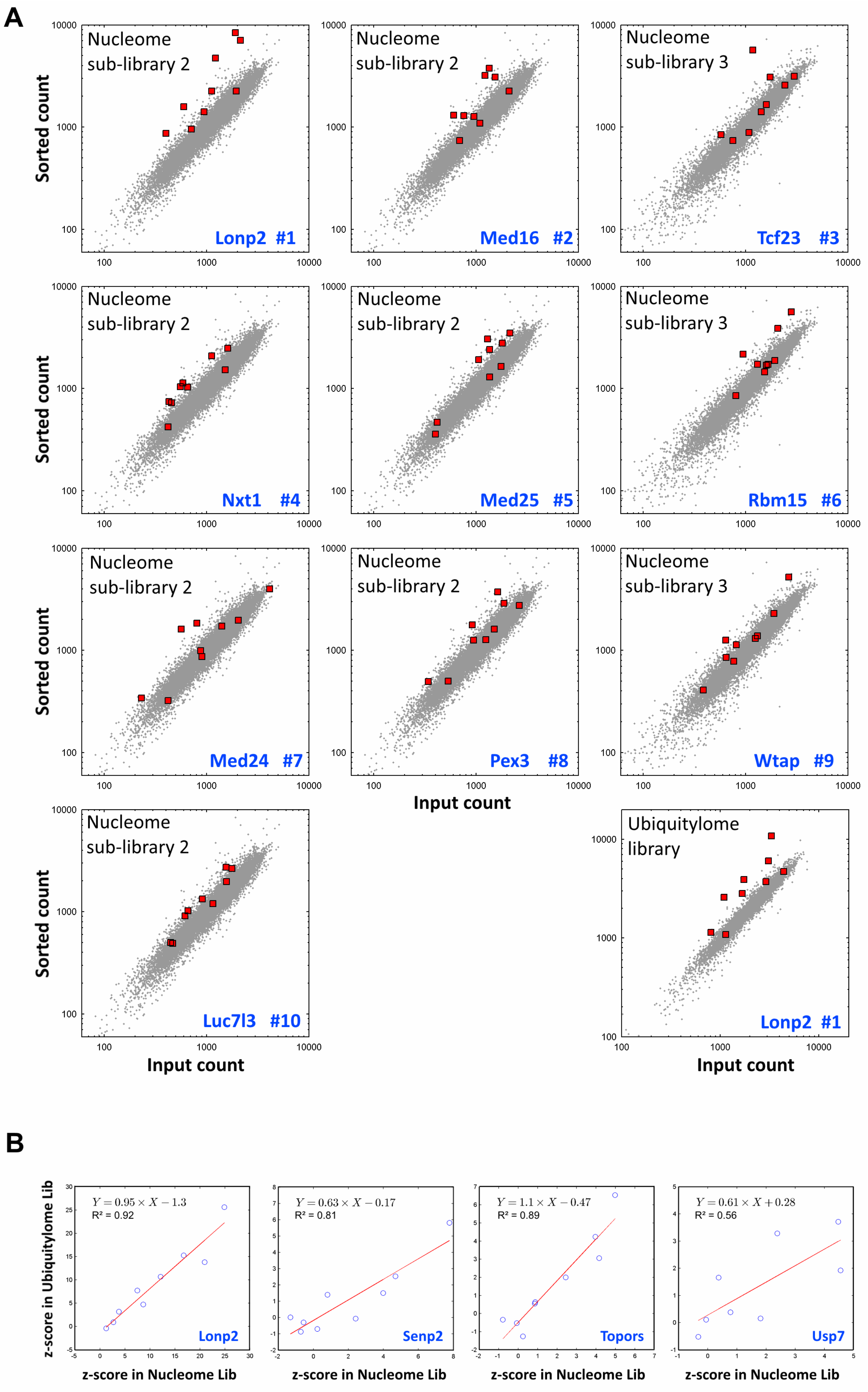
A
Sorted count
Input count
Nucleome sub-library 2
Lonp2 #1
Nucleome sub-library 2
Med16 #2
Nucleome sub-library 3
Tcf23 #3
Nucleome sub-library 2
Nxt1 #4
Nucleome sub-library 2
Med25 #5
Nucleome sub-library 3
Rbm15 #6
Nucleome sub-library 2
Med24 #7
Nucleome sub-library 2
Pex3 #8
Nucleome sub-library 3
Wtap #9
Nucleome sub-library 2
Luc7l3 #10
Ubiquitylome library
Lonp2 #1
B
z-score in Ubiquitylome Lib
z-score in Nucleome Lib
Y = 0.95 × X − 1.3
R² = 0.92
Lonp2
Y = 0.63 × X − 0.17
R² = 0.81
Senp2
Y = 1.1 × X − 0.47
R² = 0.89
Topors
Y = 0.61 × X + 0.28
R² = 0.56
Usp7

Figure S3

A

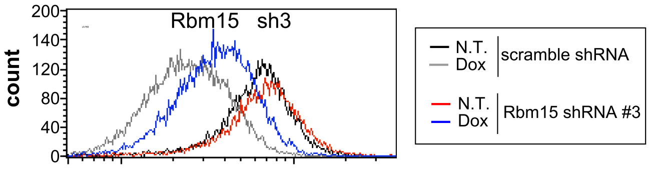

B

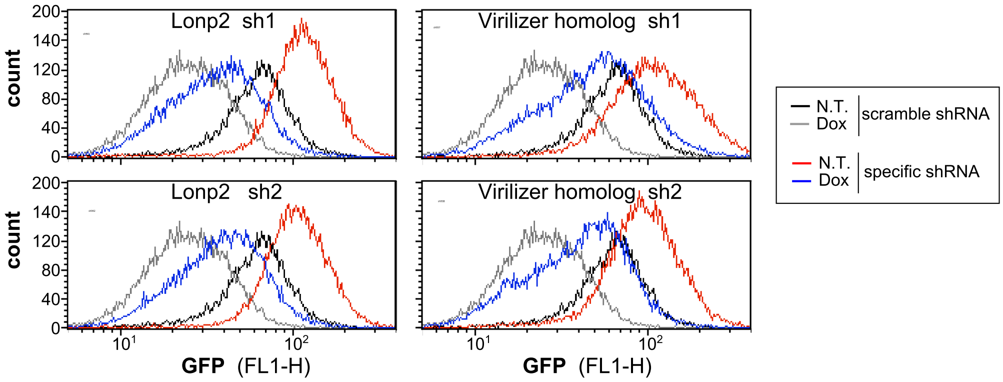

C

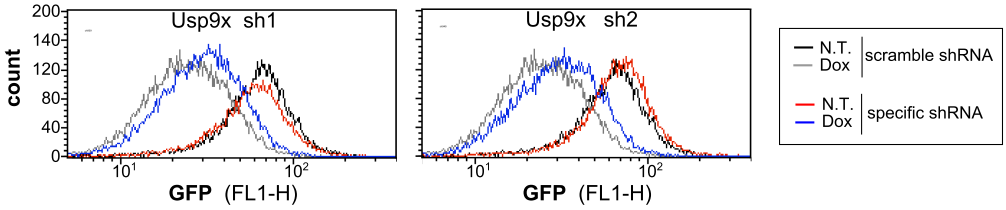

D

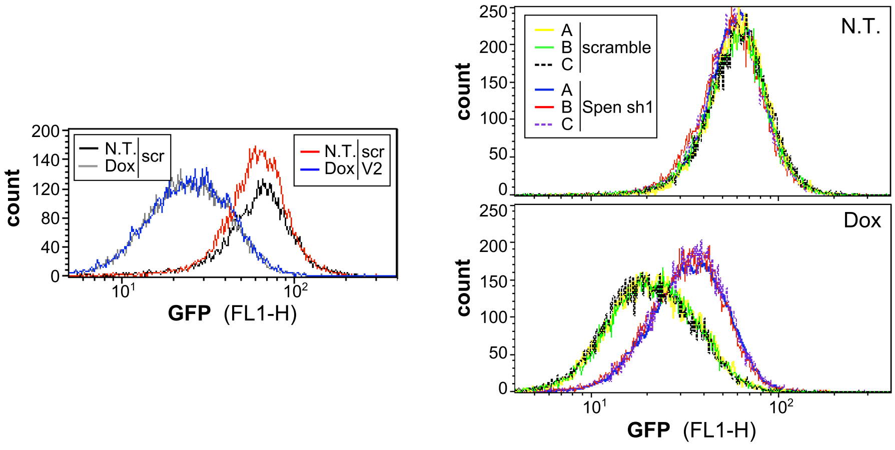

E

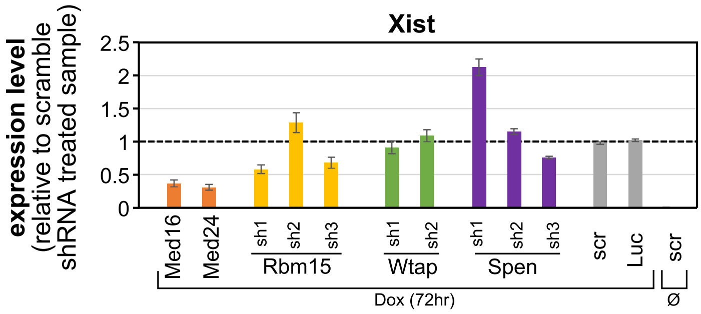

Figure S4

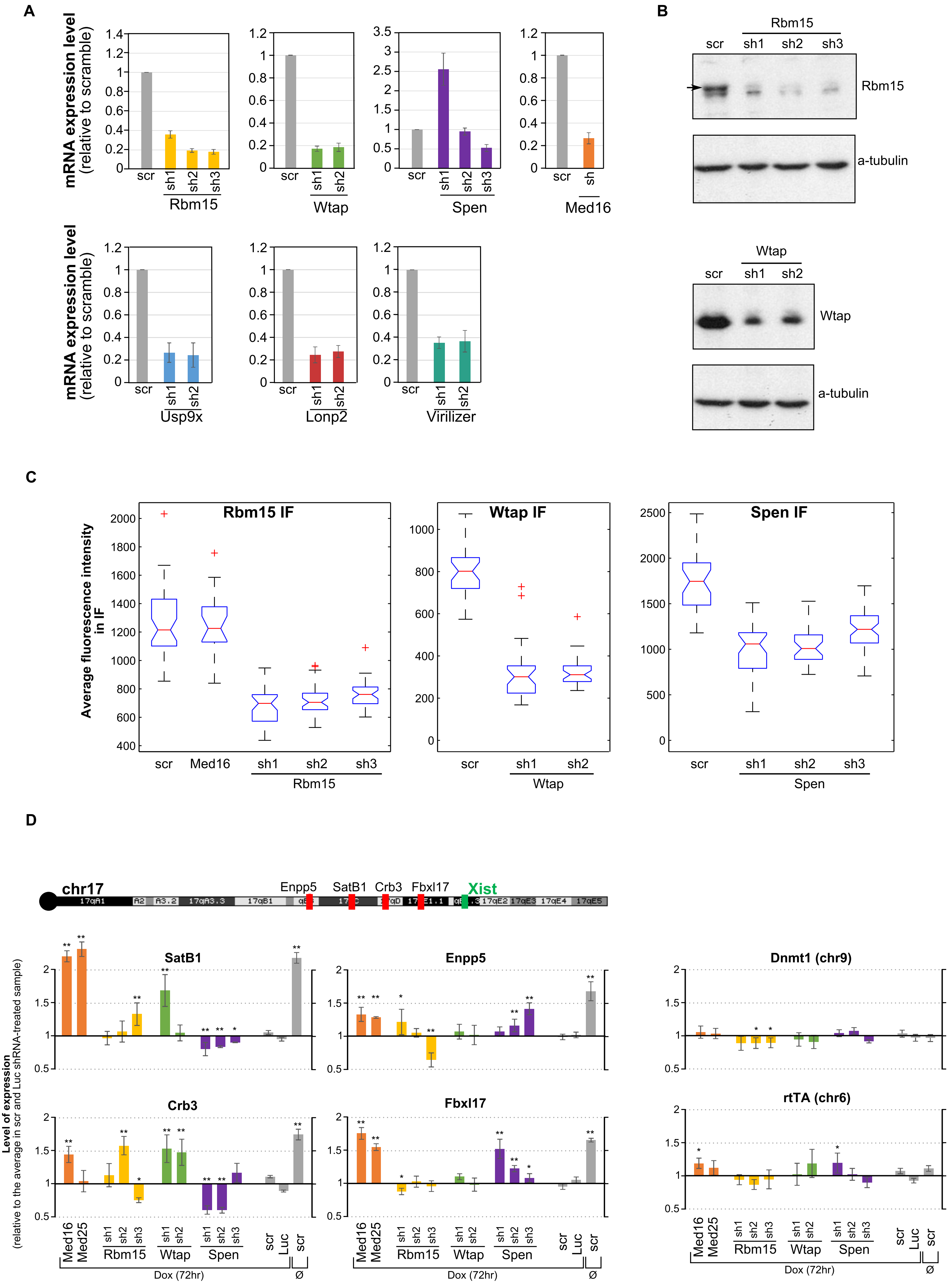

A
mRNA expression level
(relative to scramble)
scr
sh1
sh2
sh3
Rbm15
Wtap
Spen
sh
Med16
Usp9x
Lonp2
Virilizer
B
Rbm15
a-tubulin
Wtap
C
Rbm15 IF
Wtap IF
Spen IF
Average fluorescence intensity in IF
Med16
D
chr17
Enpp5
SatB1
Crb3
Fbxl17
Xist
SatB1
Enpp5
Dnmt1 (chr9)
Crb3
Fbxl17
rtTA (chr6)
Level of expression
(relative to the average in scr and Luc shRNA-treated sample)
Med25
Luc
Dox (72hr)
Ø

Figure S5

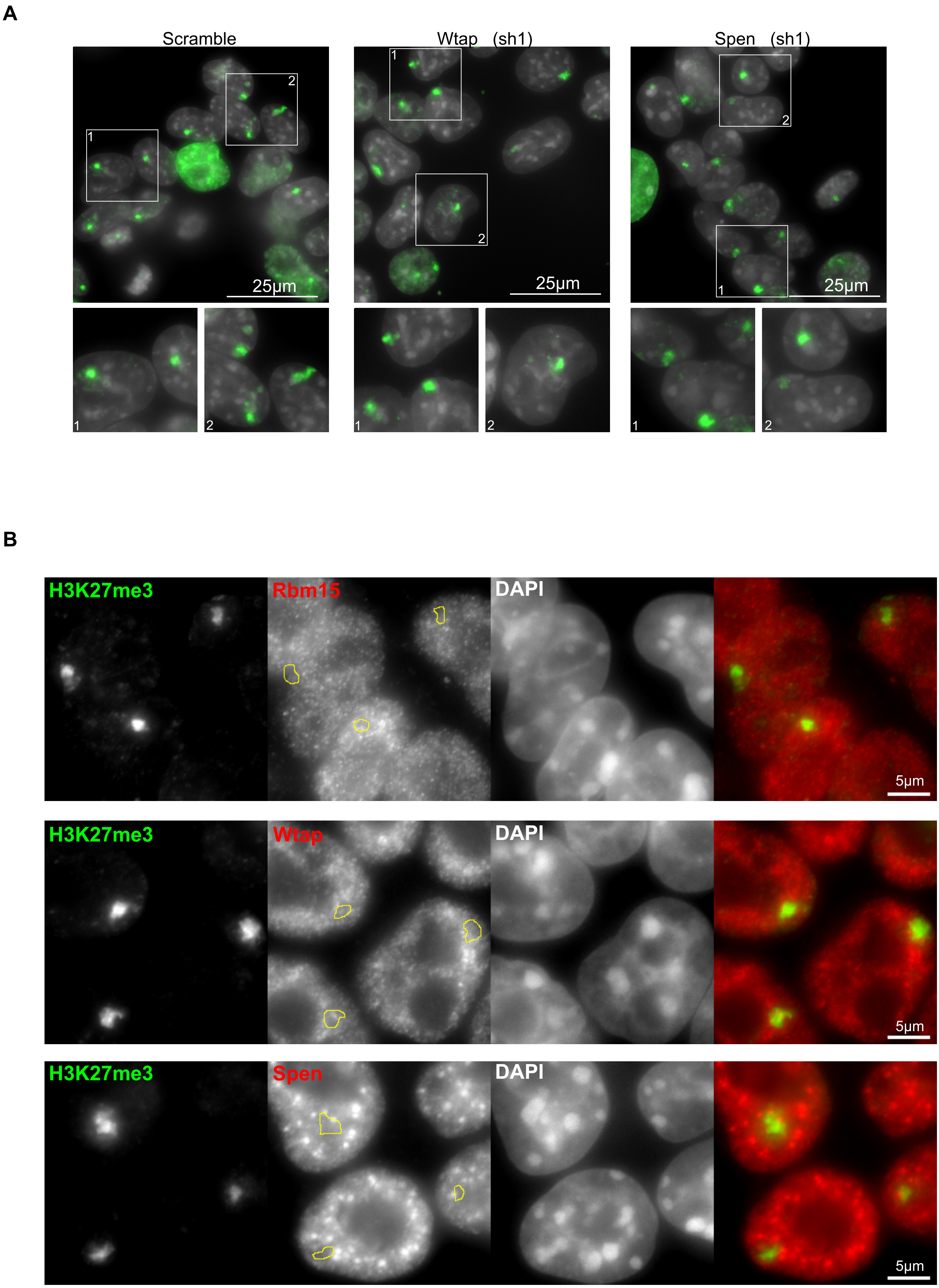
A
Scramble
Wtap (sh1)
Spen (sh1)
25µm
25µm
25µm
B
H3K27me3
Rbm15
DAPI
5µm
H3K27me3
Wtap
DAPI
5µm
H3K27me3
Spen
DAPI
5µm

Figure S6

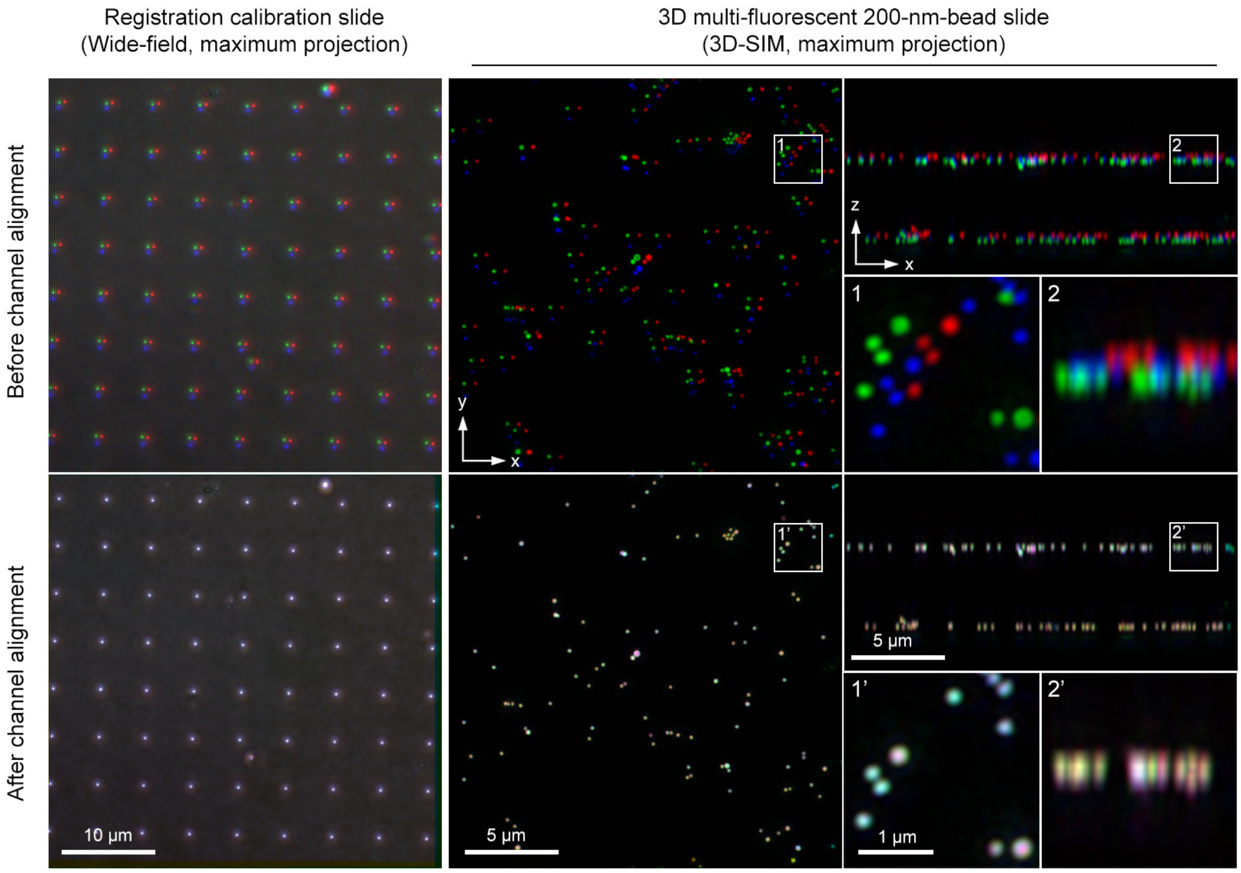

Registration calibration slide
(Wide-field, maximum projection)
3D multi-fluorescent 200-nm-bead slide
(3D-SIM, maximum projection)
Before channel alignment
After channel alignment
1
2
z
x
y
x
1
2
1'
2'
5 μm
1'
2'
10 μm
5 μm
1 μm

Supplementary Figure legends

Figure S1: Generation of the MG-3E reporter cell line, related to Figure 1.

A. Replacing puromycin cassette with a hygromycin cassette in Rosa26 locus. Schematic and Southern blot illustrate recombineering vector (top), WT allele (bottom). SA: splice acceptor, rtTA: reverse transactivator, Hygro: Hygromycin cassette, DT: Diphtheria Toxin cassette, Ex1-2-3: Exon 1-2-3, EcoRV: Restriction enzyme used for digestion, W: WT Rosa 26 allele, H: Hygromycin allele, P: Puromycin allele. Pr: probe. rtTA and Hygro have a polyA signal cassette at their 3' end. Molecular weight of digested fragments is shown.

B. Targeting GFP:PEST construct into Mylc2b locus. Schematic and Southern blot illustrate recombineering vector (top), WT allele (bottom). Ex2-3-4: Exon 2-3-4, L: LoxP site, Puro: Puromycin cassette, GFP:PEST: unstable GFP cassette. SacI: RE used for digestion, W: WT DNA, M: Mylc2b targeted allele. Molecular weight of digested fragments is shown. Pr: probe. Puro and GFP:PEST cassette have a polyA signal at their 3' end.

C. DNA FISH to verify that Xist and GFP:PEST are located *in cis* on chr17. Xist cDNA (green) and puro cassette (red) were used as probes. Chr. 17 and Chr. X are indicated.

D. Cre treatment of correctly targeted alleles to remove Puromycin cassette. Schematic and Southern blot illustrate targeted allele before (top) and after (bottom) Cre treatment. L: LoxP site, Puro: Puromycin cassette, GFP:PEST: unstable GFP, HindIII: RE used for digestion, W: WT DNA, Hi: HindIII + allele (before Cre treatment), C: Cre allele (after Puromycin cassette excised). Pr: probe. Molecular weight of digested fragments is shown. Schematics are not to scale.

Figure S2: Analysis of hairpin enrichment in GFP-high cells, related to Figure 2.

A. Distribution of shRNA scores for top 10 candidates from the nucleome screen. Each plot represents the hairpin count in unsorted and sorted MG-3E cells transduced with one of the three nucleome sub-libraries. The 9 hairpins targeting each candidate are shown in red. Some red dots are outliers in the upper left quadrant of the graph, indicating their enrichment in the FACS-sorted population. Lonp2 is ranked first in both the nucleome and ubiquitylome screens.

B. Screen reproducibility. Lonp2, Senp2, Topors, and Usp7 were identified in both the nucleome and ubiquitylome screens. The same hairpins were used in both libraries. We compared the enrichment (modified z-score) of each hairpin targeting one of those 4 genes in the two libraries. The strong correlation observed between these two independent libraries indicates a high degree of reproducibility.

Figure S3: Validation of top ranked targets, related to Figure 3.

A. GFP fluorescence of MG-3E reporter cell line with (grey/blue, 72hr) or without (black/red) doxycycline and transduced with scramble (black/grey) or Rbm15 sh3 shRNA.

B. Same than panel A but with shRNAs targeting Lonp2 or Virilizer Homolog. The knock-down of both genes increase the GFP brightness in the absence of doxycycline (Dox), indicating they affect GFP stability.

C. GFP fluorescence of MG-3E reporter cell line with (grey/blue, 72hr) or without (black/red) doxycycline (Dox) and transduced with scramble (black/grey) or specific shRNA targeting Usp9x. Usp9x was identified from the ubiquitylome screen (#3).

C. Reproducible scramble shRNA controls in MG-3E reporter cell line. Left: A second independent scrambled shRNA control does not affect the GFP fluorescence. Right: GFP-

fluorescence of the MG-3E reporter cell line transduced with scramble or Spen sh1 shRNA with (bottom) or without (top) doxycycline. The overlap between triplicates confirms good reproducibility of the approach.

D. Xist expression level following Med16, Med25, Rbm15, Wtap or Spen knockdown. Xist expression was induced for 72hr before RNA extraction (Dox 72hr). Expression levels are normalized to Actin, GAPDH and Idh2, and compared to the average expression levels for scrambled (scr) and Luciferase (Luc) shRNA-treated cells.

Figure S4: Knockdown efficiency and reduced silencing of chromosome 17 genes following knockdown of Rbm15, Wtap and Spen, related to Figure 4.

A. Relative expression level of Rbm15, Wtap, Spen, Med16, Usp9x, Lonp2 and Virilizer homologue in scrambled and specific shRNA-treated samples. Expression levels are normalized to Actin, GAPDH and Idh2. Bars represent mean ± SD in three experiments.

B. Western-blot for Rbm15 and Wtap in scrambled and target specific shRNA treated samples. Alpha Tubulin is used as a loading control.

C. Average intensity in immunofluorescence. Cells knocked-down for Rbm15, Wtap and Spen were seeded on slides and processed for IF using the corresponding antibodies. The average intensity was measured for more than 30 nuclei. The target specific shRNA treated cells showed reduced signal intensity relative to the scramble (scr) shRNA treated counterpart. Cells with Med16 knockdown, used as a control, that were stained using Rbm15 antibody show similar Rbm15 average fluorescence intensity than in the scramble shRNA treated cells.

D. RT-qPCR analysis of 4 genes located on chromosome 17 in Med16, Med25, Rbm15, Wtap and Spen knockdown 3E cells. Xist expression was induced for 72hr before RNA extraction (Dox 72hr). Expression levels are normalized to Actin, GAPDH and Idh2, and

compared to the average expression levels in scramble (scr) and Luciferase (Luc) shRNA treated cells. The expression of rtTA and Dnmt1, which are not on chromosome 17, are unaffected. The expression levels without doxycycline (ø) are also shown. The expected maximum variation is 2-fold as only one allele can be silenced by Xist. Bars represent mean ± SD in three experiments.

Figure S5: Rbm15, Wtap and Spen are not enriched within the inactive chromosome territory and their knockdown does not affect the H3K27me3 domains, related to Figures 5 and 6.

A. H3K27me3 domains are unaffected by knockdown of Wtap or Spen.

H3K27me3 domains in 3E cells treated with scramble, Wtap or Spen targeting shRNA. Xist was induced for 24hr before the IF. The inserts (below) show magnifications. Scale bars are 25µm.

B. Rbm15, Wtap and Spen are not enriched within the inactive chromosome territory.

3E cells were induced by doxycycline for 24hr and processed for immunofluorescence using Rbm15, Wtap and Spen antibodies. H3K27me3 staining was used to highlight the inactive chromosome territory.

Figure S6. Channel alignment for 3D-SIM co-localization analyses, related to Figure 6. Left column: conventional 3-channel acquisition of wide-field transmission illumination of special registration calibration slide (metal coated coverslip with microprocessed array of holes) before (top) and after (bottom) registration. Right column: Maximum intensity projection of 3D-SIM image stack of 200 nm diameter multi-fluorescent (TetraSpeck) beads attached to the coverslip (bottom), and slide (top) surface of the sample mounted

with 100% glycerol. Detailed lateral (ROIs 1 and 1’) and orthogonal (ROIs 2 and 2’) views confirm the accuracy of the alignment procedure in all spatial dimensions.

Table S4

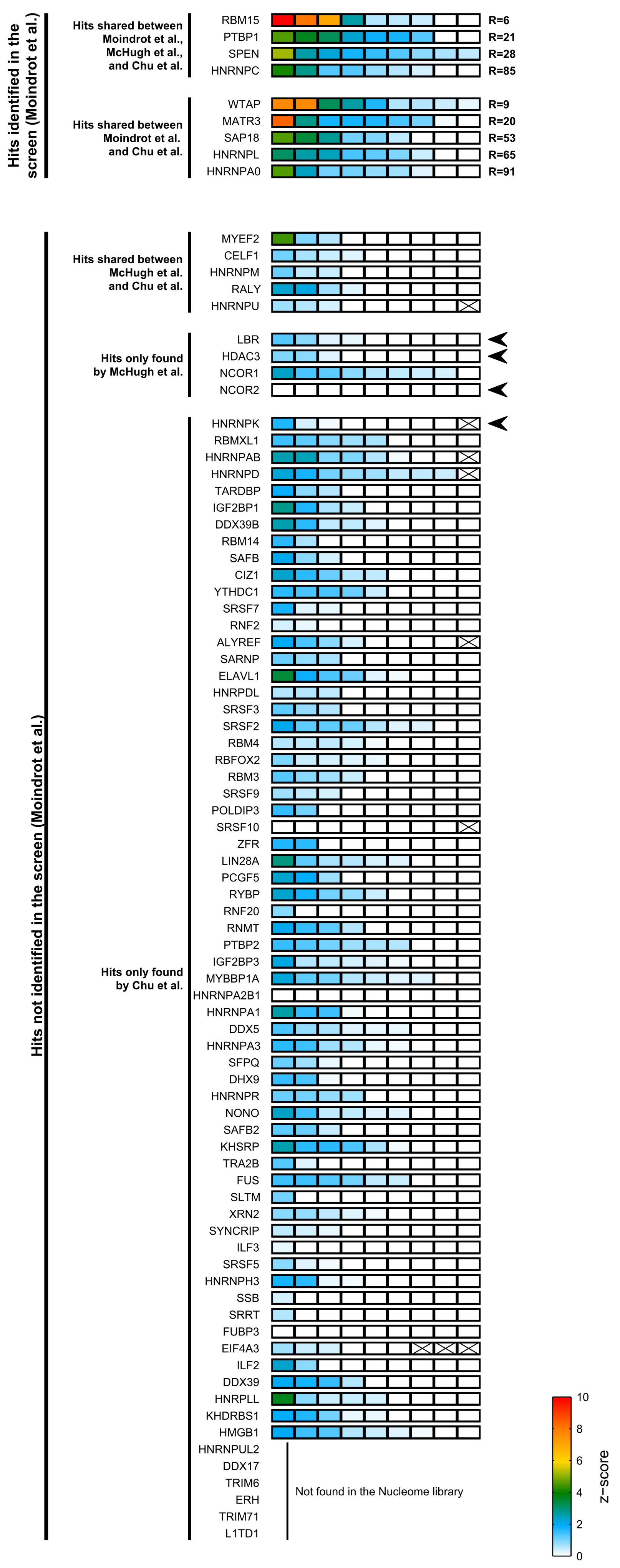
Hits identified in the screen (Moindrot et al.)
Hits shared between Moindrot et al., McHugh et al., and Chu et al.
RBM15
PTBP1
SPEN
HNRNPC
R=6
R=21
R=28
R=85
Hits shared between Moindrot et al. and Chu et al.
WTAP
MATR3
SAP18
HNRNPL
HNRNPA0
R=9
R=20
R=53
R=65
R=91
Hits not identified in the screen (Moindrot et al.)
Hits shared between McHugh et al. and Chu et al.
MYEF2
CELF1
HNRNPM
RALY
HNRNPU
Hits only found by McHugh et al.
LBR
HDAC3
NCOR1
NCOR2
Hits only found by Chu et al.
HNRNPK
RBMXL1
HNRNPAB
HNRNPD
TARDBP
IGF2BP1
DDX39B
RBM14
SAFB
CIZ1
YTHDC1
SRSF7
RNF2
ALYREF
SARNP
ELAVL1
HNRPDL
SRSF3
SRSF2
RBM4
RBFOX2
RBM3
SRSF9
POLDIP3
SRSF10
ZFR
LIN28A
PCGF5
RYBP
RNF20
RNMT
PTBP2
IGF2BP3
MYBBP1A
HNRNPA2B1
HNRNPA1
DDX5
HNRNPA3
SFPQ
DHX9
HNRNPR
NONO
SAFB2
KHSRP
TRA2B
FUS
SLTM
XRN2
SYNCRIP
ILF3
SRSF5
HNRNPH3
SSB
SRRT
FUBP3
EIF4A3
ILF2
DDX39
HNRPLL
KHDRBS1
HMGB1
HNRNPUL2
DDX17
TRIM6
ERH
TRIM71
L1TD1
Not found in the Nucleome library
10
8
6
4
2
0
z-score

Supplementary Table legends

Table S1: Unprocessed sequencing data

The excel file contains 4 sheets, one for the ubiquitylome, and 3 for the three nucleome libraries. The hairpin count after high-throughput sequencing in the plasmid sample (used to transfect the 293T cells), in the Input population and the FACS-sorted population are indicated for each hairpin.

Table S2: List of candidates from the nucleome screen.

The candidates are ranked based on the rescaled sum of the z-scores (RSZ). Columns: show the rank, Hugo name, the RefSeq ID of the targeted transcript, the RSZ, the total number of hairpin targeting this genes with more than 200 reads, the number of hairpins retained to calculate the RSZ, the number of positive hairpins (with a modified z-score above 2), the number of hairpins targeting this gene with a modified z-score above 3, between 2.5 and 3, and between 2 and 2.5, as well as the full name of the gene.

Table S3: List of candidates from the ubiquitylome screen.

Same as Table S2, but for the ubiquitylome library.

Table S4. Comparison of the hits from the genetic screen with recently published Xist RNA binding candidates identified in proteomics based studies (McHugh et al., 2015; Chu et al., 2015). The enrichment in the FACS-sorted cells (z-score) of every hairpin targeting a specific gene is represented using the same coloured heatmap as in Figure 2. Crosses indicate excluded hairpins for which read number was below a set threshold. The rank of the proteins identified in our genetic screen is indicated on the right side of the heatmaps. Arrowheads denote validated hits from the proteomics based studies.

Table S5: List of primers used for the RT-qPCR and the shRNA cloning into pLKO.1

Extended Experimental Procedures

Tissue culture

ES cells were grown on feeders (Puromycin resistant SNL) at 37°C 5% CO_2 in D-MEM (Life Technologies) supplemented with 10% fetal calf serum (Seralab), 2mM L-Glutamine (Life Technologies), 1X non-essential amino-acids (Life Technologies), 50µM 2-mercaptoethanol (Life Technologies), 1X Penicillin-Streptomycin (Life Technologies) and LIF-conditioned medium, made in-house, at a concentration equivalent to 1000 U/mL. To induce Xist expression, ES medium was supplemented with 1.5µg/mL doxycycline.
293T cells were grown at 37°C 5% CO_2 in D-MEM supplemented with 10% fetal calf serum, 2mM L-Glutamine, 1X non-essential amino-acids, 50µM 2-mercaptoethanol, 1X Penicillin-Streptomycin.

MG-3E Reporter cell line

A GFP reporter cell line was derived from the male 3E ES cell line (Tang et al., 2010) by DNA recombineering. A puromycin resistance cassette used to target the rtTA into Rosa26 locus in 3E cells was exchanged for a hygromycin resistance cassette. The coding region of Mylc2b, a gene previously shown to be sensitive to Xist silencing (Tang et al., 2010), was replaced by an unstable-GFP protein (GFP:PEST) (Corish and Tyler-Smith, 1999). Location of the GFP reporter *in cis* with the inducible Xist transgene was verified by DNA FISH using Xist cDNA and the puromycin resistance cassette as probes as described previously (Tang et al, 2010). Finally, the puromycin resistance cassette was excised from the Mylc2b locus by transient expression of CRE recombinase. MG-3E thus contains an inducible Xist transgene *in cis* with an unstable-GFP reporter, the expression of which is driven by the endogenous Mylc2b promoter, and is puromycin sensitive. Recombineering

steps were verified by Southern blot analysis using unique sequence probes as shown in Figure S1.

Lentivirus library

The nucleome library was designed to target all mouse genes with "Nucleus" gene ontology (GO:0005634) annotation. For the generation of a comprehensive mouse UPS list, GO searches were done for: "Ubiquitin Ligase Complex" GO-0000151, "Nuclear Ubiquitin Ligase Complex" GO-0000152, "Proteasome Complex" GO-0000502, "Ubiquitin-Specific protease activity" GO-00004843, "Proteasome Activator Complex" GO-00008537, "Protein Ubiquitination" GO-0016567, "Histone Ubiquitination" GO-0016574, "Protein Deubiquitination" GO-0016579, "Proteasome accessory complex" GO-0022624, "Nuclear Proteasome Complex" GO-0031595, "Proteosomal Ubiquitin-Dependent Protein Catabolic Process" GO-0043161, "C3HC4-type RING finger domain binding" GO-0055131, "RING-like Zinc Finger Domain Binding" GO-0071535, "Negative Regulation of Protein Deubiquitination". This GO-cumulative list was updated with the addition of "RBX, SKP, CULLIN, F-BOX, DDB, BTB, HECT, U-BOX, RING FINGER-containing protein" by GO searches. Manual annotated human E1, E2 and E3 Ligases, Ligase substrates and deubiquitinating enzyme list were added from the hUbiquitome database (http://202.38.126.151/hmdd/hubi/, (Du et al., 2011)). A comprehensive unique-only UPS gene-symbol list was created. This list was then converted in a mouse MGI/Gene symbol list using the David tool online (http://david.abcc.ncifcrf.gov/conversion.jsp). The final list contains 1004 "bona fide" Ubiquitin proteasome Associated factors. shRNA design and production of pooled shRNA libraries were performed by Cellecta Inc.

Lentivirus production

Selected hairpins were cloned in pLKO.1 using the oligonucleotides listed in Table S5.

For individual hairpin lentivirus production, 293T cells were transfected in a 10cm-dish using 40µL of Lipofectamine 2000 by 4.5µg psPAX2 + 1.5µg pMD2.G + 6µg pLKO.1 containing the appropriate hairpin. The medium was replaced the following day, and lentivirus was collected 44hr and 52hr after transfection. Lentivirus containing medium was then filtered through a 0.45µm PVDF filter, aliquoted and snap frozen. For the pooled shRNA libraries, the same protocol was applied except that 35 (for each nucleome sub-library) or 20 (ubiquitylome library) 10-cm dishes of 293T were processed at the same time. Lentivirus containing medium was filtered through a 0.45µm PVDF filter and concentrated using Lenti-X Concentrator (CloneTech), following manufacturer's instructions.

Flow cytometry

Cells were washed with PBS, resuspended in PBS containing 3.5% fetal calf serum and 1.5µg/mL doxycycline. 30-50 million ES cells were analyzed by FACS (Beckman Coulter MoFlo XDP) for the ubiquitylome library and each of the 3 nucleome sub-libraries, and the 5% most fluorescent cells were sorted. 30,000 ES cells were analyzed (BD FACSCalibur), after transduction of individual hairpin.

DNA extraction for the shRNA barcode amplification and quantification

Input and Sorted cells (5% most GFP fluorescent cells) were processed as follows.

ES cells were lysed in 10mM NaCl, 10mM Tris pH7.5, 10mM EDTA, 0.5% Sarcosyl, 20µg/mL RNAseA. DNA was sheared by passing the lysate through a 23G needle. The lysate was then incubated overnight at 55°C in presence of 200µg/mL Proteinase K, phenol-chloroform extracted, and ethanol precipitated. DNA was then resuspended in 10mM Tris pH7.5 and quantified by Nanodrop. Barcode amplification, HTS, and barcode count was carried out by Cellecta Inc.

Candidate identification and ranking

For every hairpin we calculated its enrichment (Ei) in the Sorted 5% most fluorescent cells by dividing the hairpin count in the sorted sample by the hairpin count in the Input. We then calculated the Modified Z-score of each hairpin (ModZi)

$$ModZ_i = \frac{0.6745 \times (E_i - \widetilde{E})}{median\{|E_i - \widetilde{E}|\})}$$

where $\widetilde{E}$ is the sample median enrichment for the hairpin of a given (sub)-library.

We then combined the Modified Z-score of the 9 hairpins targeting the same genes and kept only those for which both the count in the input and sorted samples were > 200 reads. To generate the list of candidates, we limited for genes having at least one hairpin with a modified Z-score above 3 and at least one other hairpin with a modified Z-score above 2. We ranked this list of candidates according to the rescaled sum of the z-scores (RSZ) calculated on the 7 (out of 9) largest modified z-scores to account for potential off-target hairpins.

Immunofluorescence

Slides were washed with PBS, fixed with 2% formaldehyde in PBS for 15 min, permeablised with 0.4% Triton X-100 in PBS for 5 min and then blocked with 0.2% fish skin gelatin (Sigma) in PBS (30-60min). Primary antibodies were used as follows: H3K27me3 (1:500 ActiveMotif #61017), Rbm15 (1:100 ProteinTech 10587-1-AP), Wtap (1:200 ProteinTech 10200-1-AP), Spen (1:250 Abcam ab72266). Secondary Antibodies were Alexa-fluor conjugated (1:400 Life Technologies). Slides were mounted in Vectashield with DAPI and visualised using a 63x oil immersion objective with the Zeiss Axio Observer Z1 microscope. Images were analysed with ImageJ. H3K27me3 domains were segmented using 3D spot segmentation plugin for ImageJ (Ollion et al., 2013).

Strong and weak domains are automatically detected by the plugin, with strong domain having a mean intensity > 900. Weak domains were detected by eye only.

RNA FISH

1µg Xist cDNA was labelled with green-dUTP (Abbott Molecular) using a nick translation kit (Abbott Molecular) in a total volume of 50µL. For every 22x22mm coverslip, 2.5µL of labelled probe were ethanol precipitated with 1µg of Salmon Sperm DNA (Life Technologies), and resuspended in 12µL of 50% formamide, 2X SSC, 10% dextran sulfate, 1mg/mL BSA. Coverslips were washed with PBS, fixed with 2.6% formaldehyde in PBS for 10min at room temperature and permeabilized with 0.42% Triton X-100 in PBS for 5min on ice. Coverslips were then washed with PBS, dehydrated with 70%-80%-95%-100% ethanol, and hybridized overnight at 37°C in humid chamber. The following day, the coverslips were washed 3 times with 50% formamide, 2X SSC at 42°C, and 3 times with 2X SSC at 42°C.Coverslips were mounted in Vectashield with DAPI and visualised using a 63x oil immersion objective with the Zeiss Axio Observer Z1 microscope.

For Fbxl17 nascent RNA-Fish, 1µg RP24-324P16 BAC was labelled using Red-dUTP (Abbott Molecular) using a nick translation kit (Abbott Molecular) in a total volume of 50µL. For every 22x22mm coverslip, 1.5µL of labelled Xist and 2.5µL of labelled BAC were ethanol precipitated with 1µg of Salmon Sperm DNA and 3µg of mouse Cot-1 (Life Technologies), denaturated for 5min at 75°C in 50% formamide, 2X SSC, 10% dextran sulfate, 1mg/mL BSA and competed for 30min at 37°C.

Rnf12 and Pgk1 nascent RNA-FISH was performed in differentiated female ES cells. Feederless XT67E1 XX ES cell line (Penny et al., 1996) was grown on gelatinized plates in D-MEM medium supplemented with LIF. Individual hairpin knockdowns were performed as described above, and stable puromycin-resistant populations of XT67E1 cells were selected for 5-6 days. To induce differentiation, XT67E1 cells were trypsinised and seeded

at low density ($1.5x10^4$ cells/cm^2) on non-gelatinized plates in D-MEM medium without LIF. After 5 days of differentiation cells were trypsinised, plated on coverslips and left in differentiation conditions overnight. The following day (day 6 of differentiation) coverslips were rinsed in PBS and fixed in 3.7% formaldehyde for 10 min at room temperature. Cells were permeabilised with 0.5% Triton X100 in CSK buffer for 5 min on ice (Clemson et al., 1996), rinsed in PBS and stored in 70% ethanol at +4˚C until use. Rnf12 and Pgk1 nascent transcripts were visualized with 48 (Rnf12) or 96 (Pgk1) Quasar® 570-labelled oligonucleotides designed against uniqueintronic sequences using the online Stellaris® Probe Designer tool from BioSearch Technologies (USA). For every coverslip, 1µL of Spectrum-Green labeled Xist probe was resuspended in BioSearch Technologies hybridization buffer, denatured for 5min at 75°C and kept on ice until use. The Quasar® 570-labelled oligonucleotide probes were mixed with Xist probe just before the hybridization (250nM final concentration). Hybridization and washes were done following the manufacturer's instructions. Coverslips were visualised using a 63x oil immersion objective with the Zeiss Axio Observer Z1 microscope.

Structured illumination microscopy

Cells for SR-3DSIM were seeded onto No. 1.5H (170 µm ± 5 µm) coverslips (Marienfield) and BglSL-Xist expression was induced with doxycycline for 24hrs. Cells were fixed with 2% formaldehyde for 10 min, permeabilised in 0.5% Triton X-100 PBS for 10 minutes. Initial blocking (30 min) and all further antibody incubations were carried out in 3% BSA, 5% normal goat serum (Sigma), 5% normal donkey serum (Santa Cruz) and 0.5% fish gelatine in PBS with 0.05% tween-20. Coverslips were incubated with primary antibody for 1 h in a humidified chamber (rabbit anti-Rbm15, 1/100 Proteintech 10587-1-AP, Rabbit anti-Spen, 1/1000 Atlas antibodies HPA015825, Rabbit anti-Wtap, 1/500 Proteintech 10200-1-AP, Rabbit anti-Ezh2, 1/200 Cell Signalling, and sheep anti-mCherry (kind gift of

Francis Barr) 1/500. After extensive washing in PBS-T, coverslips were incubated with Alexa Fluor goat anti-rabbit 488 and Alexa Fluor donkey anti-sheep 568, 1/1000 for 30min. Cells were again extensively washed, then post fixed with 2% formaldehyde before incubation with 2µg/ml DAPI for 10 minutes.

Super-resolution 3D-SIM was performed on a DeltaVision OMX V3 Blaze system (GE Healthcare) equipped with a 60x/1.42 NA PlanApo oil immersion objective (Olympus), sCMOS cameras (PCO), and 405, 488 and 593nm lasers. 3D-SIM image stacks were acquired with a z-distance of 125nm and with 15 raw images per plane (5 phases, 3 angles). The raw data was computationally reconstructed with SoftWoRx 6.1 (GE Healthcare) using channel-specifically measured optical transfer functions (OTFs) and Wiener filter settings 0.0020 (green/red channel) and 0.0040 (blue channel), respectively, to generate 3D stacks with ~120nm lateral and ~300nm axial resolution. Spherical aberration was minimized by matching the refractive indices (RI) of the immersion oil for sample acquisition (RI 1.514) and for generation OTFs (RI 1.512 for the blue and green, and 1.514 for the red OTFs) generated from ~100nm diameter blue (FluoroMax; Thermo Scientific), green and red (FluoSpheres; Life Technologies) beads, respectively. Lateral colour channel alignment was performed using a special image registration slide and algorithm provided by GE Healthcare. Correct 3D alignment was confirmed and refined in z by a custom test sample with two layers of 0.2µm diameter TetraSpeck beads (Life Technologies) (Figure S6). The full-scale 32-bit reconstructed data was thresholded for each channel to the stack modal grey value (representing the centre of the background intensity level) and converted to 16-bit composite tif-stacks using an in-house script in ImageJ (http://rsbweb.nih.gov/ij) before further processing.

Gene expression analysis

RNA was extracted with TRIzol® Reagent (Life Technologie) following manufacturer's instructions. The DNAse treatment was with TURBO DNA-free™ Kit (Life Technologies). The Reverse Transcription reaction was carried on 2µg RNA using SuperScript® III Reverse Transcriptase (Life Technologies). Quantitative PCR assays were carried out using the Rotor-Gene Q (Qiagen) and the iQ SYBR GREEN Supermix (BioRad) using the primers listed in Table S5.

Xist RNA tagging with Bgl-mCherry

A tandem array of 18 repeats of Bgl stem loop (BglSL) motif was inserted at a unique Bsu36I restriction site in the inducible Xist cDNA construct. The modified construct was then transfected into ES cells together with a plasmid encoding the fusion between the N-terminal RNA recognition motif of BglG and mCherry protein (Declerck et al., 2002; Chen et al., 2009). Stable cell lines were screened to identify lines with tagged Xist expression after 24hr of doxycycline treatment.

Supplemental references

Chen, J., Nikolaitchik, O., Singh, J., Wright, A., Bencsics, C. E., Coffin, J. M., Ni, N., Lockett, S., Pathak, V. K. and Hu, W.-S. (2009). High efficiency of HIV-1 genomic RNA packaging and heterozygote formation revealed by single virion analysis. Proc Natl Acad Sci U S A *106*, 13535–13540.

Chu, C., Zhang, Q. C., da Rocha, S. T., Flynn, R. A., Bharadwaj, M., Calabrese, J. M., Magnuson, T., Heard, E. and Chang, H. Y. (2015). Systematic discovery of Xist RNA binding proteins. Cell *161*, 404–416.

Clemson, C. M., McNeil, J. A., Willard, H. F. and Lawrence, J. B. (1996). XIST RNA paints the inactive X chromosome at interphase: evidence for a novel RNA involved in nuclear/chromosome structure. J Cell Biol *132*, 259–275.

Corish, P. and Tyler-Smith, C. (1999). Attenuation of green fluorescent protein half-life in mammalian cells. Protein Eng *12*, 1035–1040.

Declerck, N., Minh, N. L., Yang, Y., Bloch, V., Kochoyan, M. and Aymerich, S. (2002). RNA recognition by transcriptional antiterminators of the BglG/SacY family: mapping of SacY RNA binding site. J Mol Biol *319*, 1035–1048.

Du, Y., Xu, N., Lu, M. and Li, T. (2011). hUbiquitome: a database of experimentally verified ubiquitination cascades in humans. Database (Oxford) *2011*, bar055.

McHugh, C. A., Chen, C.-K., Chow, A., Surka, C. F., Tran, C., McDonel, P., Pandya-Jones, A., Blanco, M., Burghard, C., Moradian, A., Sweredoski, M. J., Shishkin, A. A., Su, J., Lander, E. S., Hess, S., Plath, K. and Guttman, M. (2015). The Xist lncRNA interacts directly with SHARP to silence transcription through HDAC3. Nature *521*, 232–236.

Ollion, J., Cochennec, J., Loll, F., Escudé, C. and Boudier, T. (2013). TANGO: a generic tool for high-throughput 3D image analysis for studying nuclear organization. Bioinformatics *29*, 1840–1841.

Penny, G. D., Kay, G. F., Sheardown, S. A., Rastan, S. and Brockdorff, N. (1996). Requirement for Xist in X chromosome inactivation. Nature *379*, 131–137.

Tang, Y. A., Huntley, D., Montana, G., Cerase, A., Nesterova, T. B. and Brockdorff, N. (2010). Efficiency of Xist-mediated silencing on autosomes is linked to chromosomal domain organisation. Epigenetics Chromatin *3*, 10.
