# Supplementary material for: A Pooled shRNA Screen Identifies Rbm15, Spen, and Wtap as Factors Required for Xist RNA-Mediated Silencing
Source: Cell Rep. 2015 Jul 16;12(4):562–72. doi: 10.1016/j.celrep.2015.06.053 (PMC4534822; doi:10.1016/j.celrep.2015.06.053)
Supplement: Document S2. Article plus Supplemental Information [file mmc6.pdf]

Cell Reports

Article

A Pooled shRNA Screen Identifies Rbm15, Spen, and Wtap as Factors Required for Xist RNA-Mediated Silencing

Graphical Abstract

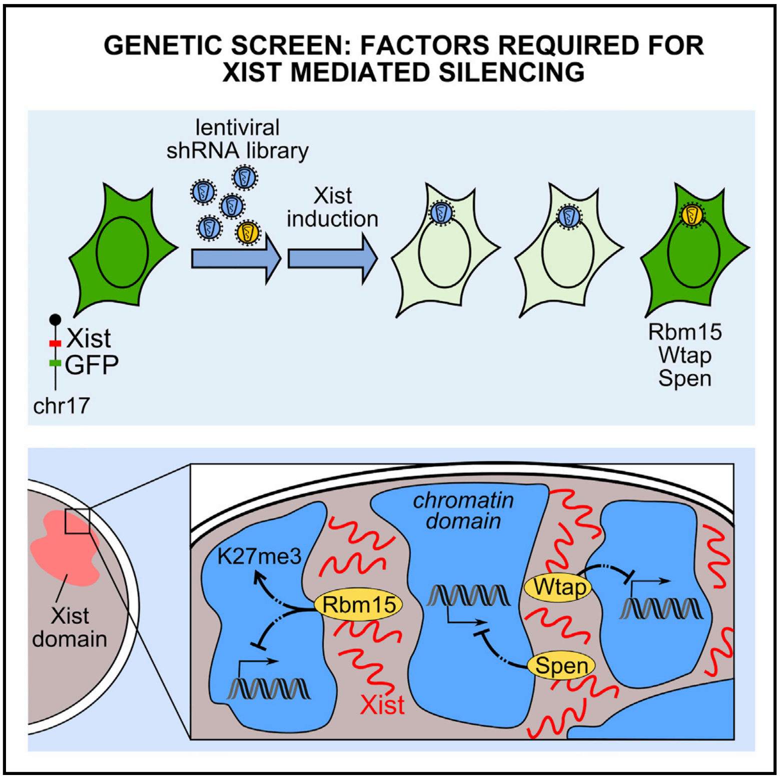

Highlights

- An shRNA screen identifies factors implicated in chromosome silencing by Xist RNA
- Rbm15, Wtap, and Spen are required for Xist-mediated silencing
- Rbm15 is important for efficient deposition of H3K27me3 on the inactive chromosome
- Rbm15, Wtap, and Spen co-localize with Xist RNA in perichromatin spaces

Authors

Benoit Moindrot, Andrea Cerase, Heather Coker, ..., Lothar Schermelleh, Tatyana B. Nesterova, Neil Brockdorff

Correspondence

neil.brockdorff@bioch.ox.ac.uk

In Brief

To identify primary silencing factors implicated in X-chromosome inactivation, Moindrot et al. set up a pooled shRNA genetic screen. The RNA-binding proteins Rbm15 and Spen, together with Wtap, a subunit of the RNA methylation complex, were identified as important factors required for Xist-mediated silencing.

CrossMark

Moindrot et al., 2015, Cell Reports *12*, 562–572
July 28, 2015 ©2015 The Authors
http://dx.doi.org/10.1016/j.celrep.2015.06.053

CellPress

OPEN ACCESS
CellPress

Cell Reports
Article

A Pooled shRNA Screen Identifies Rbm15, Spen, and Wtap as Factors Required for Xist RNA-Mediated Silencing

Benoit Moindrot,[1,4] Andrea Cerase,[1,4,5] Heather Coker,[1] Osamu Masui,[3] Anne Grijzenhout,[1] Greta Pintacuda,[1] Lothar Schermelleh,[2] Tatyana B. Nesterova,[1] and Neil Brockdorff[1,*]
[1]Developmental Epigenetics, Department of Biochemistry, University of Oxford, South Parks Road, Oxford, OX1 3QU, UK
[2]Advanced Cellular Imaging, Department of Biochemistry, University of Oxford, South Parks Road, Oxford, OX1 3QU, UK
[3]Laboratory for Developmental Genetics, RIKEN Center for Integrative Medical Sciences, 1-7-22 Suehiro, Tsurumi-ku, Yokohama 230-0045, Japan
[4]Co-first author
[5]Present address: EMBL Monterotondo, Adriano Buzzati-Traverso Campus, Via Ramarini 32, 00015 Monterotondo, Italy
*Correspondence: neil.brockdorff@bioch.ox.ac.uk
http://dx.doi.org/10.1016/j.celrep.2015.06.053
This is an open access article under the CC BY license (http://creativecommons.org/licenses/by/4.0/).

SUMMARY

X-chromosome inactivation is the process that evolved in mammals to equalize levels of X-linked gene expression in XX females relative to XY males. Silencing of a single X chromosome in female cells is mediated by the non-coding RNA Xist. Although progress has been made toward identifying factors that function in the maintenance of X inactivation, the primary silencing factors are largely undefined. We developed an shRNA screening strategy to produce a ranked list of candidate primary silencing factors. Validation experiments performed on several of the top hits identified the SPOC domain RNA binding proteins Rbm15 and Spen and Wtap, a component of the m6A RNA methyltransferase complex, as playing an important role in the establishment of Xist-mediated silencing. Localization analysis using super-resolution 3D-SIM microscopy demonstrates that these factors co-localize with Xist RNA within the nuclear matrix subcompartment, consistent with a direct interaction.

INTRODUCTION

Dosage compensation in mammals is achieved through the stable silencing of one of the two X chromosomes in female cells during early development, a process termed X-chromosome inactivation (XCI). XCI is initiated by the X inactive specific transcript (Xist), a long non-coding RNA (lncRNA) that is expressed from the inactive X (Xi) elect. Xist RNA spreads in *cis* over the length of the chromosome, and its accumulation triggers the formation of a stable heterochromatic structure, the Barr body (reviewed in Gendrel and Heard, 2014). Xist is both necessary and sufficient to initiate the X-inactivation process (Penny et al., 1996, Lee et al., 1996, Herzing et al., 1997), and indeed Xist transgenes function efficiently when located on autosomes (Lee et al., 1996, Wutz and Jaenisch, 2000). Xist functions in a context-dependent manner during a restricted window of opportunity in early development (Wutz and Jaenisch, 2000; Savarese et al., 2006). Functional dissection of Xist RNA has identified a critical element, the A repeat, required for chromosome inactivation, and multiple redundant elements that mediate localization in *cis* (Wutz et al., 2002).

Formation of the Barr body is a multistep process involving several pathways linked to formation of repressive heterochromatin. Notable examples are the acquisition or loss of specific histone tail modifications, enrichment or depletion of variant histones, DNA methylation at X-linked promoter CpG islands, and long-range topological reorganization of the chromatin fiber. How these different pathways are linked to Xist RNA accumulation and to one another remains poorly understood. A key challenge has been to identify the primary factors that initiate the silencing cascade during the early developmental window of opportunity. A priori, these factors are predicted to include one or more RNA binding proteins (RBPs). Polycomb repressive complex 2 (PRC2), which mediates the histone modification H3 lysine 27 methylation, has been suggested as a candidate for this role (Silva et al., 2003; Plath et al., 2003) and has been proposed to bind directly to Xist RNA (Zhao et al., 2008). However, recent evidence indicates that PRC2 and Xist RNA are spatially separated (Cerase et al., 2014) and further that PRC2 recruitment is mediated by a region of the transcript that is separate from the critical A-repeat element (da Rocha et al., 2014). Two other candidate factors that have been identified are the nuclear matrix proteins SATB1 and the RBP SAFA/hnRNPU. SATB1 has been suggested to have an indirect role in conferring competence for Xist silencing within the developmental window of opportunity (Agrelo et al., 2009). SAFA/hnRNPU, on the other hand, has been suggested to bind directly to Xist RNA via its RRM domain and to facilitate Xist RNA localization (Hasegawa et al., 2010). A role for the nuclear matrix in Xist-mediated silencing is further indicated by the observation that Xist RNA domains are retained in nuclear matrix preparations (Clemson et al., 1996).

OPEN
ACCESS
CellPress

Moreover, imaging of Xist RNA by super-resolution 3D-SIM microscopy (SR-3DSIM) demonstrates localization within perichromatin spaces, together with SAFA/hnRNPU (Smeets et al., 2014).

Efforts to purify native Xist ribonucleoprotein complexes have been hampered by the association of Xist RNA with the insoluble nuclear matrix. Similarly, an attempt to purify key factors from nuclear extracts using defined regions of Xist identified only hnRNPC, a generic mRNA processing factor (Brown and Baldry, 1996). Two independent studies have applied RNAi-based genetic screening to identify factors involved in X inactivation in XX somatic cells (Chan et al., 2011; Bhatnagar et al., 2014). Both studies identified several candidates, and in some cases, validation experiments supported a role in X inactivation and/or Xist expression. However, none of the validated candidates was a known RBP. In this study, we set out to identify the primary silencing factors that act in the critical developmental window of opportunity using a pooled shRNA genetic screen. Several of the targets that we identified were related, either as components of the same multisubunit complexes or defined pathways, suggesting that the screen achieved a high degree of saturation. We validated several top-ranking hits, of which Rbm15 and Spen, two related RBPs, and Wtap a subunit of the N6-methyladenosine (m6A) RNA methylation complex, were all found to play a role in Xist-mediated silencing. SR-3DSIM analysis of Rbm15, Spen, and Wtap demonstrated co-localization with Xist in nuclear matrix/perichromatin spaces, indicating that these factors may interact directly with Xist RNA.

RESULTS

Pooled shRNA Screening

In order to identify factors that function in the critical developmental window of opportunity, we established a reporter system in mouse embryonic stem cells (mESCs), which are known to be Xist responsive (Wutz and Jaenisch, 2000; Tang et al., 2010; Cerase et al., 2014). We made use of an XY mESC cell line, 3E, in which a doxycycline-inducible Xist transgene is located on chromosome 17 (Tang et al., 2010; Cerase et al., 2014). An unstable PEST-GFP open reading frame (Corish and Tyler-Smith, 1999) was inserted under the control of Mylc2b promoter in *cis* with the Xist transgene using homologous recombination to generate the MG-3E cell line (Figures 1A and S1). The Mylc2b locus was selected as one of several loci that is efficiently silenced following 3 days of Xist RNA induction in undifferentiated 3E ESCs (Tang et al., 2010; Cerase et al., 2014). Consistent with expectations, MG-3E cells showed strongly reduced Mylc2b-GFP levels following induction of Xist RNA expression, as determined by fluorescence-activated cell sorting (FACS) analysis (Figure 1B).

We adopted a pooled lentiviral shRNA screening strategy (Silva et al., 2008; Sims et al., 2011) using a custom nucleome shRNA library comprising up to nine independent shRNA hairpins for each of 5,088 target genes encoding mouse proteins with the Gene Ontology (GO) term nucleus. Additionally, a pilot screen using a commercial whole genome shRNA library identified targets in the ubiquitylation pathway (data not shown), and we therefore performed a parallel experiment using a custom designed library of shRNAs directed at ~1,000 target genes encoding mouse proteins with a function in ubiquitylation/sumolyation. A proportion of shRNAs were present in both ubiquitylome and nucleome libraries. Each shRNA was tagged with a unique barcode, enabling subsequent identification by high-throughput sequencing (HTS).

Following optimization and establishment of appropriate conditions, both libraries were screened to identify targets for which knockdown enhanced representation in GFP high cells following induction of Xist RNA (Figure 1C). Briefly, cells were transduced with lentiviral packaged shRNA pools of 9,000–15,000 shRNAs, at low multiplicity of infection (MOI), and then selected for puromycin resistance for 4 days. After the first 24 hr of puromycin selection, doxycycline was added to induce Xist RNA for a total of 3 days, at which point cells were harvested and FACS sorted based on high GFP fluorescence. The representation of shRNAs in FACS sorted and unsorted populations was determined by HTS of PCR products spanning the barcode (Figure 1D; Table S1). To identify putative Xist silencing factors, we established a pipeline to prioritize hits based first on the ratio of barcode sequence in sorted and unsorted populations and then, for a given factor, on the number of independent shRNAs overrepresented in the high GFP population (Figure S2A).

Validation of shRNA Screen Hits

A ranked list of the top targets from the nucleome (225) and ubiquitylome (34) libraries is shown in Tables S2 and S3. The highest ranking hits were all identified with multiple independent shRNAs, precluding off-target effects. There was a good correlation for individual shRNAs designed to ranked targets that are represented in both the nucleome and ubiquitylome, specifically Lonp2, Topors, Senp2, and Usp7 (Figure S2B), indicating reproducibility between independent experiments. Interestingly, we identified several factors associated with specific biochemically defined multisubunit complexes and/or specific pathways. Thus, among the top 30 ranked hits in the nucleome screen, we identified multiple subunits of Mediator complex, which is required for transactivation of RNA Polymerase II, as well as the RNA export factor Nxt1 and the SPOC domain protein Rbm15, which have been shown to interact with one another (Lindtner et al., 2006), together with Spen, an Rbm15 homolog. We also identified Wtap and Virilizer proteins, core subunits of the m6A RNA methyltransferase complex (Ping et al., 2014), different factors implicated in mRNA splicing/biogenesis, and several peroxisomal proteins that had an incidental nuclear GO annotation (Figure 2). Other components of the mRNA export complex (Nxt2#72 and Nxf1#103) and Mediator complex (Med10#208) were also identified (Table S2). Additional targets of potential interest included PRC1 Polycomb proteins (KDM2B#79, Scml2#168, and L3Mbtl3#178), other factors linked to heterochromatin (Prmt1#22 and Mbd3#51), nuclear matrix/chromosome structure proteins (Matr3#20 and Topors#23), and a pluripotency factor (Dppa2/4#16). Within the ubiquitylome screen, we identified the deubiquitylase Usp9x (#3), which had also been found in pilot screens performed using commercial whole-genome shRNA libraries (data not shown) and several subunits of the Cop9 signalosome (Table S3, #7, #9, #11 and #29).

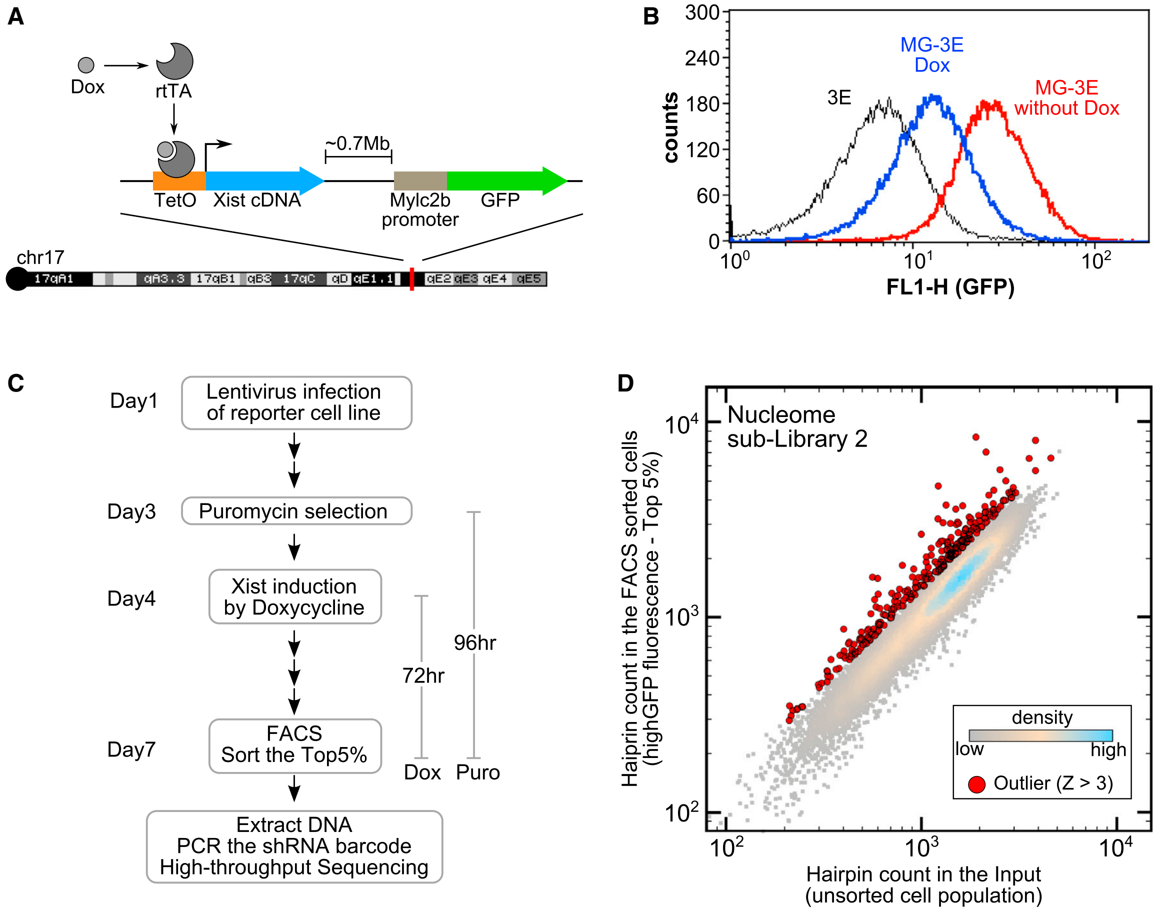

Figure 1. A Genetic Screen to Identify Silencing Factors Acting during the Establishment of XCI

(A) Arrangement of the engineered chromosome 17 loci in the MG-3E reporter cell line. See also Figure S1.

(B) GFP fluorescence of MG-3E cell line before (red) and after (blue) doxycycline treatment (72 hr, 1.5 μg/ml). The parental 3E cell line (black) is also shown.

(C) Workflow for the genetic screen. MG-3E cells were transduced with shRNA libraries 3 days prior to Xist induction. Transduced cells were FACS sorted 72 hr after Xist induction. PCR across the shRNA barcode is performed on DNA from sorted and unsorted MG-3E cells and PCR products analyzed by HTS.

(D) Hairpin count in unsorted and sorted MG-3E cells transduced with one of the three nucleome sublibraries. The outliers enriched in the sorted samples are indicated in red ($z \geq 3$).

Selected high-ranked targets were validated by FACS analysis of Mylc2b-GFP following transduction of induced and non-induced MG-3E cells with individual shRNAs and scrambled control (Figures 3A, 3B, and S3). A luciferase shRNA, together with a second scramble shRNA, were used as negative controls (Figures 3B and S3D). RT-PCR and/or antibody assays were used to determine the knockdown efficiency for the different shRNAs (Figures S4A–S4C). In several cases, for example, Med16, Rbm15, Spen, and Wtap, we observed enhanced GFP levels relative to the scrambled control following Xist RNA induction, demonstrating that knockdown of these factors indeed affects Xist-mediated repression of the GFP reporter (Figures 3B and S3A). In other cases, for example, the peroxisomal protein Lonp2 and Virilizer, we observed enhanced Mylc2b-GFP levels also in uninduced cultures (Figure S3B), indicating that the effect is, at least in part, independent of silencing by Xist RNA. This could occur, for example, through stabilization of GFP or GFP encoding mRNA. It should be noted that this does not rule out a role for these factors in Xist-mediated silencing. In the case of Nxt1, shRNA transduction severely reduced the viability of the cells, presumably because Nxt1 is required for the nuclear export of mRNAs. Because reduced viability could lead to counterselection that would bias the validation, we did no further analysis of this target. Finally, for Usp9x, ranked #3 in the ubiquitylome screen, we observed a relatively weak enhancement of Mylc2b GFP levels specifically in Xist-induced cells (Figure S3C).

The aforementioned FACS validation experiments highlighted Mediator, Rbm15, Spen, and Wtap as potentially having a role in Xist-mediated silencing. A priori, the knockdown of these positive hits could affect Xist transgene expression, Xist RNA localization or interfere with the downstream silencing cascade. We therefore analyzed Xist RNA expression using RNA FISH (Figure 3C) and RT-PCR (Figure S3E) following shRNA knockdown in induced MG-3E cells. Knockdown of the Mediator subunit Med16 resulted in a clear reduction in Xist domains and levels of Xist RNA. This is most likely due to dependence of doxycycline

OPEN ACCESS
CellPress

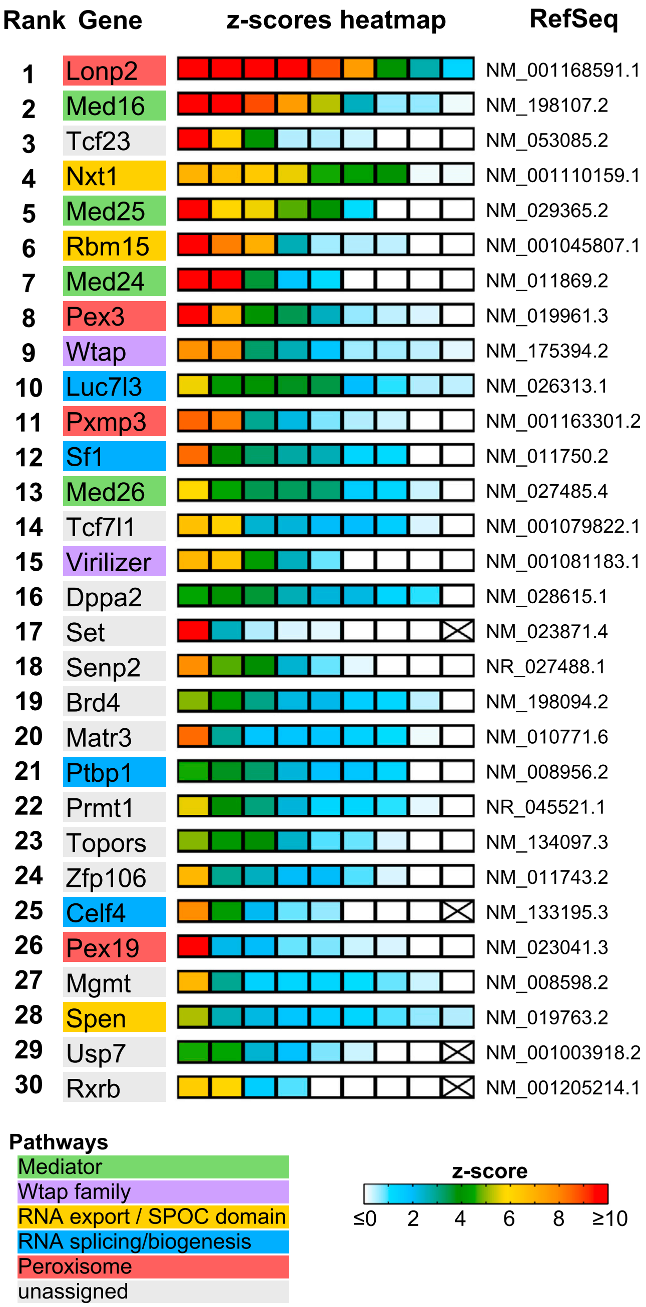

Figure 2. Top-Ranked Candidates from the Nucleome Screen

The 30 top-ranked candidates from the nucleome screen are listed. The enrichment in the FACS-sorted cells (*Z*-score) of the corresponding hairpins is shown using a colored heatmap, where each square represents an independent hairpin. Crosses indicate excluded hairpins for which read number was below a set threshold. The candidates were ranked based on the *Z*-score for the hairpins targeting the same transcript. Factors linked to the same complexes/pathways are color coded. See also Figure S2.

inducible transgene expression on co-activation by VP16, which in turn requires interaction with the Med25 subunit of the mediator complex tail region (Yang et al., 2004). Consistent with this suggestion, the majority of Mediator subunits identified in the screen are in the tail region and include Med25 (Figure 3A) (Malik and Roeder, 2010). We conclude that Mediator complex is important for the function of the MG-3E reporter system rather than for Xist-mediated silencing per se.

For Rbm15, Spen, and Wtap, Xist domains were apparently unaffected, although for Rbm15, levels of Xist RNA determined by RT-PCR were somewhat reduced (Figures 3C and S3E). Thus, these results indicate that Rbm15, Spen, and Wtap function primarily in Xist-mediated silencing and not in Xist RNA localization.

Rbm15, Spen, and Wtap Are Required for Xist-Mediated Silencing

We went on to determine whether Rbm15, Spen, and Wtap have a role in silencing of other genes located in *cis* with the Xist transgene. Initially we used RT-PCR to assess expression levels of four chromosome 17 genes, SatB1, Enpp5, Crb3, and Fbxl17, previously shown to be downregulated following Xist RNA induction in MG-3E cells (Cerase et al., 2014). As shown in Figure S4D, knockdown of Rbm15, Spen, and Wtap and also Med16, used as a positive control, all resulted in elevated levels of the analyzed genes. While these effects varied from gene to gene, and between different shRNAs, control loci on other chromosomes, Dnmt1 and rtTA, were unaffected. Overall the results are consistent with knockdown of Rbm15, Spen, and Wtap affecting Xist-mediated silencing of the whole of chromosome 17.

To substantiate our findings using RT-PCR analysis, we assayed allelic silencing of Fbxl17 by RNA FISH, determining presence or absence of Fbxl17 nascent mRNA foci within or immediately adjacent to doxycycline-induced Xist RNA domains following transduction with Rbm15, Spen, Wtap, or scrambled shRNAs. The results, shown in Figure 4A, demonstrate that the frequency of Fbxl17 nascent RNA foci associated with Xist RNA domains is significantly and reproducibly elevated following knockdown of all three factors.

To determine the role of Rbm15, Spen, and Wtap in Xist-mediated gene silencing on the X chromosome, as opposed to in an Xist transgene model, we performed RNA FISH analysis of Xist RNA and nascent mRNA for two X-linked genes, Pgk1 and Rnf12, in differentiating XX embryonic stem cells (ESCs). As shown in Figure 4B, we observed a significant increase in the frequency of nascent mRNA foci associated with Xist domains for both genes following knockdown of Rbm15, Spen, and Wtap, as compared with scrambled shRNA control. Thus, together these experiments demonstrate a key role for Rbm15, Spen, and Wtap in gene silencing in *cis* mediated by Xist RNA.

We went on to determine whether knockdown of Rbm15, Spen, and Wtap affect chromatin features of Xi, specifically the formation of H3K27me3 domains linked to Xist-mediated recruitment of the Polycomb complex PRC2. Initial analysis indicated Rbm15 knockdown leads to reduced intensity and size of H3K27me3 domains (Figure 5A). No obvious effect was seen with either Spen or Wtap knockdown (Figure S5A). To quantify the effect seen following Rbm15 knockdown, we developed a semiquantitative image analysis pipeline, defining four categories, strong, intermediate, weak, and absent K27me3 domains (Figure 5B). Knockdown of Med16 was again used as a control. As shown in Figure 5C, we observed a consistent reduction in the

OPEN ACCESS
CellPress

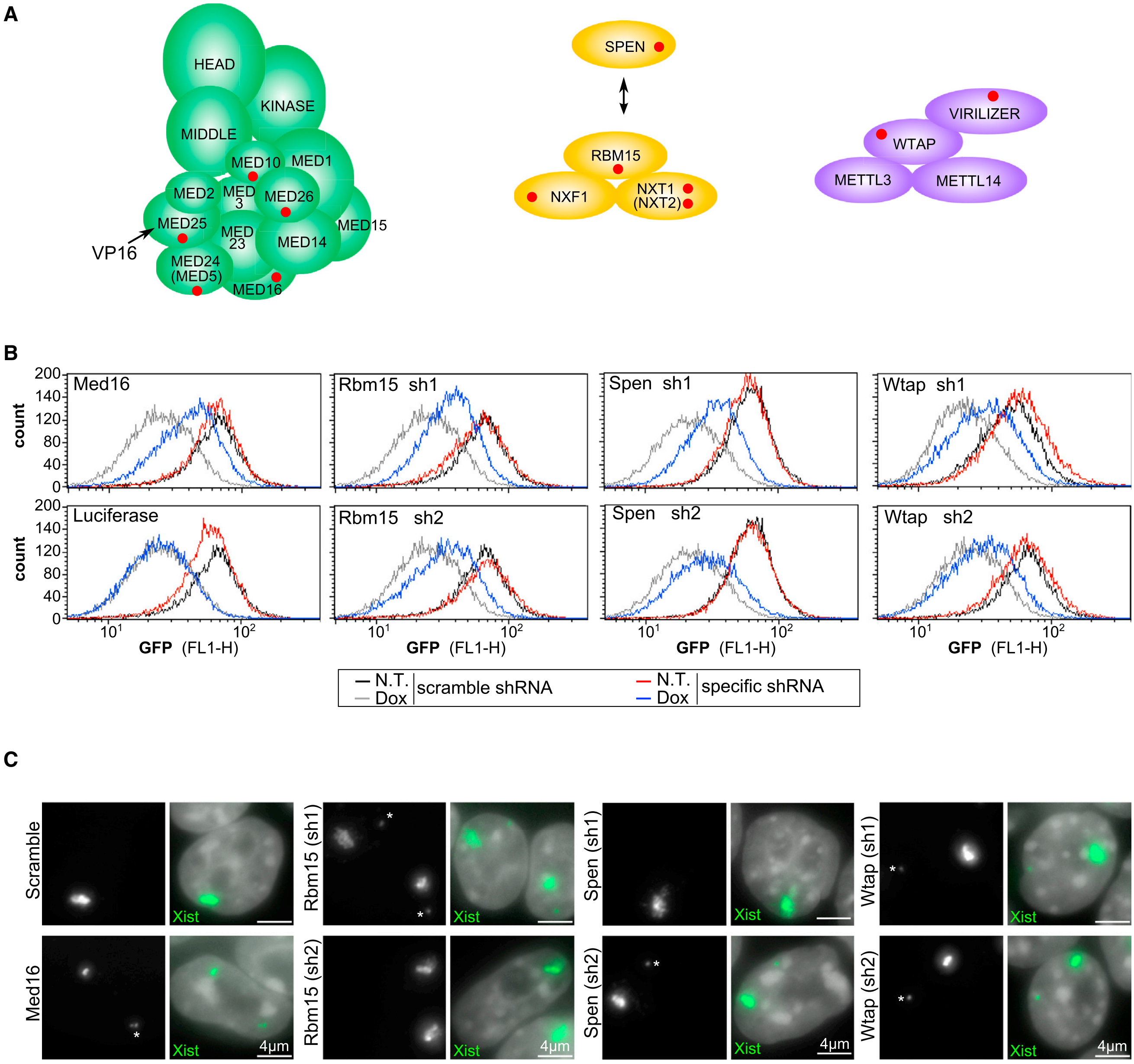

Figure 3. Validation of Selected High-Ranking Targets

(A) Molecular complexes from the nucleome library: the Mediator complex (left); an RNA export complex composed of Nxf1, Nxt1, and the SPOC domain proteins Rbm15 and Spen (middle); the m6A methylation complex with Wtap and Virilizer (right). Subunits identified in the screen are labeled with a red dot.

(B) GFP fluorescence of MG-3E reporter cell line with (gray/blue, 72 hr) or without (black/red) doxycycline, transduced with scramble (black/gray) or specific shRNA targeting Med16, Luciferase as a control, Rbm15, Wtap, and Spen.

(C) RNA FISH illustrating that knockdown of Med16 impairs Xist domain formation, whereas Rbm15, Wtap, or Spen knockdown has no detectable effect. RNA FISH was performed 24 hr after doxycycline treatment. The asterisk indicates Tsix RNA foci that sometimes lie in the same focal plane.

Scale bars are 4 μm. See also Figure S3.

size of H3K27me3 domains using three independent Rbm15 hairpins. The level of reduction approached that seen following knockdown of the Med16 subunit that significantly reduces transgenic Xist RNA expression as reported above. We conclude that Rbm15 is important for efficient establishment of H3K27me3 domains on Xi.

Super-resolution 3DSIM Reveals Rbm15, Spen, and Wtap Co-localize with Xist RNA

To further examine the function of Rbm15, Spen, and Wtap in Xist-mediated silencing, we used immunofluorescence to assess their nuclear localization in 3E mESCs expressing transgenic Xist RNA. The results, illustrated in Figure S5B, show that

OPEN ACCESS
CellPress

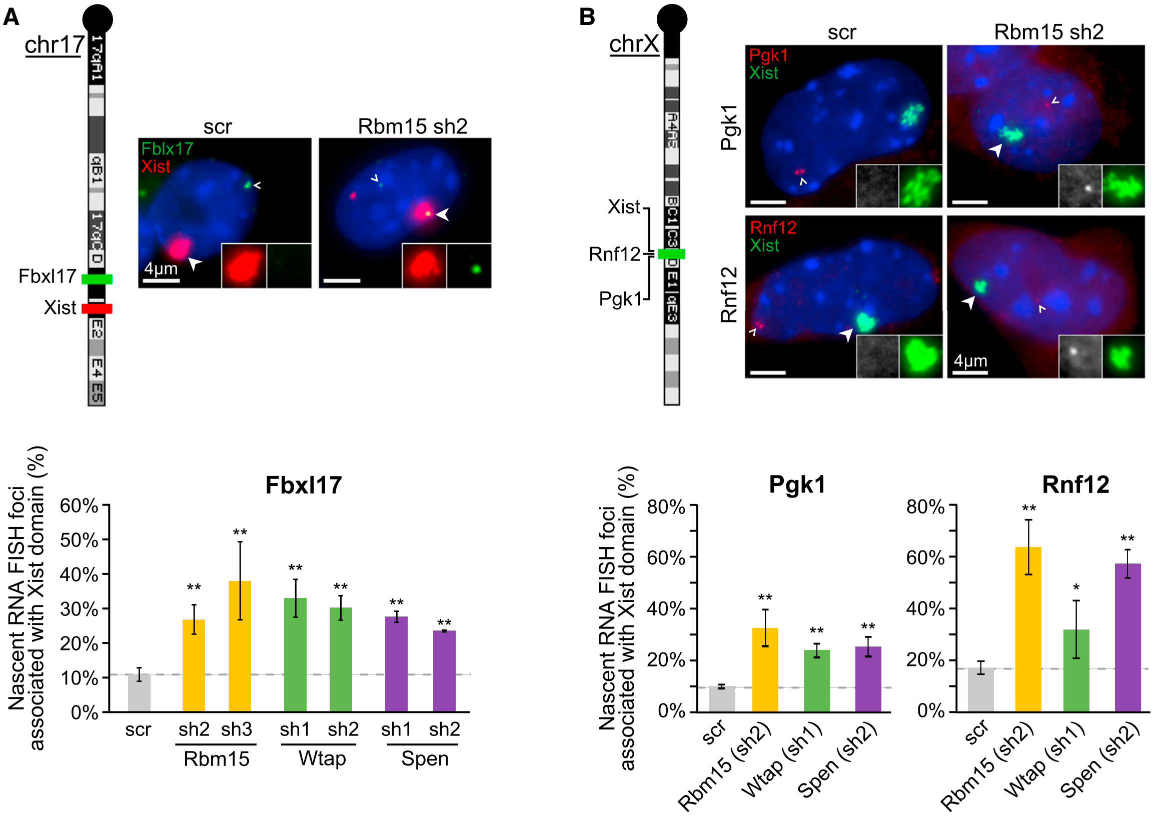

Figure 4. Rbm15, Wtap, and Spen Are Required for Xist-Mediated Transcriptional Silencing

(A) Expression of Fbxl17 gene (green) was assessed within the Xist-coated chromosome (red) by nascent RNA FISH in 3E ESCs. (Top) The relative positions of Fbxl17 and Xist transgene are shown on the chromosome 17 ideogram. Images of individual cells after treatment with scramble or Rbm15 shRNAs. The insets correspond to a 1.75× magnification of the Xist cloud and show both red and green channels. (Bottom) Quantification (mean ± SD) of the proportion of cells with Fbxl17 allele expressed from the Xist-coated chromosome after treatment with different shRNAs. More than 150 cells from three independents experiments were scored.

(B) Expression level of Pgk1 and Rnf12 genes (red) was assessed within the Xist-coated chromosome (green) by nascent RNA FISH in differentiated XT67E1 female ESCs. Relative positions of genes on chromosome X and example RNA FISH images with 1.75× magnification showing Xist in green and assessed genes in gray. Quantifications (mean ± SD) are shown below and were performed on more than 140 cells from three independents experiments.

*$p < 0.05$, **$p < 0.005$ relative to scr shRNA (chi-square test). Scale bars are 4μm. The full arrowheads indicate the Xist cloud magnified in the insets; the open arrowheads indicate the expressed genes from the homologous chromosome. See also Figure S4.

all three factors have a broad nuclear localization, with neither enrichment nor exclusion underlying Xist RNA domains, as assessed by co-staining for H3K27me3.

Analysis of Xi features and Xist RNA by super-resolution 3DSIM has shown that Xist RNA localizes to the perichromatin or nuclear matrix compartment, spatially separated from chromatin (Smeets et al., 2014). To analyze the localization patterns of Rbm15, Spen, and Wtap relative to Xist RNA, we made use of an ESC line, P4D7B1, in which inducible Xist RNA is tagged with Bgl stem loops that bind a BglG-mCherry fusion protein (Chen et al., 2009) (Figure 6A). This system bypasses the requirement to prepare samples using the relatively disruptive immuno-FISH procedure. As shown in Figure 6B, BglG-mCherry signal accurately recapitulates Xist RNA localization within perichromatin spaces and clearly separated from chromatin.

We went on to determine the relative localization of BglG-mCherry and Rbm15 (Figure 6C), Wtap (Figure 6D), and Spen (Figure 6E) in P4D7B1 cells. As a control, we analyzed the PRC2 Polycomb protein Ezh2 (Figure 6F). The channel alignment for SR-3DSIM is shown in Figure S6. All three of the Xist silencing factors localized to perichromatin spaces, both within the Xist expression domains, and at other nuclear sites (Figures 6C–6E). Moreover, within the Xist expression domains, we observed extensive co-localization of the two signals (Figures 6C–6E, right). In contrast, BglG-mCherry and Ezh2 signal were on the whole spatially separated, consistent with our previous observations (Cerase et al., 2014). These findings demonstrate that Rbm15, Wtap, and Spen function within the same nuclear subcompartment in which Xist RNA is localized and therefore support that these factors could potentially interact with Xist RNA.

DISCUSSION

The genetic screen described here was designed to identify factors required for establishment of Xist-mediated silencing.

OPEN
ACCESS
CellPress

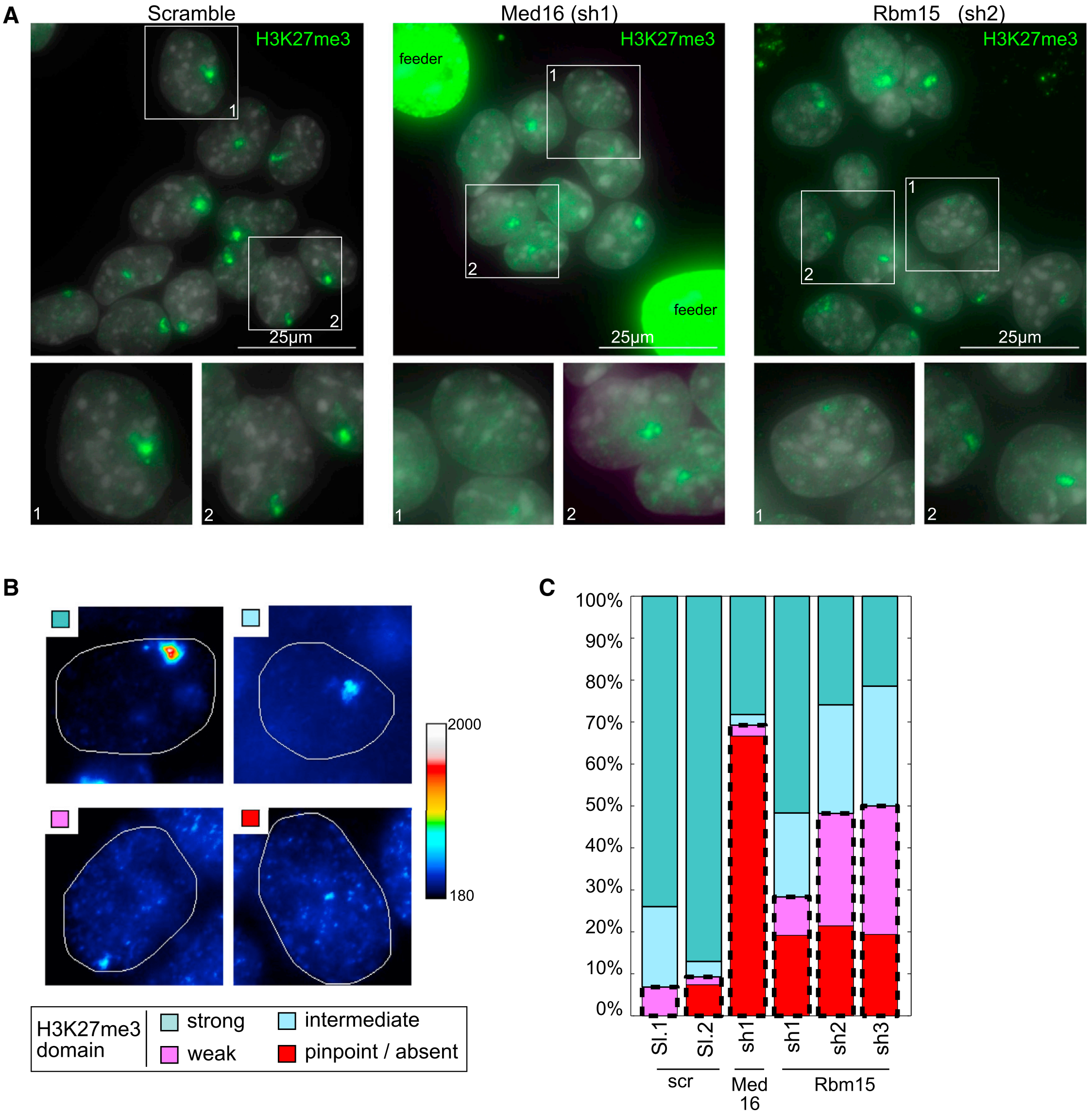

Figure 5. H3K27me3 Domains Are Altered by Rbm15 Knockdown

(A) H3K27me3 domains in 3E cells treated with scrambled, Med16 or Rbm15 targeting shRNA. Xist was induced for 24 hr before the IF. The inserts (below) show magnifications.

Scales bar are 25 μm. See also Figure S5A.

(B) H3K27me3 domains were classified into four categories based on the color map (right).

(C) Percentage of cells with strong/intermediate/weak/absent H3K27me3 domains as defined in (B); 70–120 cells were analyzed for each shRNA.

Specifically, the reporter cell system functions within the critical developmental window of opportunity during which cells are Xist responsive. The use of an inducible Xist transgene system and of an unstable PEST-GFP reporter enabled us to focus the screen on the time period during which Xist-mediated silencing is initiated. The fact that we identified different subunits of defined complexes and also multiple factors linked to specific pathways indicates that the screen achieved a good degree of saturation. However, we cannot rule out that some factors evaded detection, for example, because of functional redundancy or incomplete coverage of the shRNA libraries.

Pooled shRNA knockdown offered specific advantages in the context of this screen. First, loss of function occurs across a broad dynamic range (because of cell-to-cell variation in knockdown efficiency and between different hairpins designed to the same gene), creating a virtual allelic series that facilitates identification of essential factors for which significant loss of function affects cell viability. Second, using a number of shRNA hairpins to each target provided a critical parameter for the ranking of positive hits.

Our screening procedure was developed after extensive optimization. Notably, we found that it was important to ensure that

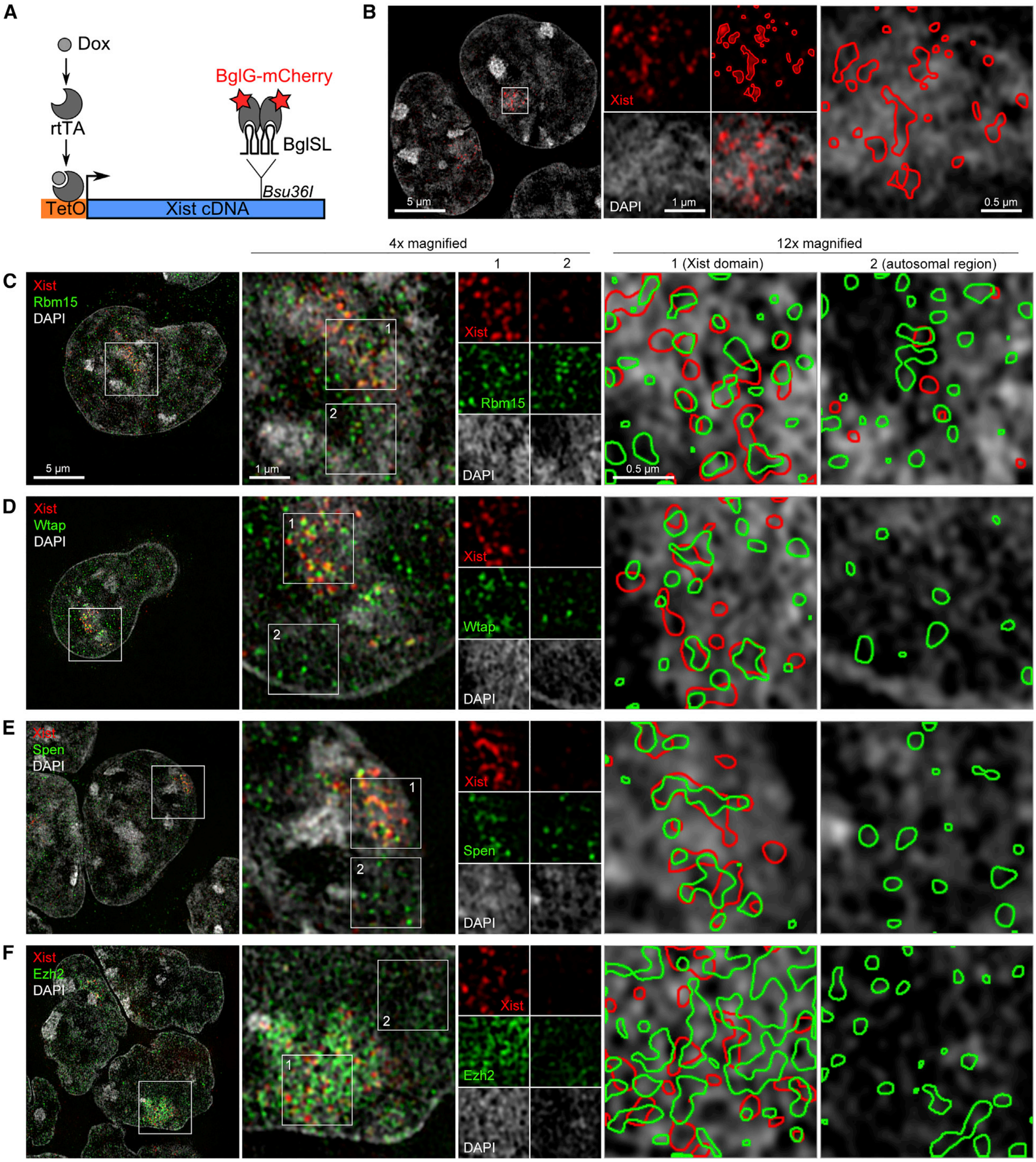

Figure 6. 3DSIM Showing that Xist RNA, Rbm15, Wtap, and Spen Co-localize within Perichromatin Spaces
(A) Xist tagging strategy using 18 copies of the Bgl stem-loop (BglSL) motif, which are recognized by the protein fusion BglG-mCherry.
(B) Indirect detection of Xist RNA by immunofluorescence using an anti-mCherry antibody illustrates its localization within perichromatin spaces.
(C–F) Double IF to analyze the distribution of Xist (red) and Rbm15 (C), Wtap (D), Spen (E), and the PRC2 component Ezh2 (F) (green). The 4×-magnified panels show the merged image. Insets 1 and 2, respectively, correspond to the Xist domain and the neighboring autosomal chromosome. The 12×-magnified panels illustrate the respective DAPI signal with outlined Xist (red) and analyzed protein (green) localization.
See also Figure S6.

the number of cells transduced with a given shRNA was sufficient to reliably detect overrepresentation in the selected populations, particularly given that lentiviral transduction of ESCs is relatively inefficient. Linked to this, it was important to limit the coverage of the screen to factors in the nucleome and ubiquitylome in order to reduce false positive rates. Finally, inclusion of an HTS barcode in the shRNAs was essential to obviate the need to sequence across stem loops, which can introduce extreme bias.

Possible sources of false positives in the screen include knockdown of factors that affect the TetOn-inducible promoter system used to drive Xist expression in the reporter cell line and factors that influence the levels of the GFP-PEST reporter other than at the level of transcriptional silencing. Using validation assays that discriminate these possibilities, we found that Mediator knockdown strongly reduces TetOn promoter-driven Xist RNA expression, most likely linked to its requirement for VP16 mediated transactivation, and that knockdown of core peroxisome proteins increased levels of GFP-PEST protein, possibly linked to defects in protein turnover. Although we discarded these hits as false positives, the fact that we identified multiple factors in the same complex/pathway in both cases further demonstrates that the screen achieved a high degree of saturation.

Rbm15, Spen, and Wtap Facilitate Xist-Mediated Silencing

The validation of top-ranked hits identified three factors, Rbm15, Spen, and Wtap, as playing a role in Xist-mediated silencing. Interestingly, there is evidence that these factors may interact with one another (Horiuchi et al., 2013; Malovannaya et al., 2011). Knockdown of all three factors suppressed Xist-mediated silencing in *cis* but had no obvious effect on the formation of Xist RNA domains. In the case of Rbm15, we also observed a deficiency in the formation of Xist-mediated H3K27me3 domains. SR-3DSIM analysis revealed co-localization with Xist RNA within the nuclear matrix/perichromatin compartment. Given that Rbm15 and Spen are RBPs, our SR-3DSIM observations are consistent with a direct role in binding Xist RNA. This proposal is further supported by two very recently published studies that identified Spen, Wtap, and Rbm15 among several proteins that crosslink to Xist RNA following either formaldehyde (Chu et al., 2015) or UV treatment (McHugh et al., 2015). Of note, both Rbm15 and Spen interactions were found using UV crosslinking (McHugh et al., 2015), supporting that they bind to Xist RNA directly. Both of these studies found that Spen is important for Xist-mediated silencing, in agreement with our observations. However, McHugh et al. (2015) did not detect silencing defects following knockdown of Rbm15, contrasting with our results. The reason for this discrepancy is unknown but could relate to the use of different shRNAs and/or silencing assays.

A comparative analysis of our results and those from the proteomic studies is provided in Table S4. In addition to the major candidates, the RBP Ptbp1, ranked 21 on our list, was identified as a direct Xist interactor in McHugh et al. (2015) and Chu et al. (2015), and the nuclear matrix protein Matr3, ranked 20 in our screen, was identified in Chu et al. (2015). The overlap between our analysis and the proteomic-based studies indicates that a good proportion of candidates identified but not yet validated in this study also function in the X-inactivation pathway. Of particular interest, several candidate factors highly ranked in our study were not identified in the proteomic screens. Notable examples are the RNA export factors Nxt1 and Nxf1/2, proposed Rbm15 interactors, and Virilizer, which interacts with Wtap. It should also be noted that several factors identified in the proteomic analyses were not present in our top-ranked list (Table S4). Of particular note, shRNAs for lamin B receptor (LBR), suggested as a key silencing factor by McHugh et al. (2015), and hnRNPK, which in Chu et al. (2015) was reported to contribute to silencing, showed no significant enrichment. Together, these examples highlight key similarities and differences that should be investigated in future work.

The mechanism of action of Rbm15, Spen, and Wtap in Xist-mediated silencing remains to be fully elucidated. Rbm15 and Spen are both Spen paralogue and orthologue C-terminal (SPOC) domain proteins, possibly indicating a common mode of action. The SPOC domain of Spen has been reported to interact with the co-repressor NCoR/SMRT, together with the histone deacetylase Hdac3 (Shi et al., 2001), and McHugh et al. (2015) found that knockdown of NCoR/SMRT and Hdac3 abrogates Xist-mediated silencing. In this regard, it is noteworthy that shRNAs for NCoR/SMRT and Hdac3 were not enriched in our analysis (Table S4). Similarly, the Rbm15 SPOC domain has been reported to interact with the histone H3K4 methyltransferase SET1B (Lee and Skalnik, 2012), but SET1B shRNAs are also not enriched in our screen. An alternative model for the role of Rbm15 in Xist-mediated silencing is that it functions in complex with the RNA export factors Nxt1/2 and Nxf1. This could potentially link to the fact that Xist RNA is retained in the nucleus, despite being spliced and polyadenylated (Brockdorff et al., 1992; Brown et al., 1992).

Wtap and Virilizer are subunits of the m6A methylation complex, important for the regulation of mRNA stability (Schwartz et al., 2014; Geula et al., 2015). Additionally, a recent study has shown that m6A methylation modifies RNA structure to facilitate binding of the hnRNPC protein required for RNA maturation (Liu et al., 2015). Based on this, we speculate that m6A methylation of Xist RNA may be important to enable binding of silencing factors such as Rbm15 and Spen. An important caveat, however, is that Mettl3, one of the catalytic subunits of the m6A methyltransferase complex, was not present on our ranked list, nor in the aforementioned proteomic screens. Thus, further studies are required to determine whether the link between Wtap and m6A methylation is in fact relevant in the context of Xist mediated silencing.

Linking Xist Function to the Nuclear Matrix

Polycomb repressor proteins and several other factors implicated in Xi chromatin structure show a strong enrichment within Xist silencing domains relative to other regions of the nucleus. Our findings indicate that this is not the case for Rbm15, Spen, and Wtap, even though they clearly co-localize with Xist RNA. A possible explanation is that these factors are constitutive components of a machinery that localizes to perichromatin spaces, which functions, for example, in mRNA surveillance following release from RNA PolII complexes, prior to translocation to the

nuclear pores. Xist RNA may have evolved to interact with this machinery in a manner that triggers a checkpoint that involves both RNA entrapment by nuclear matrix proteins such as hnRNPU/SAFA and signaling back to chromatin to shut down transcription. In relation to the latter, it is interesting to note that Spen is an unusually large protein (450 kDa) and as such could bridge the distance between Xist RNA and underlying chromatin, observed using SR-3DSIM.

In summary, we have used shRNA screening to identify novel factors that function in the establishment of Xist-mediated silencing. The screen achieved a high degree of saturation, indicating that the majority of key factors have been detected. Of particular interest, we validated that Rbm15, Wtap, and Spen are required for Xist-mediated silencing. Given that those three factors have very recently been identified as Xist RNA interacting proteins (Chu et al., 2015; McHugh et al., 2015), we believe that other targets identified in this study provide a rich resource for further investigation of the mechanism of Xist-mediated silencing.

EXPERIMENTAL PROCEDURES

For detailed experimental procedures, see Supplemental Experimental Procedures.

Reporter Cell Line

The reporter cell line MG-3E was derived from XY 3E ESCs (Tang et al., 2010; Cerase et al., 2014) by replacing Mylc2b coding region located in *cis* with the Xist transgene with an unstable GFP (GFP:Pest; Corish and Tyler-Smith, 1999). Full details are provided in Supplemental Experimental Procedures.

Cell Culture and shRNA Screen

ESCs were grown on feeders in DMEM supplemented with leukemia inhibitory factor (LIF)-conditioned medium. Before lentiviral infection, ESCs were trypsinized and pre-plated for 30 min to remove the feeders. For the pooled shRNA libraries 215 × 10^6 (Nucleome sub-libraries) or 126 × 10^6 (Ubiquitylome library) MG-3E cells were seeded in 14-cm dishes in ESC medium containing 8 μg/ml Polybrene. Lentiviruses were then added to the medium and cells grown for 24 hr at 37°C 5% CO_2. The following day, ESCs were trypsinized and seeded on feeders. On day 3, Puromycin selection (2 μg/ml) was initiated and maintained until day 7. From day 4, Xist expression was induced by supplementing the medium with doxycycline (1.5 μg/ml). At day 7, cells were trypsinized and processed for FACS analysis.

Flow sorting (Beckman Coulter MoFlo XDP) was performed on 30–50 million MG-3E ESCs for each of three nucleome sublibraries and the ubiquitylome library. Cells with high GFP fluorescence (upper 5%) were collected. Candidates were identified and ranked based on the hairpin enrichment in the FACS-sorted compared with input populations (see Supplemental Experimental Procedures). A similar protocol was used for individual hairpin transductions, using smaller numbers of cells (the sequences of the shRNA used in validation experiments are listed in Table S5). For immunofluorescence (IF) following lentivirus infection, cells were trypsinized and seeded on slides on day 5, Xist was induced on day 6, and cells were fixed on day 7. For RNA FISH following lentivirus infection in 3E cell lines, cells were induced from day 4, trypsinized and seeded on slides on day 6, and fixed on day 7.

Microscopy

Preparation of cells for RNA-FISH, IF, and 3DSIM was essentially as previously described (Nesterova et al., 2011; Smeets et al., 2014; Cerase et al., 2014). 3DSIM imaging was performed on a DeltaVision OMX V3 Blaze system (GE Healthcare). Modifications to protocols and all further details are provided in Supplemental Experimental Procedures.

SUPPLEMENTAL INFORMATION

Supplemental Information includes Supplemental Experimental Procedures, six figures, and five tables and can be found with this article online at http://dx.doi.org/10.1016/j.celrep.2015.06.053.

AUTHOR CONTRIBUTIONS

A.C, T.B.N., and N.B. conceptualized the experiments. A.C. generated the reporter cell line, optimized and validated the initial screening strategy. B.M. performed and analyzed the genetic screen and validated the hits in the reporter cell line. T.B.N. performed the validations in differentiated ESCs. H.C. and L.S. performed the super-resolution imaging. A.G., G.P. and O.M. provided cell lines and reagents. N.B. wrote the manuscript. B.M., A.C., H.C., G.P., and T.B.N. reviewed and edited the manuscript. The manuscript has been approved by all authors. N.B. acquired and secured the funding.

ACKNOWLEDGMENTS

We wish to thank Chris Tyler-Smith for GFP-PEST plasmid, Nigel Rust from the Flow Cytometry Service in the Sir William Dunn School of Pathology (Oxford), and Francis Barr for mCherry antibody. This work was funded by grants to N.B. (Wellcome Trust 081385,091911) and the Micron advance imaging initiative (Wellcome Trust 103768).

Received: March 10, 2015
Revised: May 21, 2015
Accepted: June 15, 2015
Published: July 16, 2015

REFERENCES

Agrelo, R., Souabni, A., Novatchkova, M., Haslinger, C., Leeb, M., Komnenovic, V., Kishimoto, H., Gresh, L., Kohwi-Shigematsu, T., Kenner, L., and Wutz, A. (2009). SATB1 defines the developmental context for gene silencing by Xist in lymphoma and embryonic cells. Dev. Cell *16*, 507–516.

Bhatnagar, S., Zhu, X., Ou, J., Lin, L., Chamberlain, L., Zhu, L.J., Wajapeyee, N., and Green, M.R. (2014). Genetic and pharmacological reactivation of the mammalian inactive X chromosome. Proc. Natl. Acad. Sci. USA *111*, 12591–12598.

Brockdorff, N., Ashworth, A., Kay, G.F., McCabe, V.M., Norris, D.P., Cooper, P.J., Swift, S., and Rastan, S. (1992). The product of the mouse Xist gene is a 15 kb inactive X-specific transcript containing no conserved ORF and located in the nucleus. Cell *71*, 515–526.

Brown, C.J., and Baldry, S.E. (1996). Evidence that heteronuclear proteins interact with XIST RNA in vitro. Somat. Cell Mol. Genet. *22*, 403–417.

Brown, C.J., Hendrich, B.D., Rupert, J.L., Lafrenière, R.G., Xing, Y., Lawrence, J., and Willard, H.F. (1992). The human XIST gene: analysis of a 17 kb inactive X-specific RNA that contains conserved repeats and is highly localized within the nucleus. Cell *71*, 527–542.

Cerase, A., Smeets, D., Tang, Y.A., Gdula, M., Kraus, F., Spivakov, M., Moindrot, B., Leleu, M., Tattermusch, A., Demmerle, J., et al. (2014). Spatial separation of Xist RNA and polycomb proteins revealed by superresolution microscopy. Proc. Natl. Acad. Sci. USA *111*, 2235–2240.

Chan, K.M., Zhang, H., Malureanu, L., van Deursen, J., and Zhang, Z. (2011). Diverse factors are involved in maintaining X chromosome inactivation. Proc. Natl. Acad. Sci. USA *108*, 16699–16704.

Chen, J., Nikolaitchik, O., Singh, J., Wright, A., Bencsics, C.E., Coffin, J.M., Ni, N., Lockett, S., Pathak, V.K., and Hu, W.S. (2009). High efficiency of HIV-1 genomic RNA packaging and heterozygote formation revealed by single virion analysis. Proc. Natl. Acad. Sci. USA *106*, 13535–13540.

Chu, C., Zhang, Q.C., da Rocha, S.T., Flynn, R.A., Bharadwaj, M., Calabrese, J.M., Magnuson, T., Heard, E., and Chang, H.Y. (2015). Systematic discovery of Xist RNA binding proteins. Cell *161*, 404–416.

Clemson, C.M., McNeil, J.A., Willard, H.F., and Lawrence, J.B. (1996). XIST RNA paints the inactive X chromosome at interphase: evidence for a novel RNA involved in nuclear/chromosome structure. J. Cell Biol. *132*, 259–275.

Corish, P., and Tyler-Smith, C. (1999). Attenuation of green fluorescent protein half-life in mammalian cells. Protein Eng. *12*, 1035–1040.

da Rocha, S.T., Boeva, V., Escamilla-Del-Arenal, M., Ancelin, K., Granier, C., Matias, N.R., Sanulli, S., Chow, J., Schulz, E., Picard, C., et al. (2014). Jarid2 Is Implicated in the Initial Xist-Induced Targeting of PRC2 to the Inactive X Chromosome. Mol. Cell *53*, 301–316.

Gendrel, A.V., and Heard, E. (2014). Noncoding RNAs and epigenetic mechanisms during X-chromosome inactivation. Annu. Rev. Cell Dev. Biol. *30*, 561–580.

Geula, S., Moshitch-Moshkovitz, S., Dominissini, D., Mansour, A.A., Kol, N., Salmon-Divon, M., Hershkovitz, V., Peer, E., Mor, N., Manor, Y.S., et al. (2015). Stem cells. m6A mRNA methylation facilitates resolution of naïve pluripotency toward differentiation. Science *347*, 1002–1006.

Hasegawa, Y., Brockdorff, N., Kawano, S., Tsutui, K., Tsutui, K., and Nakagawa, S. (2010). The matrix protein hnRNP U is required for chromosomal localization of Xist RNA. Dev. Cell *19*, 469–476.

Herzing, L.B., Romer, J.T., Horn, J.M., and Ashworth, A. (1997). Xist has properties of the X-chromosome inactivation centre. Nature *386*, 272–275.

Horiuchi, K., Kawamura, T., Iwanari, H., Ohashi, R., Naito, M., Kodama, T., and Hamakubo, T. (2013). Identification of Wilms' tumor 1-associating protein complex and its role in alternative splicing and the cell cycle. J. Biol. Chem. *288*, 33292–33302.

Lee, J.H., and Skalnik, D.G. (2012). Rbm15-Mkl1 interacts with the Setd1b histone H3-Lys4 methyltransferase via a SPOC domain that is required for cytokine-independent proliferation. PLoS ONE *7*, e42965.

Lee, J.T., Strauss, W.M., Dausman, J.A., and Jaenisch, R. (1996). A 450 kb transgene displays properties of the mammalian X-inactivation center. Cell *86*, 83–94.

Lindtner, S., Zolotukhin, A.S., Uranishi, H., Bear, J., Kulkarni, V., Smulevitch, S., Samiotaki, M., Panayotou, G., Felber, B.K., and Pavlakis, G.N. (2006). RNA-binding motif protein 15 binds to the RNA transport element RTE and provides a direct link to the NXF1 export pathway. J. Biol. Chem. *281*, 36915–36928.

Liu, N., Dai, Q., Zheng, G., He, C., Parisien, M., and Pan, T. (2015). N(6)-methyladenosine-dependent RNA structural switches regulate RNA-protein interactions. Nature *518*, 560–564.

Malik, S., and Roeder, R.G. (2010). The metazoan Mediator co-activator complex as an integrative hub for transcriptional regulation. Nat. Rev. Genet. *11*, 761–772.

Malovannaya, A., Lanz, R.B., Jung, S.Y., Bulynko, Y., Le, N.T., Chan, D.W., Ding, C., Shi, Y., Yucer, N., Krenciute, G., et al. (2011). Analysis of the human endogenous coregulator complexome. Cell *145*, 787–799.

McHugh, C.A., Chen, C.K., Chow, A., Surka, C.F., Tran, C., McDonel, P., Pandya-Jones, A., Blanco, M., Burghard, C., Moradian, A., et al. (2015). The Xist lncRNA interacts directly with SHARP to silence transcription through HDAC3. Nature *521*, 232–236.

Nesterova, T.B., Senner, C.E., Schneider, J., Alcayna-Stevens, T., Tattermusch, A., Hemberger, M., and Brockdorff, N. (2011). Pluripotency factor binding and Tsix expression act synergistically to repress Xist in undifferentiated embryonic stem cells. Epigenetics Chromatin *4*, 17.

Penny, G.D., Kay, G.F., Sheardown, S.A., Rastan, S., and Brockdorff, N. (1996). Requirement for Xist in X chromosome inactivation. Nature *379*, 131–137.

Ping, X.L., Sun, B.F., Wang, L., Xiao, W., Yang, X., Wang, W.J., Adhikari, S., Shi, Y., Lv, Y., Chen, Y.S., et al. (2014). Mammalian WTAP is a regulatory subunit of the RNA N6-methyladenosine methyltransferase. Cell Res. *24*, 177–189.

Plath, K., Fang, J., Mlynarczyk-Evans, S.K., Cao, R., Worringer, K.A., Wang, H., de la Cruz, C.C., Otte, A.P., Panning, B., and Zhang, Y. (2003). Role of histone H3 lysine 27 methylation in X inactivation. Science *300*, 131–135.

Savarese, F., Flahndorfer, K., Jaenisch, R., Busslinger, M., and Wutz, A. (2006). Hematopoietic precursor cells transiently reestablish permissiveness for X inactivation. Mol. Cell. Biol. *26*, 7167–7177.

Schwartz, S., Mumbach, M.R., Jovanovic, M., Wang, T., Maciag, K., Bushkin, G.G., Mertins, P., Ter-Ovanesyan, D., Habib, N., Cacchiarelli, D., et al. (2014). Perturbation of m6A writers reveals two distinct classes of mRNA methylation at internal and 5′ sites. Cell Rep. *8*, 284–296.

Shi, Y., Downes, M., Xie, W., Kao, H.Y., Ordentlich, P., Tsai, C.C., Hon, M., and Evans, R.M. (2001). Sharp, an inducible cofactor that integrates nuclear receptor repression and activation. Genes Dev. *15*, 1140–1151.

Silva, J., Mak, W., Zvetkova, I., Appanah, R., Nesterova, T.B., Webster, Z., Peters, A.H., Jenuwein, T., Otte, A.P., and Brockdorff, N. (2003). Establishment of histone h3 methylation on the inactive X chromosome requires transient recruitment of Eed-Enx1 polycomb group complexes. Dev. Cell *4*, 481–495.

Silva, J.M., Marran, K., Parker, J.S., Silva, J., Golding, M., Schlabach, M.R., Elledge, S.J., Hannon, G.J., and Chang, K. (2008). Profiling essential genes in human mammary cells by multiplex RNAi screening. Science *319*, 617–620.

Sims, D., Mendes-Pereira, A.M., Frankum, J., Burgess, D., Cerone, M.A., Lombardelli, C., Mitsopoulos, C., Hakas, J., Murugaesu, N., Isacke, C.M., et al. (2011). High-throughput RNA interference screening using pooled shRNA libraries and next generation sequencing. Genome Biol. *12*, R104.

Smeets, D., Markaki, Y., Schmid, V.J., Kraus, F., Tattermusch, A., Cerase, A., Sterr, M., Fiedler, S., Demmerle, J., Popken, J., et al. (2014). Three-dimensional super-resolution microscopy of the inactive X chromosome territory reveals a collapse of its active nuclear compartment harboring distinct Xist RNA foci. Epigenetics Chromatin *7*, 8.

Tang, Y.A., Huntley, D., Montana, G., Cerase, A., Nesterova, T.B., and Brockdorff, N. (2010). Efficiency of Xist-mediated silencing on autosomes is linked to chromosomal domain organisation. Epigenetics Chromatin *3*, 10.

Wutz, A., and Jaenisch, R. (2000). A shift from reversible to irreversible X inactivation is triggered during ES cell differentiation. Mol. Cell *5*, 695–705.

Wutz, A., Rasmussen, T.P., and Jaenisch, R. (2002). Chromosomal silencing and localization are mediated by different domains of Xist RNA. Nat. Genet. *30*, 167–174.

Yang, F., DeBeaumont, R., Zhou, S., and Näär, A.M. (2004). The activator-recruited cofactor/Mediator coactivator subunit ARC92 is a functionally important target of the VP16 transcriptional activator. Proc. Natl. Acad. Sci. USA *101*, 2339–2344.

Zhao, J., Sun, B.K., Erwin, J.A., Song, J.J., and Lee, J.T. (2008). Polycomb proteins targeted by a short repeat RNA to the mouse X chromosome. Science *322*, 750–756.

Cell Reports

Supplemental Information

A Pooled shRNA Screen Identifies Rbm15, Spen, and Wtap as Factors Required for Xist RNA-Mediated Silencing

Benoit Moindrot, Andrea Cerase, Heather Coker, Osamu Masui, Anne Grijzenhout, Greta Pintacuda, Lothar Schermelleh, Tatyana B. Nesterova, and Neil Brockdorff

Figure S1

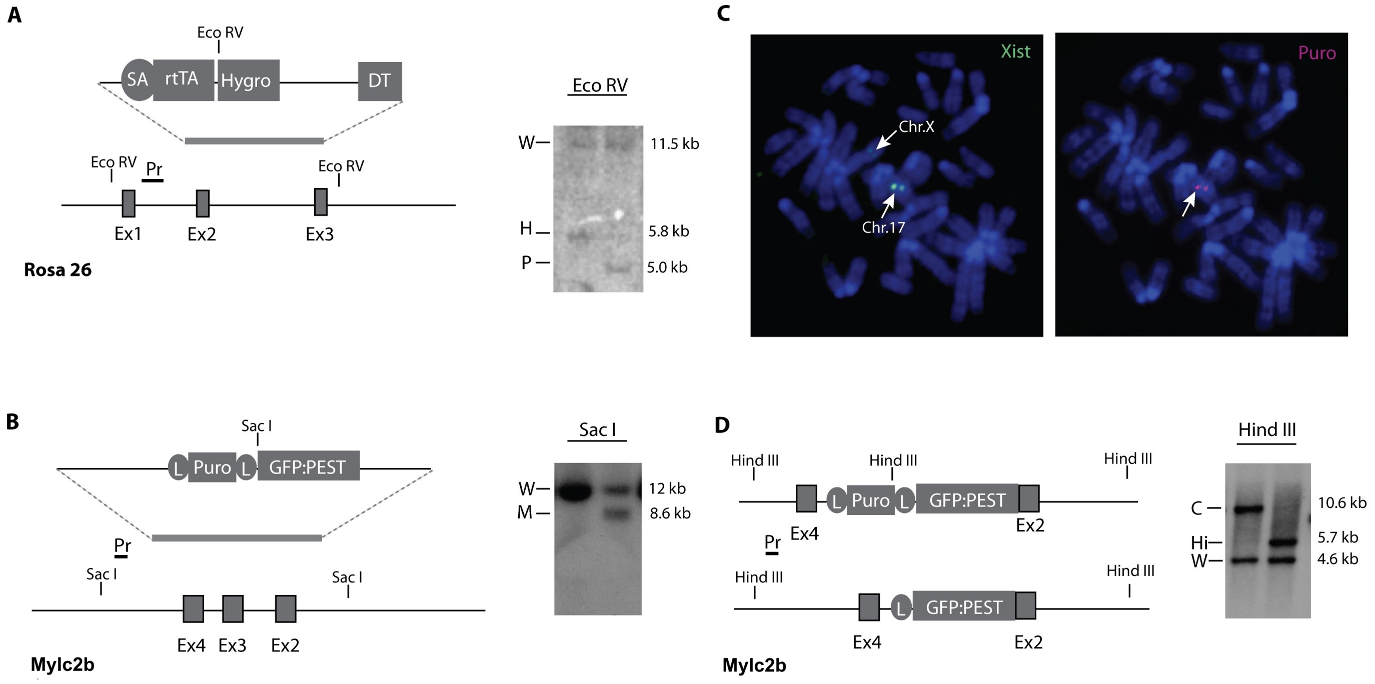
A
Eco RV
SA
rtTA
Hygro
DT
Eco RV
Pr
Eco RV
Ex1
Ex2
Ex3
Rosa 26
Eco RV
W
H
P
11.5 kb
5.8 kb
5.0 kb
C
Xist
Chr.X
Chr.17
Puro
B
Sac I
L
Puro
L
GFP:PEST
Pr
Sac I
Sac I
Ex4
Ex3
Ex2
Mylc2b
Sac I
W
M
12 kb
8.6 kb
D
Hind III
Hind III
Hind III
L
Puro
L
GFP:PEST
Ex4
Ex2
Pr
Hind III
Hind III
L
GFP:PEST
Ex4
Ex2
Mylc2b
Hind III
C
Hi
W
10.6 kb
5.7 kb
4.6 kb

Figure S2

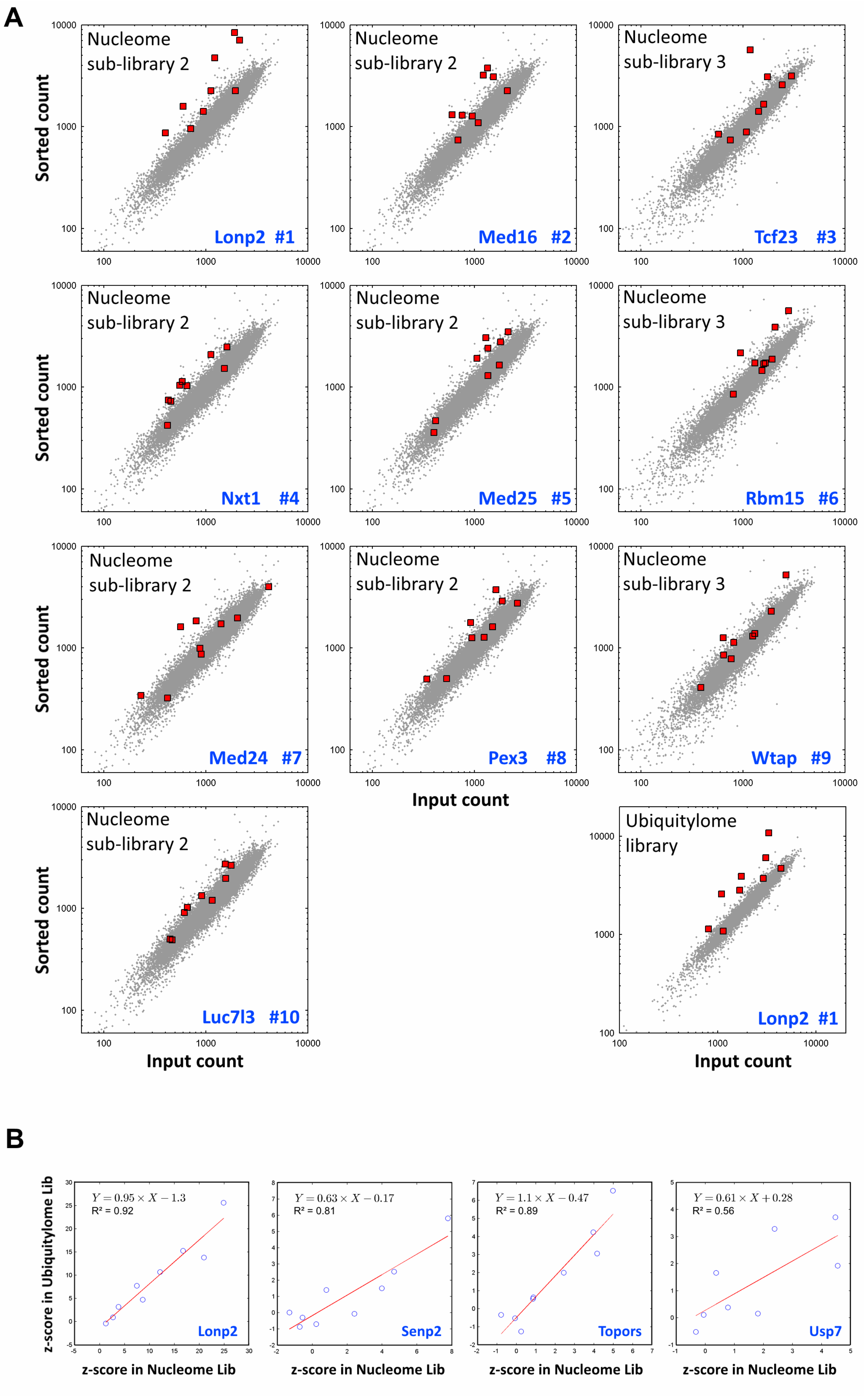
A
Sorted count
Input count
Nucleome sub-library 2
Lonp2 #1
Med16 #2
Nucleome sub-library 3
Tcf23 #3
Nxt1 #4
Med25 #5
Rbm15 #6
Med24 #7
Pex3 #8
Wtap #9
Luc7l3 #10
Ubiquitylome library
Lonp2 #1
B
z-score in Ubiquitylome Lib
z-score in Nucleome Lib
Y = 0.95 × X − 1.3
R² = 0.92
Lonp2
Y = 0.63 × X − 0.17
R² = 0.81
Senp2
Y = 1.1 × X − 0.47
R² = 0.89
Topors
Y = 0.61 × X + 0.28
R² = 0.56
Usp7

Figure S3

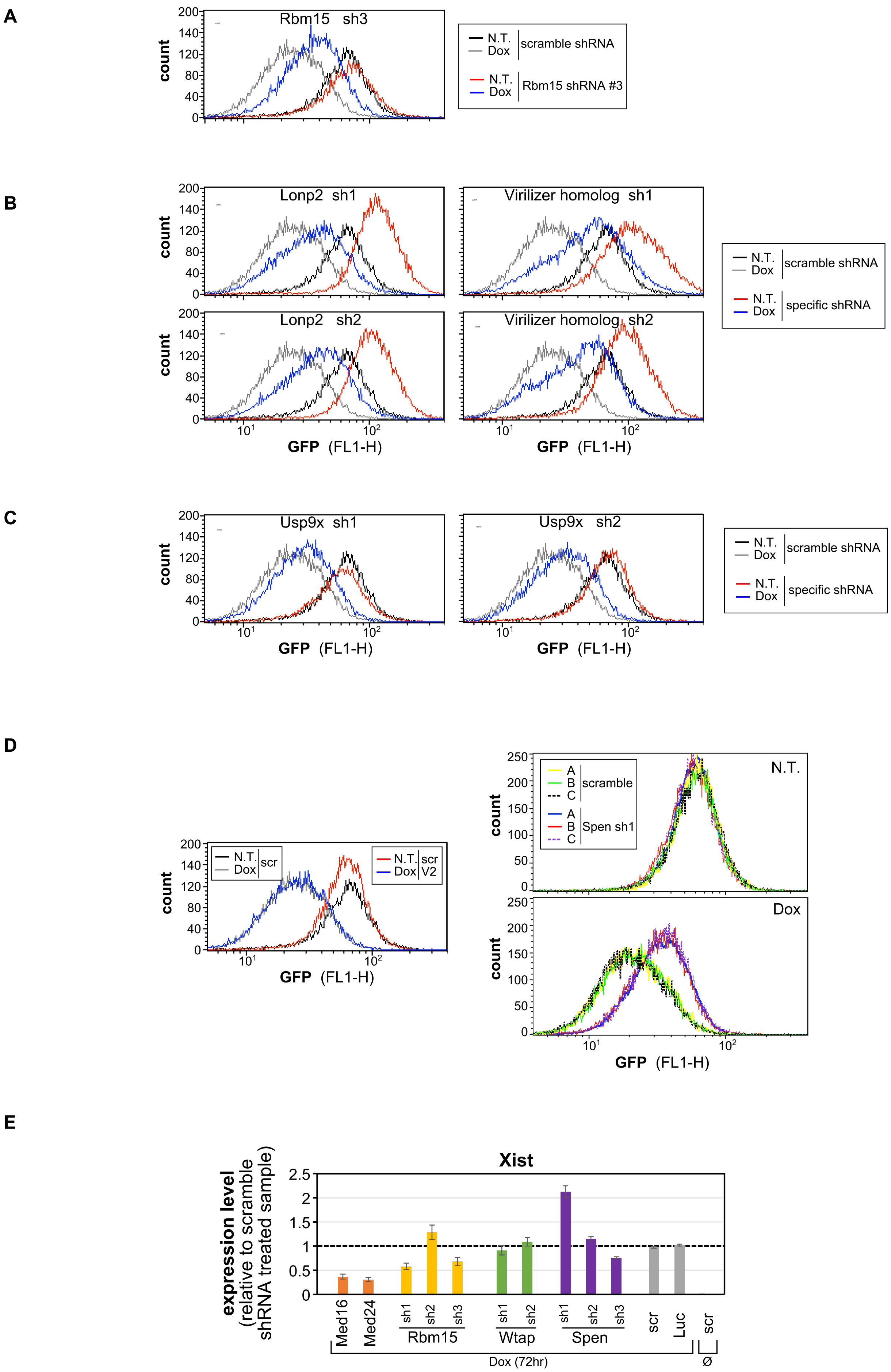

A
Rbm15 sh3
count
N.T. | Dox | scramble shRNA
N.T. | Dox | Rbm15 shRNA #3
B
Lonp2 sh1
Virilizer homolog sh1
Lonp2 sh2
Virilizer homolog sh2
N.T. | Dox | scramble shRNA
N.T. | Dox | specific shRNA
GFP (FL1-H)
C
Usp9x sh1
Usp9x sh2
N.T. | Dox | scramble shRNA
N.T. | Dox | specific shRNA
GFP (FL1-H)
D
N.T. | Dox | scr
N.T. | Dox | scr | V2
A B C | scramble
A B C | Spen sh1
N.T.
Dox
GFP (FL1-H)
E
Xist
expression level (relative to scramble shRNA treated sample)
Med16
Med24
sh1 sh2 sh3 Rbm15
sh1 sh2 Wtap
sh1 sh2 sh3 Spen
scr
Luc
scr
Dox (72hr)
Ø

Figure S4

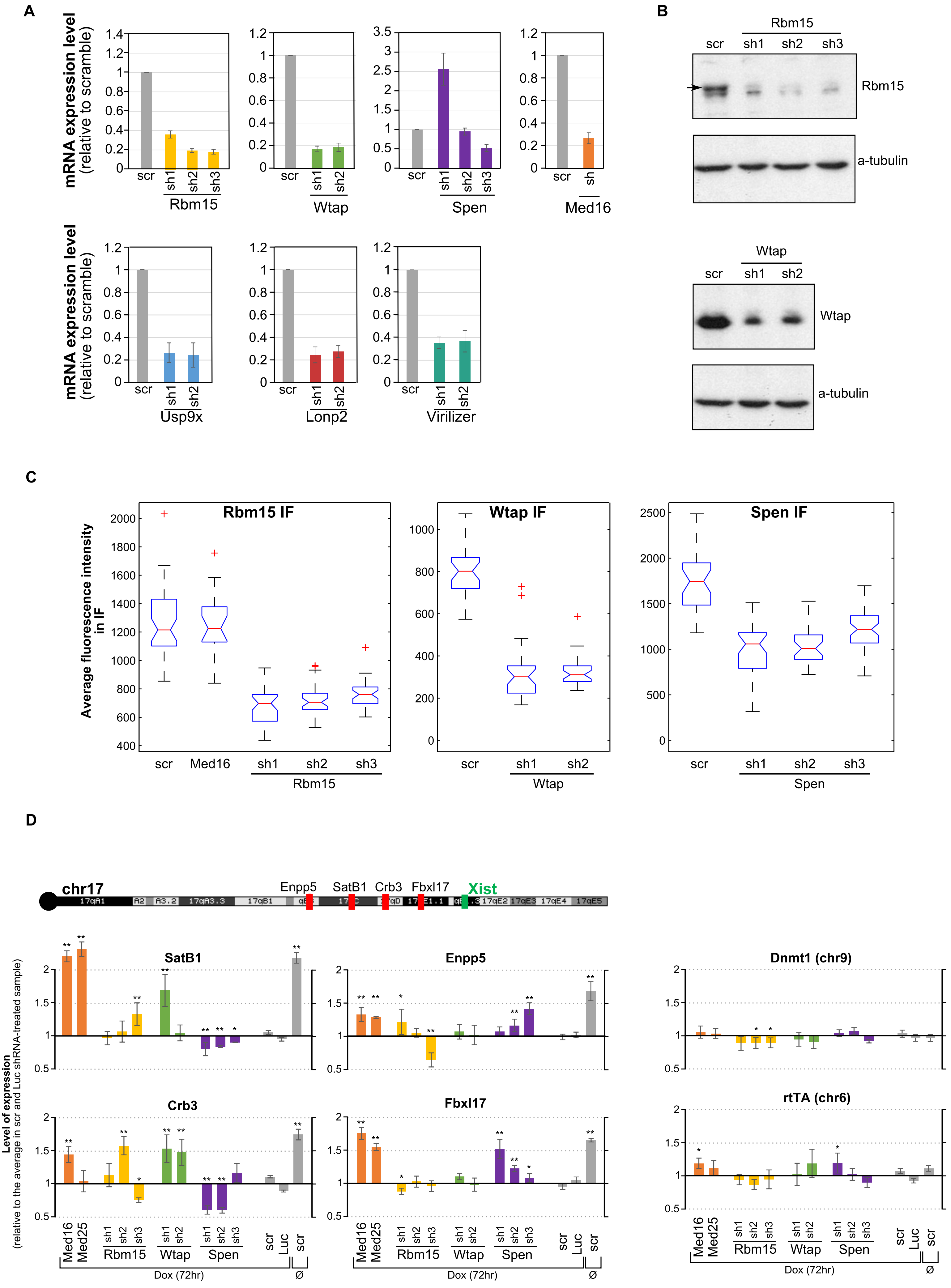

A
mRNA expression level (relative to scramble)
scr
sh1
sh2
sh3
Rbm15
Wtap
Spen
sh
Med16
Usp9x
Lonp2
Virilizer
B
Rbm15
Wtap
a-tubulin
C
Rbm15 IF
Wtap IF
Spen IF
Average fluorescence intensity in IF
Med16
D
chr17
Enpp5
SatB1
Crb3
Fbxl17
Xist
Dnmt1 (chr9)
rtTA (chr6)
Level of expression (relative to the average in scr and Luc shRNA-treated sample)
Med25
Luc
Dox (72hr)
Ø

Figure S5

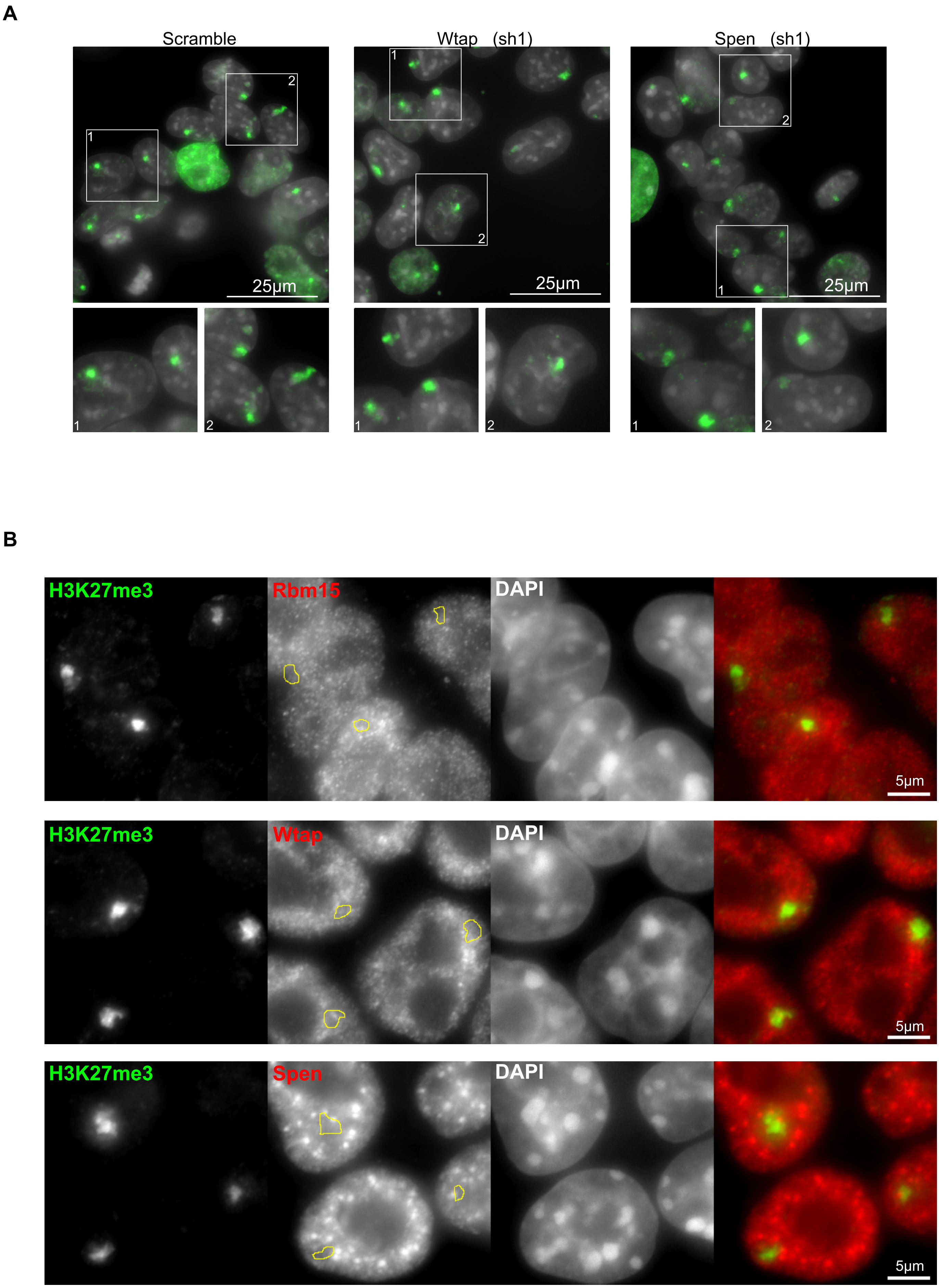
A
Scramble
Wtap (sh1)
Spen (sh1)
25μm
25μm
25μm
B
H3K27me3
Rbm15
DAPI
5μm
H3K27me3
Wtap
DAPI
5μm
H3K27me3
Spen
DAPI
5μm

Figure S6

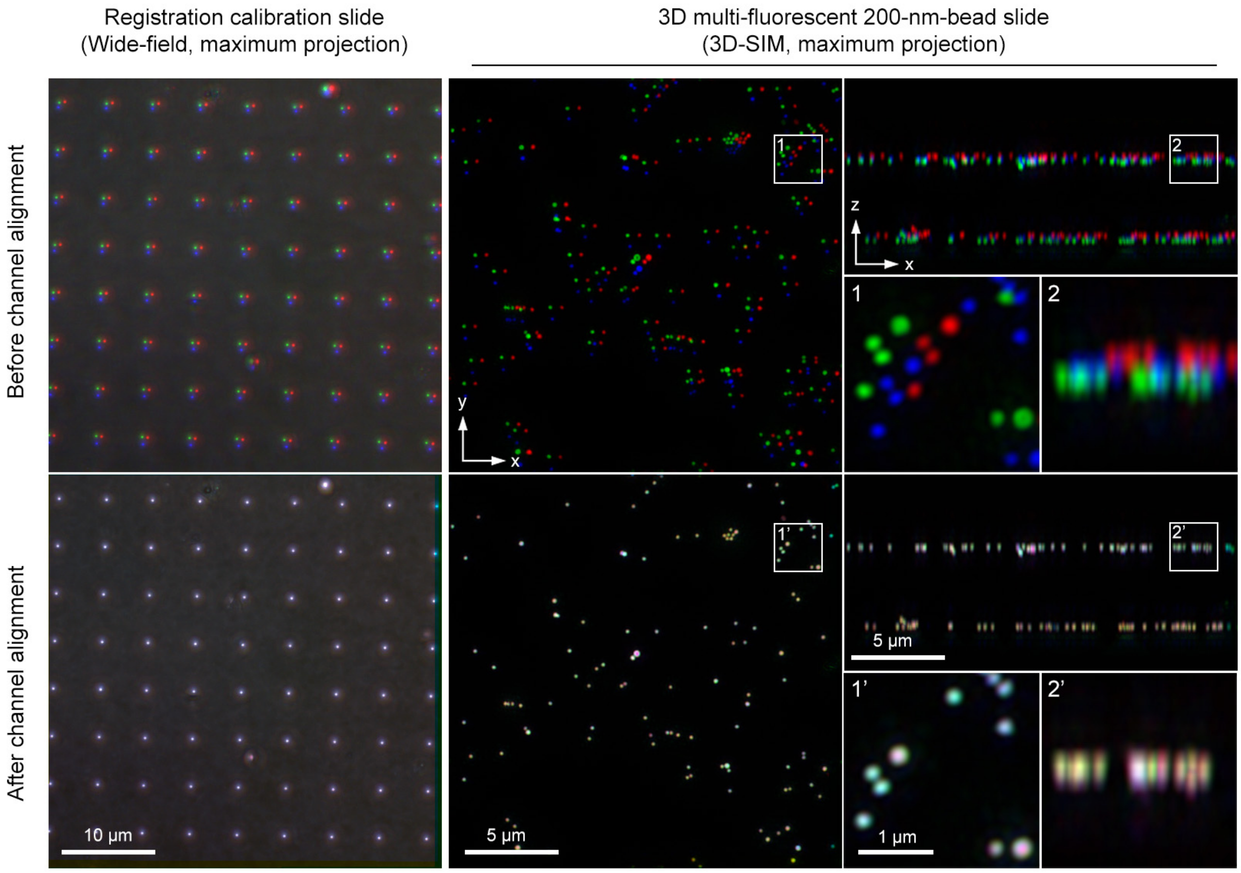

Registration calibration slide
(Wide-field, maximum projection)
3D multi-fluorescent 200-nm-bead slide
(3D-SIM, maximum projection)
Before channel alignment
After channel alignment
1
2
1'
2'
y
x
z
x
10 µm
5 µm
5 µm
1 µm

Supplementary Figure legends

Figure S1: Generation of the MG-3E reporter cell line, related to Figure 1.

A. Replacing puromycin cassette with a hygromycin cassette in Rosa26 locus. Schematic and Southern blot illustrate recombineering vector (top), WT allele (bottom). SA: splice acceptor, rtTA: reverse transactivator, Hygro: Hygromycin cassette, DT: Diphtheria Toxin cassette, Ex1-2-3: Exon 1-2-3, EcoRV: Restriction enzyme used for digestion, W: WT Rosa 26 allele, H: Hygromycin allele, P: Puromycin allele. Pr: probe. rtTA and Hygro have a polyA signal cassette at their 3’ end. Molecular weight of digested fragments is shown.

B. Targeting GFP:PEST construct into Mylc2b locus. Schematic and Southern blot illustrate recombineering vector (top), WT allele (bottom). Ex2-3-4: Exon 2-3-4, L: LoxP site, Puro: Puromycin cassette, GFP:PEST: unstable GFP cassette. SacI: RE used for digestion, W: WT DNA, M: Mylc2b targeted allele. Molecular weight of digested fragments is shown. Pr: probe. Puro and GFP:PEST cassette have a polyA signal at their 3’ end.

C. DNA FISH to verify that Xist and GFP:PEST are located *in cis* on chr17. Xist cDNA (green) and puro cassette (red) were used as probes. Chr. 17 and Chr. X are indicated.

D. Cre treatment of correctly targeted alleles to remove Puromycin cassette. Schematic and Southern blot illustrate targeted allele before (top) and after (bottom) Cre treatment. L: LoxP site, Puro: Puromycin cassette, GFP:PEST: unstable GFP, HindIII: RE used for digestion, W: WT DNA, Hi: HindIII + allele (before Cre treatment), C: Cre allele (after Puromycin cassette excised). Pr: probe. Molecular weight of digested fragments is shown. Schematics are not to scale.

Figure S2: Analysis of hairpin enrichment in GFP-high cells, related to Figure 2.

A. Distribution of shRNA scores for top 10 candidates from the nucleome screen. Each plot represents the hairpin count in unsorted and sorted MG-3E cells transduced with one of the three nucleome sub-libraries. The 9 hairpins targeting each candidate are shown in red. Some red dots are outliers in the upper left quadrant of the graph, indicating their enrichment in the FACS-sorted population. Lonp2 is ranked first in both the nucleome and ubiquitylome screens.

B. Screen reproducibility. Lonp2, Senp2, Topors, and Usp7 were identified in both the nucleome and ubiquitylome screens. The same hairpins were used in both libraries. We compared the enrichment (modified z-score) of each hairpin targeting one of those 4 genes in the two libraries. The strong correlation observed between these two independent libraries indicates a high degree of reproducibility.

Figure S3: Validation of top ranked targets, related to Figure 3.

A. GFP fluorescence of MG-3E reporter cell line with (grey/blue, 72hr) or without (black/red) doxycycline and transduced with scramble (black/grey) or Rbm15 sh3 shRNA.

B. Same than panel A but with shRNAs targeting Lonp2 or Virilizer Homolog. The knock-down of both genes increase the GFP brightness in the absence of doxycycline (Dox), indicating they affect GFP stability.

C. GFP fluorescence of MG-3E reporter cell line with (grey/blue, 72hr) or without (black/red) doxycycline (Dox) and transduced with scramble (black/grey) or specific shRNA targeting Usp9x. Usp9x was identified from the ubiquitylome screen (#3).

C. Reproducible scramble shRNA controls in MG-3E reporter cell line. Left: A second independent scrambled shRNA control does not affect the GFP fluorescence. Right: GFP-

fluorescence of the MG-3E reporter cell line transduced with scramble or Spen sh1 shRNA with (bottom) or without (top) doxycycline. The overlap between triplicates confirms good reproducibility of the approach.

D. Xist expression level following Med16, Med25, Rbm15, Wtap or Spen knockdown. Xist expression was induced for 72hr before RNA extraction (Dox 72hr). Expression levels are normalized to Actin, GAPDH and Idh2, and compared to the average expression levels for scrambled (scr) and Luciferase (Luc) shRNA-treated cells.

Figure S4: Knockdown efficiency and reduced silencing of chromosome 17 genes following knockdown of Rbm15, Wtap and Spen, related to Figure 4.

A. Relative expression level of Rbm15, Wtap, Spen, Med16, Usp9x, Lonp2 and Virilizer homologue in scrambled and specific shRNA-treated samples. Expression levels are normalized to Actin, GAPDH and Idh2. Bars represent mean ± SD in three experiments.

B. Western-blot for Rbm15 and Wtap in scrambled and target specific shRNA treated samples. Alpha Tubulin is used as a loading control.

C. Average intensity in immunofluorescence. Cells knocked-down for Rbm15, Wtap and Spen were seeded on slides and processed for IF using the corresponding antibodies. The average intensity was measured for more than 30 nuclei. The target specific shRNA treated cells showed reduced signal intensity relative to the scramble (scr) shRNA treated counterpart. Cells with Med16 knockdown, used as a control, that were stained using Rbm15 antibody show similar Rbm15 average fluorescence intensity than in the scramble shRNA treated cells.

D. RT-qPCR analysis of 4 genes located on chromosome 17 in Med16, Med25, Rbm15, Wtap and Spen knockdown 3E cells. Xist expression was induced for 72hr before RNA extraction (Dox 72hr). Expression levels are normalized to Actin, GAPDH and Idh2, and

compared to the average expression levels in scramble (scr) and Luciferase (Luc) shRNA treated cells. The expression of rtTA and Dnmt1, which are not on chromosome 17, are unaffected. The expression levels without doxycycline (ø) are also shown. The expected maximum variation is 2-fold as only one allele can be silenced by Xist. Bars represent mean ± SD in three experiments.

Figure S5: Rbm15, Wtap and Spen are not enriched within the inactive chromosome territory and their knockdown does not affect the H3K27me3 domains, related to Figures 5 and 6.

A. H3K27me3 domains are unaffected by knockdown of Wtap or Spen.

H3K27me3 domains in 3E cells treated with scramble, Wtap or Spen targeting shRNA. Xist was induced for 24hr before the IF. The inserts (below) show magnifications. Scale bars are 25µm.

B. Rbm15, Wtap and Spen are not enriched within the inactive chromosome territory.

3E cells were induced by doxycycline for 24hr and processed for immunofluorescence using Rbm15, Wtap and Spen antibodies. H3K27me3 staining was used to highlight the inactive chromosome territory.

Figure S6. Channel alignment for 3D-SIM co-localization analyses, related to Figure 6. Left column: conventional 3-channel acquisition of wide-field transmission illumination of special registration calibration slide (metal coated coverslip with microprocessed array of holes) before (top) and after (bottom) registration. Right column: Maximum intensity projection of 3D-SIM image stack of 200 nm diameter multi-fluorescent (TetraSpeck) beads attached to the coverslip (bottom), and slide (top) surface of the sample mounted

with 100% glycerol. Detailed lateral (ROIs 1 and 1’) and orthogonal (ROIs 2 and 2’) views confirm the accuracy of the alignment procedure in all spatial dimensions.

Table S4

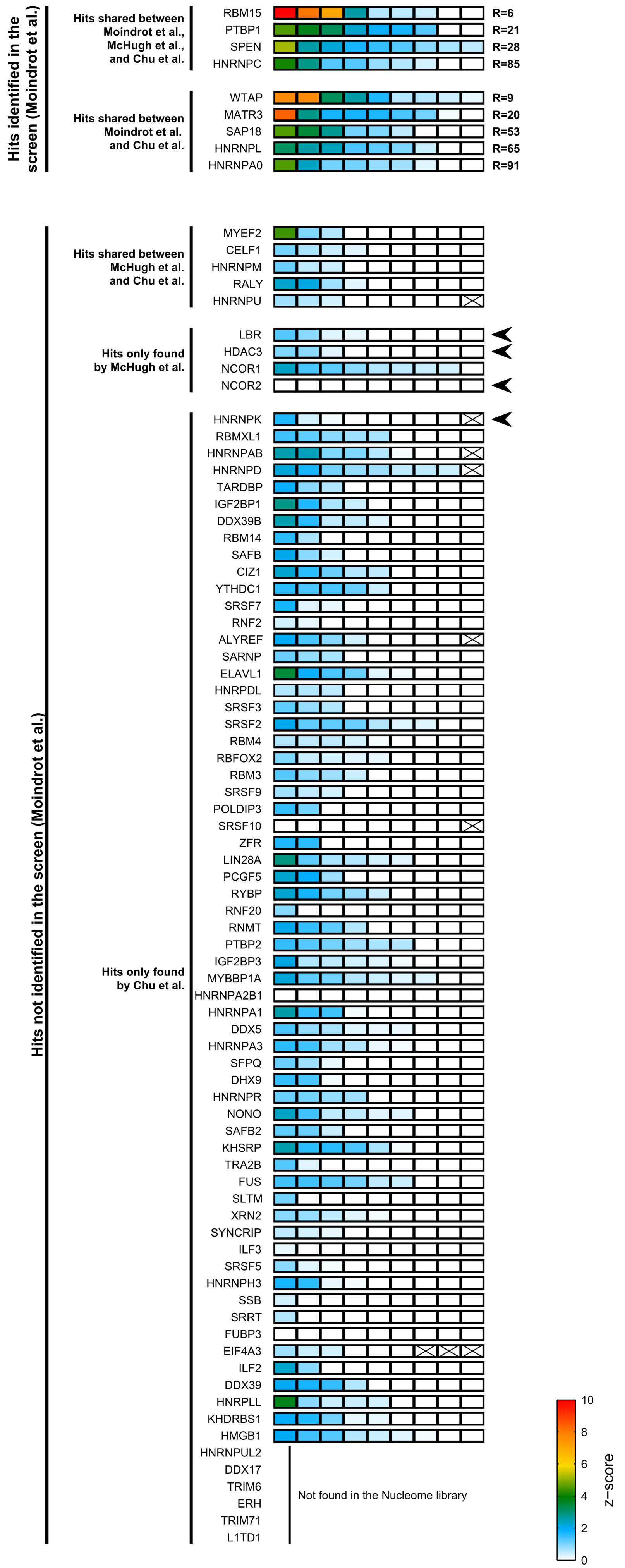

Hits identified in the screen (Moindrot et al.)
Hits shared between Moindrot et al., McHugh et al., and Chu et al.
RBM15
PTBP1
SPEN
HNRNPC
R=6
R=21
R=28
R=85
Hits shared between Moindrot et al. and Chu et al.
WTAP
MATR3
SAP18
HNRNPL
HNRNPA0
R=9
R=20
R=53
R=65
R=91
Hits not identified in the screen (Moindrot et al.)
Hits shared between McHugh et al. and Chu et al.
MYEF2
CELF1
HNRNPM
RALY
HNRNPU
Hits only found by McHugh et al.
LBR
HDAC3
NCOR1
NCOR2
Hits only found by Chu et al.
HNRNPK
RBMXL1
HNRNPAB
HNRNPD
TARDBP
IGF2BP1
DDX39B
RBM14
SAFB
CIZ1
YTHDC1
SRSF7
RNF2
ALYREF
SARNP
ELAVL1
HNRPDL
SRSF3
SRSF2
RBM4
RBFOX2
RBM3
SRSF9
POLDIP3
SRSF10
ZFR
LIN28A
PCGF5
RYBP
RNF20
RNMT
PTBP2
IGF2BP3
MYBBP1A
HNRNPA2B1
HNRNPA1
DDX5
HNRNPA3
SFPQ
DHX9
HNRNPR
NONO
SAFB2
KHSRP
TRA2B
FUS
SLTM
XRN2
SYNCRIP
ILF3
SRSF5
HNRNPH3
SSB
SRRT
FUBP3
EIF4A3
ILF2
DDX39
HNRPLL
KHDRBS1
HMGB1
HNRNPUL2
DDX17
TRIM6
ERH
TRIM71
L1TD1
Not found in the Nucleome library
10
8
6
4
2
0
z-score

Supplementary Table legends

Table S1: Unprocessed sequencing data

The excel file contains 4 sheets, one for the ubiquitylome, and 3 for the three nucleome libraries. The hairpin count after high-throughput sequencing in the plasmid sample (used to transfect the 293T cells), in the Input population and the FACS-sorted population are indicated for each hairpin.

Table S2: List of candidates from the nucleome screen.

The candidates are ranked based on the rescaled sum of the z-scores (RSZ). Columns: show the rank, Hugo name, the RefSeq ID of the targeted transcript, the RSZ, the total number of hairpin targeting this genes with more than 200 reads, the number of hairpins retained to calculate the RSZ, the number of positive hairpins (with a modified z-score above 2), the number of hairpins targeting this gene with a modified z-score above 3, between 2.5 and 3, and between 2 and 2.5, as well as the full name of the gene.

Table S3: List of candidates from the ubiquitylome screen.

Same as Table S2, but for the ubiquitylome library.

Table S4. Comparison of the hits from the genetic screen with recently published Xist RNA binding candidates identified in proteomics based studies (McHugh et al., 2015; Chu et al., 2015). The enrichment in the FACS-sorted cells (z-score) of every hairpin targeting a specific gene is represented using the same coloured heatmap as in Figure 2. Crosses indicate excluded hairpins for which read number was below a set threshold. The rank of the proteins identified in our genetic screen is indicated on the right side of the heatmaps. Arrowheads denote validated hits from the proteomics based studies.

Table S5: List of primers used for the RT-qPCR and the shRNA cloning into pLKO.1

Extended Experimental Procedures

Tissue culture

ES cells were grown on feeders (Puromycin resistant SNL) at 37°C 5% CO_2 in D-MEM (Life Technologies) supplemented with 10% fetal calf serum (Seralab), 2mM L-Glutamine (Life Technologies), 1X non-essential amino-acids (Life Technologies), 50µM 2-mercaptoethanol (Life Technologies), 1X Penicillin-Streptomycin (Life Technologies) and LIF-conditioned medium, made in-house, at a concentration equivalent to 1000 U/mL. To induce Xist expression, ES medium was supplemented with 1.5µg/mL doxycycline.

293T cells were grown at 37°C 5% CO_2 in D-MEM supplemented with 10% fetal calf serum, 2mM L-Glutamine, 1X non-essential amino-acids, 50µM 2-mercaptoethanol, 1X Penicillin-Streptomycin.

MG-3E Reporter cell line

A GFP reporter cell line was derived from the male 3E ES cell line (Tang et al., 2010) by DNA recombineering. A puromycin resistance cassette used to target the rtTA into Rosa26 locus in 3E cells was exchanged for a hygromycin resistance cassette. The coding region of Mylc2b, a gene previously shown to be sensitive to Xist silencing (Tang et al., 2010), was replaced by an unstable-GFP protein (GFP:PEST) (Corish and Tyler-Smith, 1999). Location of the GFP reporter *in cis* with the inducible Xist transgene was verified by DNA FISH using Xist cDNA and the puromycin resistance cassette as probes as described previously (Tang et al, 2010). Finally, the puromycin resistance cassette was excised from the Mylc2b locus by transient expression of CRE recombinase. MG-3E thus contains an inducible Xist transgene *in cis* with an unstable-GFP reporter, the expression of which is driven by the endogenous Mylc2b promoter, and is puromycin sensitive. Recombineering

steps were verified by Southern blot analysis using unique sequence probes as shown in Figure S1.

Lentivirus library

The nucleome library was designed to target all mouse genes with “Nucleus” gene ontology (GO:0005634) annotation. For the generation of a comprehensive mouse UPS list, GO searches were done for: “Ubiquitin Ligase Complex” GO-0000151, “Nuclear Ubiquitin Ligase Complex” GO-0000152, “Proteasome Complex” GO-0000502, “Ubiquitin-Specific protease activity” GO-00004843, “Proteasome Activator Complex” GO-00008537, “Protein Ubiquitination” GO-0016567, “Histone Ubiquitination” GO-0016574, “Protein Deubiquitination” GO-0016579, “Proteasome accessory complex” GO-0022624, “Nuclear Proteasome Complex” GO-0031595, “Proteosomal Ubiquitin-Dependent Protein Catabolic Process” GO-0043161, “C3HC4-type RING finger domain binding” GO-0055131, “RING-like Zinc Finger Domain Binding” GO-0071535, “Negative Regulation of Protein Deubiquitination”. This GO-cumulative list was updated with the addition of “RBX, SKP, CULLIN, F-BOX, DDB, BTB, HECT, U-BOX, RING FINGER-containing protein” by GO searches. Manual annotated human E1, E2 and E3 Ligases, Ligase substrates and deubiquitinating enzyme list were added from the hUbiquitome database (http://202.38.126.151/hmdd/hubi/, (Du et al., 2011)). A comprehensive unique-only UPS gene-symbol list was created. This list was then converted in a mouse MGI/Gene symbol list using the David tool online (http://david.abcc.ncifcrf.gov/conversion.jsp). The final list contains 1004 “bona fide” Ubiquitin proteasome Associated factors. shRNA design and production of pooled shRNA libraries were performed by Cellecta Inc.

Lentivirus production

Selected hairpins were cloned in pLKO.1 using the oligonucleotides listed in Table S5.

For individual hairpin lentivirus production, 293T cells were transfected in a 10cm-dish using 40µL of Lipofectamine 2000 by 4.5µg psPAX2 + 1.5µg pMD2.G + 6µg pLKO.1 containing the appropriate hairpin. The medium was replaced the following day, and lentivirus was collected 44hr and 52hr after transfection. Lentivirus containing medium was then filtered through a 0.45µm PVDF filter, aliquoted and snap frozen. For the pooled shRNA libraries, the same protocol was applied except that 35 (for each nucleome sub-library) or 20 (ubiquitylome library) 10-cm dishes of 293T were processed at the same time. Lentivirus containing medium was filtered through a 0.45µm PVDF filter and concentrated using Lenti-X Concentrator (CloneTech), following manufacturer's instructions.

Flow cytometry

Cells were washed with PBS, resuspended in PBS containing 3.5% fetal calf serum and 1.5µg/mL doxycycline. 30-50 million ES cells were analyzed by FACS (Beckman Coulter MoFlo XDP) for the ubiquitylome library and each of the 3 nucleome sub-libraries, and the 5% most fluorescent cells were sorted. 30,000 ES cells were analyzed (BD FACSCalibur), after transduction of individual hairpin.

DNA extraction for the shRNA barcode amplification and quantification

Input and Sorted cells (5% most GFP fluorescent cells) were processed as follows.

ES cells were lysed in 10mM NaCl, 10mM Tris pH7.5, 10mM EDTA, 0.5% Sarcosyl, 20µg/mL RNAseA. DNA was sheared by passing the lysate through a 23G needle. The lysate was then incubated overnight at 55°C in presence of 200µg/mL Proteinase K, phenol-chloroform extracted, and ethanol precipitated. DNA was then resuspended in 10mM Tris pH7.5 and quantified by Nanodrop. Barcode amplification, HTS, and barcode count was carried out by Cellecta Inc.

Candidate identification and ranking

For every hairpin we calculated its enrichment (Ei) in the Sorted 5% most fluorescent cells by dividing the hairpin count in the sorted sample by the hairpin count in the Input. We then calculated the Modified Z-score of each hairpin (ModZi)

$$ModZ_i = \frac{0.6745 \times (E_i - \widetilde{E})}{median\{|E_i - \widetilde{E}|\})}$$

where $\widetilde{E}$ is the sample median enrichment for the hairpin of a given (sub)-library.
We then combined the Modified Z-score of the 9 hairpins targeting the same genes and kept only those for which both the count in the input and sorted samples were > 200 reads. To generate the list of candidates, we limited for genes having at least one hairpin with a modified Z-score above 3 and at least one other hairpin with a modified Z-score above 2. We ranked this list of candidates according to the rescaled sum of the z-scores (RSZ) calculated on the 7 (out of 9) largest modified z-scores to account for potential off-target hairpins.

Immunofluorescence

Slides were washed with PBS, fixed with 2% formaldehyde in PBS for 15 min, permeablised with 0.4% Triton X-100 in PBS for 5 min and then blocked with 0.2% fish skin gelatin (Sigma) in PBS (30-60min). Primary antibodies were used as follows: H3K27me3 (1:500 ActiveMotif #61017), Rbm15 (1:100 ProteinTech 10587-1-AP), Wtap (1:200 ProteinTech 10200-1-AP), Spen (1:250 Abcam ab72266). Secondary Antibodies were Alexa-fluor conjugated (1:400 Life Technologies). Slides were mounted in Vectashield with DAPI and visualised using a 63x oil immersion objective with the Zeiss Axio Observer Z1 microscope. Images were analysed with ImageJ. H3K27me3 domains were segmented using 3D spot segmentation plugin for ImageJ (Ollion et al., 2013).

Strong and weak domains are automatically detected by the plugin, with strong domain having a mean intensity > 900. Weak domains were detected by eye only.

RNA FISH

1µg Xist cDNA was labelled with green-dUTP (Abbott Molecular) using a nick translation kit (Abbott Molecular) in a total volume of 50µL. For every 22x22mm coverslip, 2.5µL of labelled probe were ethanol precipitated with 1µg of Salmon Sperm DNA (Life Technologies), and resuspended in 12µL of 50% formamide, 2X SSC, 10% dextran sulfate, 1mg/mL BSA. Coverslips were washed with PBS, fixed with 2.6% formaldehyde in PBS for 10min at room temperature and permeabilized with 0.42% Triton X-100 in PBS for 5min on ice. Coverslips were then washed with PBS, dehydrated with 70%-80%-95%-100% ethanol, and hybridized overnight at 37°C in humid chamber. The following day, the coverslips were washed 3 times with 50% formamide, 2X SSC at 42°C, and 3 times with 2X SSC at 42°C.Coverslips were mounted in Vectashield with DAPI and visualised using a 63x oil immersion objective with the Zeiss Axio Observer Z1 microscope.

For Fbxl17 nascent RNA-Fish, 1µg RP24-324P16 BAC was labelled using Red-dUTP (Abbott Molecular) using a nick translation kit (Abbott Molecular) in a total volume of 50µL. For every 22x22mm coverslip, 1.5µL of labelled Xist and 2.5µL of labelled BAC were ethanol precipitated with 1µg of Salmon Sperm DNA and 3µg of mouse Cot-1 (Life Technologies), denaturated for 5min at 75°C in 50% formamide, 2X SSC, 10% dextran sulfate, 1mg/mL BSA and competed for 30min at 37°C.

Rnf12 and Pgk1 nascent RNA-FISH was performed in differentiated female ES cells. Feederless XT67E1 XX ES cell line (Penny et al., 1996) was grown on gelatinized plates in D-MEM medium supplemented with LIF. Individual hairpin knockdowns were performed as described above, and stable puromycin-resistant populations of XT67E1 cells were selected for 5-6 days. To induce differentiation, XT67E1 cells were trypsinised and seeded

at low density (1.5x10^4 cells/cm^2) on non-gelatinized plates in D-MEM medium without LIF. After 5 days of differentiation cells were trypsinised, plated on coverslips and left in differentiation conditions overnight. The following day (day 6 of differentiation) coverslips were rinsed in PBS and fixed in 3.7% formaldehyde for 10 min at room temperature. Cells were permeabilised with 0.5% Triton X100 in CSK buffer for 5 min on ice (Clemson et al., 1996), rinsed in PBS and stored in 70% ethanol at +4˚C until use. Rnf12 and Pgk1 nascent transcripts were visualized with 48 (Rnf12) or 96 (Pgk1) Quasar® 570-labelled oligonucleotides designed against uniqueintronic sequences using the online Stellaris® Probe Designer tool from BioSearch Technologies (USA). For every coverslip, 1µL of Spectrum-Green labeled Xist probe was resuspended in BioSearch Technologies hybridization buffer, denatured for 5min at 75°C and kept on ice until use. The Quasar® 570-labelled oligonucleotide probes were mixed with Xist probe just before the hybridization (250nM final concentration). Hybridization and washes were done following the manufacturer’s instructions. Coverslips were visualised using a 63x oil immersion objective with the Zeiss Axio Observer Z1 microscope.

Structured illumination microscopy

Cells for SR-3DSIM were seeded onto No. 1.5H (170 µm ± 5 µm) coverslips (Marienfield) and BglSL-Xist expression was induced with doxycycline for 24hrs. Cells were fixed with 2% formaldehyde for 10 min, permeabilised in 0.5% Triton X-100 PBS for 10 minutes. Initial blocking (30 min) and all further antibody incubations were carried out in 3% BSA, 5% normal goat serum (Sigma), 5% normal donkey serum (Santa Cruz) and 0.5% fish gelatine in PBS with 0.05% tween-20. Coverslips were incubated with primary antibody for 1 h in a humidified chamber (rabbit anti-Rbm15, 1/100 Proteintech 10587-1-AP, Rabbit anti-Spen, 1/1000 Atlas antibodies HPA015825, Rabbit anti-Wtap, 1/500 Proteintech 10200-1-AP, Rabbit anti-Ezh2, 1/200 Cell Signalling, and sheep anti-mCherry (kind gift of

Francis Barr) 1/500. After extensive washing in PBS-T, coverslips were incubated with Alexa Fluor goat anti-rabbit 488 and Alexa Fluor donkey anti-sheep 568, 1/1000 for 30min. Cells were again extensively washed, then post fixed with 2% formaldehyde before incubation with 2µg/ml DAPI for 10 minutes.

Super-resolution 3D-SIM was performed on a DeltaVision OMX V3 Blaze system (GE Healthcare) equipped with a 60x/1.42 NA PlanApo oil immersion objective (Olympus), sCMOS cameras (PCO), and 405, 488 and 593nm lasers. 3D-SIM image stacks were acquired with a z-distance of 125nm and with 15 raw images per plane (5 phases, 3 angles). The raw data was computationally reconstructed with SoftWoRx 6.1 (GE Healthcare) using channel-specifically measured optical transfer functions (OTFs) and Wiener filter settings 0.0020 (green/red channel) and 0.0040 (blue channel), respectively, to generate 3D stacks with ~120nm lateral and ~300nm axial resolution. Spherical aberration was minimized by matching the refractive indices (RI) of the immersion oil for sample acquisition (RI 1.514) and for generation OTFs (RI 1.512 for the blue and green, and 1.514 for the red OTFs) generated from ~100nm diameter blue (FluoroMax; Thermo Scientific), green and red (FluoSpheres; Life Technologies) beads, respectively. Lateral colour channel alignment was performed using a special image registration slide and algorithm provided by GE Healthcare. Correct 3D alignment was confirmed and refined in z by a custom test sample with two layers of 0.2µm diameter TetraSpeck beads (Life Technologies) (Figure S6). The full-scale 32-bit reconstructed data was thresholded for each channel to the stack modal grey value (representing the centre of the background intensity level) and converted to 16-bit composite tif-stacks using an in-house script in ImageJ (http://rsbweb.nih.gov/ij) before further processing.

Gene expression analysis

RNA was extracted with TRIzol® Reagent (Life Technologie) following manufacturer's instructions. The DNAse treatment was with TURBO DNA-free™ Kit (Life Technologies). The Reverse Transcription reaction was carried on 2µg RNA using SuperScript® III Reverse Transcriptase (Life Technologies). Quantitative PCR assays were carried out using the Rotor-Gene Q (Qiagen) and the iQ SYBR GREEN Supermix (BioRad) using the primers listed in Table S5.

Xist RNA tagging with Bgl-mCherry

A tandem array of 18 repeats of Bgl stem loop (BglSL) motif was inserted at a unique Bsu36I restriction site in the inducible Xist cDNA construct. The modified construct was then transfected into ES cells together with a plasmid encoding the fusion between the N-terminal RNA recognition motif of BglG and mCherry protein (Declerck et al., 2002; Chen et al., 2009). Stable cell lines were screened to identify lines with tagged Xist expression after 24hr of doxycycline treatment.

Supplemental references

Chen, J., Nikolaitchik, O., Singh, J., Wright, A., Bencsics, C. E., Coffin, J. M., Ni, N., Lockett, S., Pathak, V. K. and Hu, W.-S. (2009). High efficiency of HIV-1 genomic RNA packaging and heterozygote formation revealed by single virion analysis. Proc Natl Acad Sci U S A *106*, 13535–13540.

Chu, C., Zhang, Q. C., da Rocha, S. T., Flynn, R. A., Bharadwaj, M., Calabrese, J. M., Magnuson, T., Heard, E. and Chang, H. Y. (2015). Systematic discovery of Xist RNA binding proteins. Cell *161*, 404–416.

Clemson, C. M., McNeil, J. A., Willard, H. F. and Lawrence, J. B. (1996). XIST RNA paints the inactive X chromosome at interphase: evidence for a novel RNA involved in nuclear/chromosome structure. J Cell Biol *132*, 259–275.

Corish, P. and Tyler-Smith, C. (1999). Attenuation of green fluorescent protein half-life in mammalian cells. Protein Eng *12*, 1035–1040.

Declerck, N., Minh, N. L., Yang, Y., Bloch, V., Kochoyan, M. and Aymerich, S. (2002). RNA recognition by transcriptional antiterminators of the BglG/SacY family: mapping of SacY RNA binding site. J Mol Biol *319*, 1035–1048.

Du, Y., Xu, N., Lu, M. and Li, T. (2011). hUbiquitome: a database of experimentally verified ubiquitination cascades in humans. Database (Oxford) *2011*, bar055.

McHugh, C. A., Chen, C.-K., Chow, A., Surka, C. F., Tran, C., McDonel, P., Pandya-Jones, A., Blanco, M., Burghard, C., Moradian, A., Sweredoski, M. J., Shishkin, A. A., Su, J., Lander, E. S., Hess, S., Plath, K. and Guttman, M. (2015). The Xist lncRNA interacts directly with SHARP to silence transcription through HDAC3. Nature *521*, 232–236.

Ollion, J., Cochennec, J., Loll, F., Escudé, C. and Boudier, T. (2013). TANGO: a generic tool for high-throughput 3D image analysis for studying nuclear organization. Bioinformatics *29*, 1840–1841.

Penny, G. D., Kay, G. F., Sheardown, S. A., Rastan, S. and Brockdorff, N. (1996). Requirement for Xist in X chromosome inactivation. Nature *379*, 131–137.

Tang, Y. A., Huntley, D., Montana, G., Cerase, A., Nesterova, T. B. and Brockdorff, N. (2010). Efficiency of Xist-mediated silencing on autosomes is linked to chromosomal domain organisation. Epigenetics Chromatin *3*, 10.
